# LINC00330/CCL2 axis-mediated ESCC TAM reprogramming affects tumor progression

**DOI:** 10.1186/s11658-024-00592-8

**Published:** 2024-05-20

**Authors:** Lijun Zhao, Gengchao Wang, Haonan Qi, Lili Yu, Huilong Yin, Ruili Sun, Hongfei Wang, Xiaofei Zhu, Angang Yang

**Affiliations:** 1 Henan Key Laboratory of Immunology and Targeted Drugs, Xinxiang Key Laboratory of Tumor Microenvironment and Immunotherapy, School of Medical Technology, Xinxiang Medical University, Xinxiang, Henan China; 2 State Key Laboratory of Liver Research, Department of Pathology, Li Ka Shing Faculty of Medicine, The University of Hong Kong, Hong Kong, China; 3 The State Key Laboratory of Cancer Biology, Department of Immunology, Fourth Military Medical University, Xi’an, Shanxi China

**Keywords:** ESCC, TAM reprogramming, LINC00330, CCL2

## Abstract

**Background:**

Tumor-associated macrophages (TAMs) significantly influence the progression, metastasis, and recurrence of esophageal squamous cell carcinoma (ESCC). The aberrant expression of long noncoding RNAs (lncRNAs) in ESCC has been established, yet the role of lncRNAs in TAM reprogramming during ESCC progression remains largely unexplored.

**Methods:**

ESCC TAM-related lncRNAs were identified by intersecting differentially expressed lncRNAs with immune-related lncRNAs and performing immune cell infiltration analysis. The expression profile and clinical relevance of LINC00330 were examined using the TCGA database and clinical samples. The LINC00330 overexpression and interference sequences were constructed to evaluate the effect of LINC00330 on ESCC progression. Single-cell sequencing data, CIBERSORTx, and GEPIA were utilized to analyze immune cell infiltration within the ESCC tumor microenvironment and to assess the correlation between LINC00330 and TAM infiltration. ESCC-macrophage coculture experiments were conducted to investigate the influence of LINC00330 on TAM reprogramming and its subsequent effect on ESCC progression. The interaction between LINC00330 and C–C motif ligand 2 (CCL2) was confirmed through transcriptomic sequencing, subcellular localization analysis, RNA pulldown, silver staining, RNA immunoprecipitation, and other experiments.

**Results:**

LINC00330 is significantly downregulated in ESCC tissues and strongly associated with poor patient outcomes. Overexpression of LINC00330 inhibits ESCC progression, including proliferation, invasion, epithelial–mesenchymal transition, and tumorigenicity in vivo. LINC00330 promotes TAM reprogramming, and LINC00330-mediated TAM reprogramming inhibits ESCC progression. LINC00330 binds to the CCL2 protein and inhibits the expression of CCL2 and downstream signaling pathways. CCL2 is critical for LINC00330-mediated TAM reprogramming and ESCC progression.

**Conclusions:**

LINC00330 inhibited ESCC progression by disrupting the CCL2/CCR2 axis and its downstream signaling pathways in an autocrine fashion; and by impeding CCL2-mediated TAM reprogramming in a paracrine manner. The new mechanism of TAM reprogramming mediated by the LINC00330/CCL2 axis may provide potential strategies for targeted and immunocombination therapies for patients with ESCC.

**Graphical Abstract:**

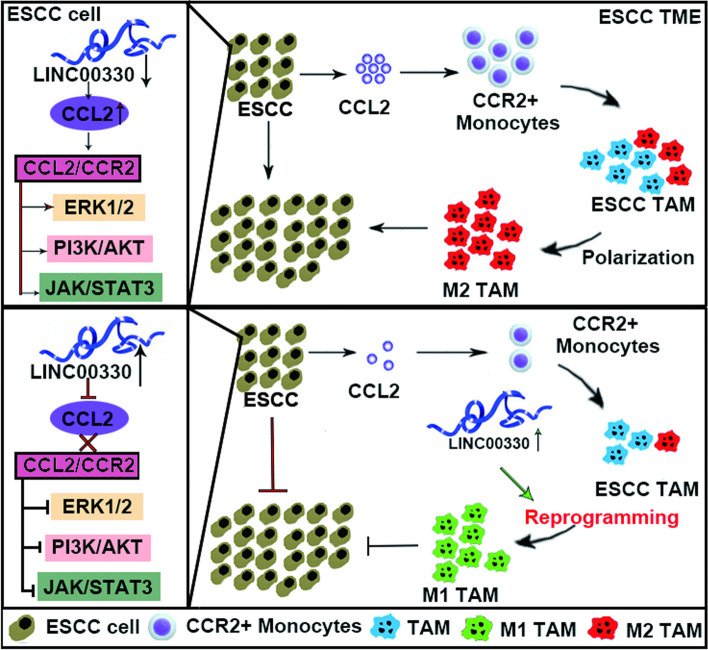

**Supplementary Information:**

The online version contains supplementary material available at 10.1186/s11658-024-00592-8.

## Background

Esophageal squamous cell carcinoma (ESCC) is the predominant subtype of esophageal cancer, accounting for approximately 90% of cases worldwide and more than 95% of cases in China [1]. ESCC is notoriously challenging to treat because it is associated with high morbidity and mortality rates, a grim prognosis, and an elevated risk of recurrence [2]. Recent advances have shifted the management of ESCC toward precision medicine, immunotherapy, and targeted therapy, disrupting the conventional triad of surgery, radiotherapy, and chemotherapy and offering new hope for ESCC patients [3]. However, the prognosis for most ESCC patients remains dismal [4].

Recent studies have increasingly demonstrated that the tumor microenvironment (TME) is pivotal in the progression of malignancies, including ESCC [5, 6]. Tumor-associated macrophages (TAMs) are prevalent immune cell infiltrates within the TME and play pivotal roles in tumor progression, metastasis, recurrence and immune evasion [7–9]. TAMs are highly plastic in phenotype and function and are influenced by TME to differentiate into various subtypes, predominantly M1 and M2 [10]. The quantity and ratio of TAM polarization phenotypes vary with different stages of tumor development [11]. The majority of TAMs in the TME of solid tumors, including ESCC [12], predominantly exhibit M2-like characteristics [13]. The substantial infiltration of M2 TAMs at tumor sites is closely associated with histological grade, tumor invasion depth, vascular infiltration, lymph node metastasis, clinical stage, and poor prognosis in patients with ESCC [14, 15]. Hence, identifying novel biomarkers that can induce TAM reprogramming within the ESCC TME represents a promising therapeutic avenue.

Long noncoding RNAs (lncRNAs) are a class of molecules that, despite not coding for proteins, have biological functions [16]. LncRNAs are dysregulated in various human diseases, including ESCC, and their dysregulation is intimately linked to disease pathogenesis and progression, suggesting potential clinical applications [17]. Recently, numerous studies have established that lncRNAs are essential for modulating TME remodeling and macrophage function [18–20]. Manipulating macrophage lncRNA activity may offer therapeutic benefits, potentially ameliorating disease severity and reducing morbidity [21, 22]. LINC00330, an intergenic noncoding RNA, has been shown to enhance the migration, invasion, and survival of laryngeal cancer cells in vitro by activating the PI3K/Akt signaling pathway [23]. It appears to be a pivotal element in the onset and progression of bladder cancer and has potential as a prognostic biomarker for the disease [24]. However, the precise functions and mechanisms of LINC00330 in the progression of TAM and ESCC require detailed characterization.

In this study, we identified LINC00330 as an ESCC TAM-associated lncRNA with a tumor suppressive effect. LINC00330 can specifically bind to the C–C motif ligand 2 (CCL2) protein. The LINC00330/CCL2 axis can block the CCL2/CCR2 axis and its downstream signaling pathways through the autocrine pathway and inhibit the secretion of CCL2 through the paracrine pathway, mediate TAM reprogramming, and inhibit ESCC progression. This research proposes a novel mechanism of TAM reprogramming through the LINC00330/CCL2 axis, offering a potential avenue for targeted and combination immunotherapy strategies for the treatment of ESCC patients.

## Methods

### Data collection and preprocessing

Gene expression data for ESCC were obtained from The Cancer Genome Atlas (TCGA) and Gene Expression Omnibus (GEO) datasets GSE53625, GSE130078, and GSE45670. Differential gene expression (DGE) analysis between cancer and normal samples was performed using the R packages: limma (version 3.52.1) for microarray data and DESeq2 (version 1.36.0) for RNA-seq raw count data. For the GSE53625 dataset, reference sequences from RefSeq and lncRNA gene sequences from LNCipedia (version 5.2) were used as reference databases to map gene information from the probe sequences of the GPL18109 platform via BLASTN (version 2.10.1). For the GSE130078 and TCGA datasets, the read counts were determined as previously described and acquired from the GEO and UCSC Xena databases for DGE analysis. The lncRNA gene were annotated using lncRNA gene annotations from GENCODE, and 164, 915, 1982, and 41 differentially expressed lncRNAs were identified in the GSE45670, GSE53625, GSE130078, and TCGA datasets, respectively (log2|fold change|> 0.585 and FDR < 0.05, Additional file 1: P1–4). Finally, we defined the significantly differentially expressed lncRNAs in at least two datasets (266 upregulated, 123 downregulated, a total of 389 significantly differentially expressed lncRNAs, Additional file 1: P5–6) as ESCC DE-lncRNAs for subsequent analysis.

### Selection of ESCC TAM associated lncRNAs

Immune-related genes were downloaded from ImmLnc (http://biobigdata.hrbmu.edu.cn/ImmLnc/), Gene Set Enrichment Analysis (GSEA) Molecular Signature Database v7.0. Then, 932 immune-associated lncRNAs (Imm-lncRNAs) were extracted from the GENCODE database on the basis of lncRNA gene annotations. The intersection of these 932 Imm-lncRNAs with the 389 DE-lncRNAs resulted in 99 ESCC immune-related DE-lncRNAs (ESCC Imm-lncRNAs), with 64 significantly upregulated and 35 significantly downregulated (Additional file 1: P8–9). Subsequently, ImmLnc software was used to perform an immune-relatedness analysis of these 99 ESCC Imm-lncRNAs, focusing primarily on the correlation between lncRNA expression and the infiltration of six classic immune cell types in ESCC, such as CD8 T cells, dendritic cells, neutrophils, CD4 T cells, B cells, and macrophages. This analysis identified 11 lncRNAs that were significantly correlated with TAM infiltration (|*r*|> 0.3, *P* < 0.05; Additional file 1: P12). Among them, six lncRNAs (two upregulated, NR2F1-AS1 and ZFHX4-AS1; four downregulated, HAND2-AS1, LINC00330, RP11-834C11.4, and TTTY10) were associated only with TAM infiltration, had no or weak correlations with other immune cell infiltrations, and were defined as ESCC TAM-associated lncRNAs.

### Clinical samples and cell lines

ESCC tissues and matched adjacent nontumor esophageal tissues were obtained from 22 patients who were newly diagnosed and histologically confirmed with ESCC by more than three pathologists at the Department of Pathology, The First Affiliated Hospital of Xinxiang Medical College, between January 2020 and December 2021. All enrolled patients signed a written informed consent form and none of the patients underwent preoperative chemotherapy or radiotherapy. Fresh cancer tissues and matched adjacent nontumor tissues were immediately collected after surgery and stored at −80 °C. The research methods were in accordance with the standards of the Helsinki Declaration, and the study was approved by the Ethics Committee of Xinxiang Medical College (No. XYLL-2021043).

SHEE (#IM-H382, IMMOCELL), Kyse450 (#CBP60458, COBIOER), and TE7 (#CBP60470, COBIOER) cells were donated by Henan Cancer Hospital. EC109 (#1101HUM-PUMC000246) cells were obtained from the Cell Resource Center, Peking Union Medical College. EC9706 (#IM-H270, IMMOCELL) and Kyse30(#IM-H311, IMMOCELL) were purchased from IMMOCELL. 293T (GNHu17) cells were obtained from the Shanghai Institute of Life Science Cell Bank Center (Shanghai, China). The Kyse450, EC109, and Kyse30 cell lines were maintained in Roswell Park Memorial Institute medium (RPIM)-1640 (#31800-500, Solarbio) supplemented with 10% fetal bovine serum (FBS; #10270-106, Gibco) and 1% penicillin–streptomycin (#C100C5, NCM Biotech). The 293T, TE-7, EC9706, and SHEE cell lines were maintained in Dulbecco’s modified Eagle’s medium (DMEM; #12100, Solarbio) supplemented with 10% FBS (#10270-106, Gibco) and 1% penicillin–streptomycin (#C100C5, NCM Biotech). The THP-1 cell line (#CL-0233, Proser) was purchased from Prosac and stored in THP-1 special medium (#CM-023, Proser). All cells were cultured at 37 °C in an incubator containing 5% CO_2_.

### Model of macrophage polarization

THP-1 cells were differentiated into intermediate-stage M0 cells by treatment with 300 nM phorbol 12-myristate 13-acetate (PMA, #S7791, Selleckchem) for 48 h. Subsequently, M1 and M2 macrophages were induced with 20 ng/mL IFN-γ (#AF-345-05-20 µG, Proteintech) for 48 h and 20 ng/mL IL-4 (#AF-214-14-5 µG, Proteintech), respectively, for 48 h on the basis of M0 macrophages. For ESCC TAMs, M0, M1, and M2 macrophages were coincubated with conditioned medium produced by ESCC cells. Note: THP-1 cells are oval suspension cells, M0 TAMs grow adherently but are oval in shape, M1 TAMs are irregular in shape with multiple radial antennae, and M2 TAMs are fusiform with elongated antennae on both sides. These morphological features were used to observe macrophages after treatment with conditioned media.

### Plasmid construction and cell transfection

The LINC00330 overexpression plasmid (pCMV6-LINC00330) was designed and synthesized by Miaoling Bio. The CCL2 overexpression plasmid (PHBLV-CCL2) was purchased from Longqian Biotech (Shanghai, China). Using the Millipore Sigma online website, three sequences of targeted human LINC00330 and CCL2 were screened, and shRNA primers were designed and constructed, as shown in Additional file 2A. The target sequence was used to construct LINC00330 knockdown plasmid (pLKO.1-sh-LINC00330, sh-LINC00330) and CCL2 knockdown plasmid (pLKO.1-sh-CCL2, sh-CCL2). The plasmids were transfected into the ESCC cell lines Kys450, EC109, THP-1, and 293T using Lipofectamine2000 Transfection Reagent (Invitrogen) or PEI transfection reagent (#23966-1, Polysciences). For transient transfection, the effects of gene overexpression or silencing were detected at the mRNA and protein levels after 48 h. For lentiviral packaging and infection, the supernatant was collected after 48 h of transfection, and the virus was concentrated and collected using lentiviral concentration kits (#BW-V2001, BIOMIGA). Then, an appropriate dose of virus and 5 μg/mL of the proviral infection reagent polybrene (#20207023, HANBIO) were added to the target cells for viral infection. After 48 h of infection, 1 μg/mL puromycin (# HB-PU-500, HANBIO) was used for 1 week to screen the stable overexpression and knockdown cell lines.

### Cell proliferation and invasion

A CCK8 assay (CCK8, #B34304, Bimake) was used to test the proliferation of ESCC cells according to the manufacturer’s instructions. Briefly, 10^3^ cells/well were seeded in 100 µL of medium, seeded in 96-well plates, and incubated for 0 h, 24 h, 48 h, 72 h, or 96 h in a humidified incubator containing 5% CO_2_. CCK8 solution (10 µL) was added to each well. The plate was incubated for 1.5 h, and the absorbance at 450 nm was measured using a Multiskan MK3 microplate reader (Thermo Fisher Scientific).

Transwell assay were used to test the invasion of ESCC cells according to the manufacturer’s instructions. Briefly, approximately 4 × 10^4^ ESCC cells were suspended in 300 µL of serum-free RPIM-1640 and seeded in the upper chamber of a 24-well plate, and 600 µL of RPIM-1640 supplemented with 20% FBS (or conditioned medium resulting from special treatment) was carefully added to the lower chamber. The cells were incubated at 37 °C for 28 h. The nonmigratory cells in the upper layer were removed, and the migratory cells were fixed with 4% paraformaldehyde (#SLI830, Coolaber) at room temperature for 15 min, followed by staining with 0.1% crystal violet solution (#G1062, Solarbio). Images were captured using a light microscope (Leica DMI8) and quantified by counting the number of cells in five randomly selected fields of view in each well.

### RNA extraction and real-time polymerase chain reaction (PCR)

Total RNA was isolated from tissues and cell lines using TRIzol reagent (#R401-01, Vazyme) according to the manufacturer’s protocol. RNA purity was determined, and quantification was performed using a NanoDrop 2000 spectrometer (#Nano-100, Allsheng). Reverse transcription was performed using HiSCRIPTR III 1st Strand cDNA Synthesis Kit (#R312-02, Vazyme) and HiSCRIPTR II Q RT SuperMix (#R223-01, Vazyme) to obtain lncRNA and mRNA cDNA, respectively. Real-time PCR (RT–PCR) was performed using the HiSCRIPTR Universal SYBR qPCR Master Mix (#Q511-02, Vazyme) and a Pikorea196 Real-Time PCR Detection System (Thermo Fisher Scientific). GAPDH was used as an endogenous normalization control, and relative expression levels were calculated using the 2^−ΔΔCt^ method. All experiments were performed in triplicate. Information about the primers used is shown in Additional file 2B.

### Western blot analysis

Cell lysis buffer (#R0020, Solarbio) was used to isolate proteins from cells. A BCA kit (#PC0020, Solarbio) was used to determine the protein concentration. Protein loading buffer (#WB2001, New Saimei Biotechnology, Inc.) was used to normalize the protein concentration. After 12% sodium dodecyl sulfate polyacrylamide gel electrophoresis, the proteins were transferred onto PVDF membranes (#IPVH00010, Millipore). The cells were blocked for 1 h at room temperature with blocking solution (#P0252, Beyotime). Primary antibodies against E-cadherin (#3195, CST), Snail (#ab180714, Abcam), Vimentin (#ab92547, Abcam), N-cadherin (#ab76011, Abcam), CCL2 (#ab214819, Abcam), CCR2 (#ET1611-65, HUABIO), p-ERK1/2 (#4390S, CST), p-AKT (#4060T, CST), P-STAT3 (#9139s, CST), p-mTORC1(#2971, CST), and GAPDH (#F033205, Abways) were added and incubated overnight at 4 °C. The sections were then incubated with horseradish peroxidase (HRP)-conjugated secondary antibodies (goat anti-rabbit or goat anti-mouse, 1/10,000) for 2 h at room temperature. The target proteins were visualized using an enhanced chemiluminescence kit (#KE0101, Kemix) and developer (US GE Amersham Imager 680), and the density of the target proteins was analyzed using ImageJ software.

### Flow cytometry

The cells were collected 48 h post treatment, washed, and resuspended in phosphate buffered saline (PBS). For macrophage polarization analysis, samples were stained with PE and APC-conjugated antibodies for cell surface markers. The M1-labeled human antibody APC-CD86 (#APC-65165) and the M2-labeled human antibody PE-CD163 (#PE-65169) were purchased from Proteintech. Unstained and homotype-treated cells were used as controls. Cells treated with isotype controls and unstained cells served as controls. For apoptosis assays, single-cell suspensions were stained with FITC/PI using an Apoptosis Analysis Kit (#E-CK-A211, Elabscience) for 15 min at room temperature. All samples were analyzed on a BD FACS CaliburTM Flow Cytometer (#342975, BD Biosciences) and the data were processed using the FlowJo software program (Tree Star, Ashland, OR).

### Subcellular localization assay

The cytoplasmic and nuclear fractions of ESCC cells were separated using NE-PER™ Nucleus and Cytoplasmic Extraction Reagent (Thermo Scientific™, #78833) according to the manufacturer’s instructions. Briefly, 5 × 10^6^ cells were collected in a centrifuge tube and subjected to two washes with ice-cold PBS. Then, ice-cold cytoplasmic extraction reagent I (CER I) and CER II (from the kit) were added sequentially to the cell pellet. The tube was incubated on ice for 10 min. After that, centrifugation was carried out at maximum speed in a microcentrifuge (~ 16,000×*g*) at 4 °C for 5 min, and the supernatant (cytoplasmic extract) was transferred to a clean prechilled tube. Subsequently, the insoluble (pellet) fraction was suspended in ice-cold NER (from the kit). The tube was incubated on ice for 40 min and vortexed for 15 s every 10 min. Finally, the tube was centrifuged at maximum speed (~ 16,000*g*) for 10 min, and the nuclear extract was harvested (supernatant). Cytoplasmic and nuclear RNA was extracted using TRIzol reagent, and the subcellular localization of LINC00330 and CCL2 was detected by RT‒PCR. Glyceraldehyde 3-phosphate dehydrogenase (GAPDH) and U6 served as the controls for the cytoplasmic and nuclear fractions, respectively.

### LINC00330 RNA pulldown, silver staining, and western blotting (WB)

The sense and antisense sequences of LINC00330 were obtained by PCR amplification (PCR Amplification Kit, #R011, Takara) and in vitro transcription (T7 RNA Polymerase, #10881775001, Roche). The sequences were treated with RNase-free DNase I and purified with the GeneJET RNA Purification Kit (#K0731, Thermo Scientific). Next, LINC00330 sense and antisense sequences were biotinylated using a Biotinylation Kit for Pierce RNA 3' End (#20163, Thermo Scientific™). Then, LINC00330 pulldown experiments were performed using the Pierce™ Magnetic RNA‒Protein Pull-Down Kit (#20164, Thermo Scientific™) according to the manufacturer’s instructions. Briefly, RNA probes (1 μg of biotinylated RNA used per experiment) were combined with streptavidin-coated magnetic beads to form an RNA-bead complex, which was then incubated with EC109 cell lysate. The RNA‒protein complexes were isolated from the other components in the lysate using a magnetic stand. After elution with sodium dodecyl sulfate polyacrylamide gel electrophoresis (SDS‒PAGE) sample buffer, the proteins were separated on a polyacrylamide gel (PAGE). Silver staining of the proteins in the SDS‒PAGE gel was performed using the Pierce™ Mass Spectrometry-compatible Silver Stain Kit (#24600, Thermo Scientific™) according to the manufacturer’s instructions. Alternatively, standard immunoblotting was performed for visualization using a CCL2 antibody (#ab214819, Abcam).

### RNA immunoprecipitation (RIP) assay

The RNA immunoprecipitation (RIP) assay was conducted using Magna RIP kits (#17-700, Millipore, Billerica, MA, USA). Cell lysates were prepared with RIP lysis buffer, and 100 μL of the resulting whole-cell extract was incubated with magnetic beads conjugated to a primary antibody against CCL2 (#ab214819, Abcam). Following incubation, the immunoprecipitated proteins were treated with proteinase K (#70663, Sigma-Aldrich) to release the bound RNA. The RNA was then purified and subjected to RT‒PCR analysis.

### Transcriptome sequencing and data analysis

Kyse450 cells were transiently transfected with a LINC00330 overexpression plasmid. After 48 h, the cells were harvested and total RNA was isolated using TRIzol reagent (Invitrogen, MA, USA). The RNA concentration and purity were assessed using a NanoDrop 2000 spectrophotometer (Thermo Scientific, USA). The RNA samples were then sent to OE Biotech (Shanghai, China) for library construction, sequencing, and subsequent bioinformatics analysis on an Illumina HiSeq X Ten platform. Initial raw data in fastq format were processed with Trimmomatic to remove low-quality reads, yielding clean reads that were mapped to the human genome (GRCh38) using HISAT2. The FPKM for each gene was calculated with Cufflinks, and gene read counts were obtained via HTSeq-count. Differential expression analysis was conducted with the DESeq R package, setting a significance threshold of *P* < 0.05 and a fold change > 2 or < 0.5. Hierarchical clustering analysis illustrated the expression patterns of DEGs across various samples. Enrichment analyses for Gene Ontology (GO) terms, Reactome, and Kyoto Encyclopedia of Genes and Genomes (KEGG) pathways of DEGs were performed using R on the basis of the hypergeometric distribution.

### Bioinformatics analysis

ESCC gene expression data were downloaded from the TCGA (https://www.cancer.gov/ccg/research/genome-sequencing/tcga) and GEO (https://www.ncbi.nlm.nih.gov/geo/) datasets. The GSEA immunological feature gene set (C7) was downloaded from ImmLnc (http://bio-bigdata.hrbmu.edu.cn/ImmLnc/), and immune correlation analysis of lncRNAs was performed using ImmLnc. The gene expression profile and correlation analysis of LINC00330/CCL2/CCR2 and their correlation with prognosis in patients with ESCC were predicted with the help of the UALCAN database (https://ualcan.path.uab.edu/cgi-bin/ualcan-res.pl) and GEPIA database (http://www.gepia.cancer-pku.cn/).

### Xenograft experiments

All animal research was conducted in accordance with the Basel Declaration and approved by the Animal Care and Ethics Committee of Xinxiang Medical College (No. XYLL-2021043). BALB/c mice (6–8 weeks old) were obtained from Beijing Vital River Laboratory Animal Technology Co., Ltd. The animals were randomly divided into four groups, and KYSE450 cells transfected with pCMV6, LINC00330, pLKO.1, and sh-LINC00330 were subcutaneously injected. The concentration of planted cells was 5 × 10^6^ cells in 0.1 mL of normal saline. The animals were monitored for 4–6 weeks after injection. The length (*L*) and width (*W*) of the tumors were measured every 3 days after injection of the cells. The tumor volume (*V*) was calculated using the following formula: *V* = *L* × *W*^2^ × 0.5. After approximately 4–6 weeks of treatment, the animals were sacrificed via cervical dislocation, and the tumors were collected and weighed.

### Statistical analysis

All experiments were independently repeated at least three times. The data are presented as the mean ± standard deviation (SD) unless otherwise stated. Two-tailed Student’s *t* tests were used to compare two groups, while one-way or two-way analysis of variance (ANOVA) was used for comparisons among multiple groups. Correlations were assessed using Pearson’s correlation coefficient. Survival curves were generated using the Kaplan‒Meier method and analyzed using the log-rank test for univariate assessments, with a significance level set at *P* < 0.05. All the statistical analyses were conducted using Graph Pad Prism 9. A *P* value less than 0.05 was considered to indicate statistical significance, denoted as *P < 0.05, **P < 0.01, or ***P < 0.001.

## Results

### LINC00330 as a potential new therapeutic target and biomarker for ESCC

**Fig. 1:**
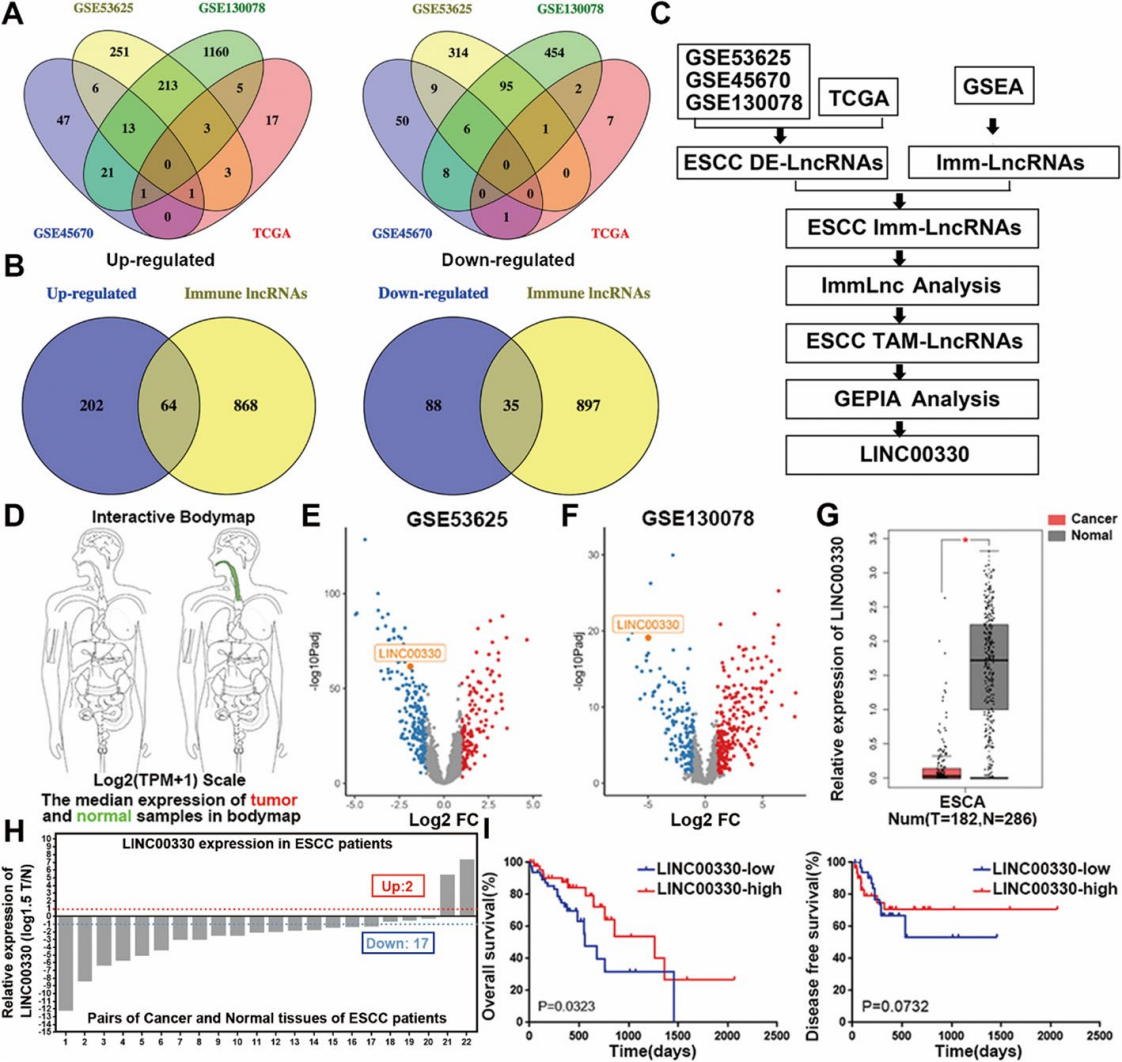
LINC00330 is expected to become a new target and biomarker for the treatment of ESCC. **A** LncRNAs with significantly upregulated and downregulated expression in the GSE53625, GSE130078, GSE45670, and TCGA datasets. **B** Intersection of immune-related lncRNAs with significantly upregulated or downregulated expression in the GSE53625, GSE130078, GSE45670, and TCGA datasets.** C** Screening flow chart of the ESCC TAM-associated lncRNA LINC00330. **D** The expression level of LINC00330 in different parts of the human body. Red represents tumor tissue and green represents normal tissue. **E**, **F** LINC00330 was significantly expressed at low levels in the GSE53625 and GSE130078 datasets.** G** The expression of LINC00330 in esophageal carcinoma tissues and paired paracancerous tissues in the TCGA database. **H** RT‒PCR detection of LINC00330 expression in 22 pairs of ESCC clinical samples.** I** Kaplan‒Meier analysis of prognostic survival in patients with high or low expression of LINC00330 in ESCC. The results are presented as the mean ± SD, * *P* < 0.05, ** *P* < 0.01, *** *P* < 0.001

To screen and identify TAM-associated lncRNAs in ESCC, transcriptional sequencing data of cancerous and adjacent normal esophageal tissue from 312 patients with ESCC were collected from the GEO (GSE53625, GSE130078, and GSE45670) and TCGA databases as of 1 November 2018. Differential expression analysis across datasets yielded 3102 lncRNAs, with 1077 downregulated and 2025 upregulated (Fig. 1A & Additional file 1: P1–4). Then, lncRNAs significantly differentially expressed in at least two databases (266 upregulated and 123 downregulated, Additional file 1: P5–6) were intersected with 932 immune-related lncRNAs (Additional file 1: P7) from the immune-related GSEA molecular signature database, resulting in 99 ESCC immune-related lncRNAs (64 upregulated lncRNAs and 35 downregulated lncRNAs; Fig. 1B & Additional file 1: P8–9). Next, using the ImmLnc web tool, we analyzed the correlation between these 99 lncRNAs and immune cell infiltration (mainly CD8^+^ T cells, dendritic cells, neutrophils, CD4^+^ T cells, B cells, and macrophages) in the ESCC TME (Additional file 1: P10–11), and 11 lncRNAs associated with TAM infiltration were identified (Additional file 1: P12). Among them, six lncRNAs (two upregulated, NR2F1-AS1 and ZFHX4-AS1; four downregulated, HAND2-AS1, LINC00330, RP11-834C11.4, and TTTY10) were defined as ESCC TAM-associated lncRNAs, which were only associated with TAM infiltration and had no correlation with infiltration of other immune cells (|*r*|> 0.3, *P* < 0.05; Additional file 3: Table S1). Finally, a series of literature investigations, bioinformatics reanalysis, and preexperimental detection were carried out, and LINC00330 was ultimately selected as the focus of this project.

Subsequently, the expression profile and clinical relevance of LINC00330 in ESCC were evaluated. Analysis of sequencing data from ESCC tissue samples in the TCGA and GEO databases indicated that LINC00330 was highly expressed in normal esophageal epithelial tissue (Fig. 1D) and significantly downregulated in ESCC tissue (Fig. 1E–G). Similarly, LINC00330 expression was assessed in the cancerous and adjacent normal tissues of 22 patients with ESCC who were pathologically diagnosed and underwent tumor resection surgery. The results demonstrated that LINC00330 was consistently underexpressed in the majority of ESCC tissues (17/22) compared with adjacent normal tissues (Fig. 1H). Moreover, clinical data from the TCGA were used to evaluate the associations between LINC00330 levels and clinical parameters in patients with ESCC. Kaplan–Meier analysis revealed a close association between low LINC00330 expression and poor prognosis in patients with ESCC. Patients with low LINC00330 expression had significantly worse overall survival and disease-free survival (Fig1I). However, no significant correlation was observed with other clinical parameters, such as age, sex, TNM stage, or tumor grade (Additional file 3: Table S2). In summary, our study indicated that the downregulation of LINC00330 is significant in ESCC and that its expression can predict patient outcomes.

### Overexpression of LINC00330 inhibits ESCC progression in vivo and in vitro

To further clarify the function of LINC00330, we first analyzed the relative expression levels of LINC00330 in the normal esophageal epithelial cell line SHEE and five ESCC cell lines (Additional file 3: Figure S1A). We selected the Kyse450 and EC109 cell lines for subsequent tests owing to their moderate levels of LINC00330 expression. Next, we constructed LINC00330 overexpression (LINC00330) and knockdown (sh-LINC00330) plasmids. Overexpression (Additional file 3: Figure S1B) and knockdown (Additional file 3: Figure S1C) of LINC00330 in ESCC cell lines were achieved by transient transfection or lentiviral packaging and infection, followed by a series of gain-of-function and loss-of-function experiments.

**Fig. 2:**
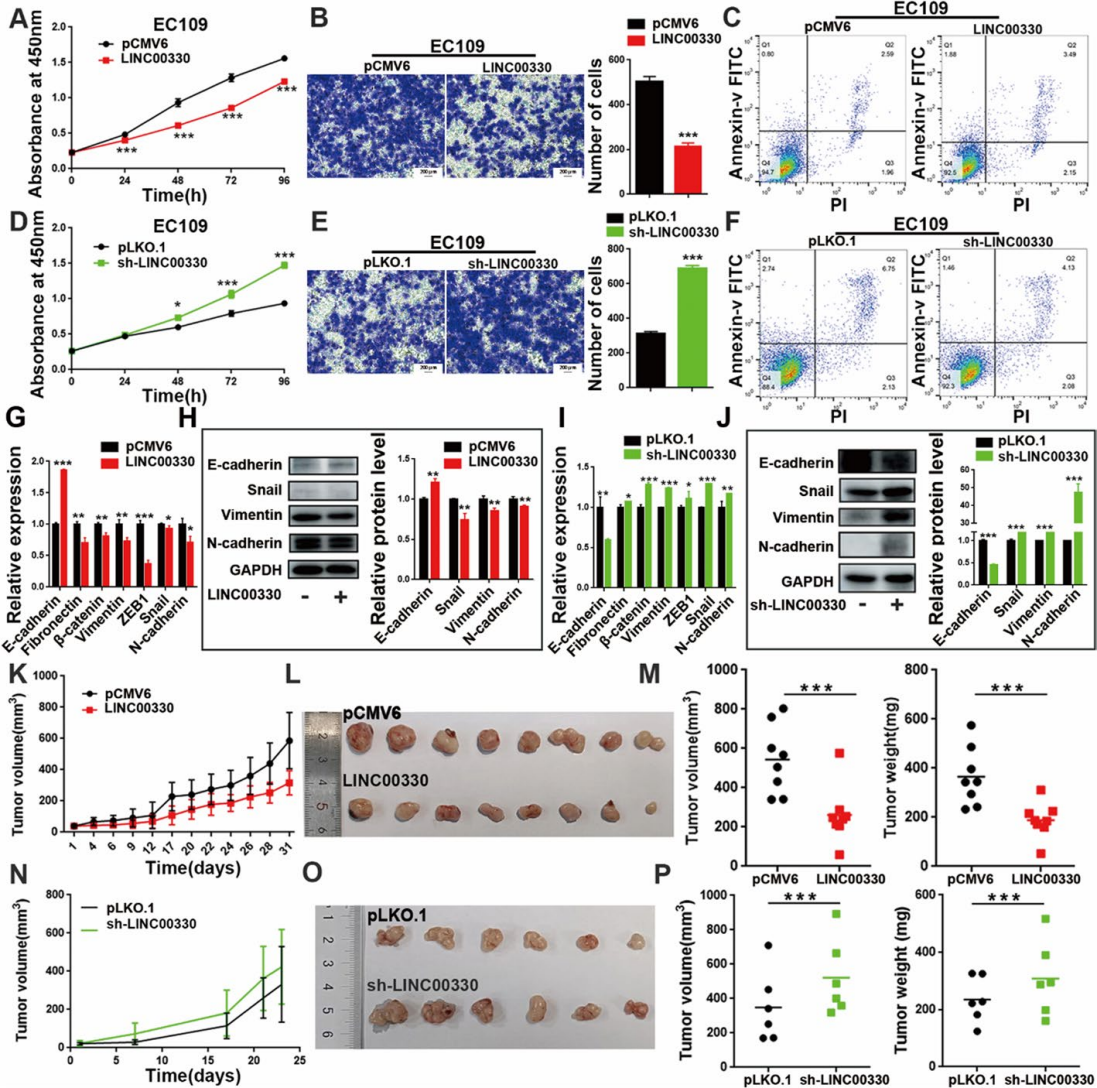
LINC00330 inhibits ESCC progression in vivo and in vitro. **A**, **D** CCK-8 assays were used to detect the effect of LINC00330 overexpression or knockdown on the proliferation of EC109 cells. **B**, **E** Transwell assays were used to detect the effect of LINC00330 overexpression or knockdown on the invasion of EC109 cells. Scale bar = 200 μm. **C**, **F** Flow cytometry was used to detect the effect of LINC00330 overexpression or knockdown on apoptosis in EC109 cells. **G**‒**J** RT‒PCR and WB were used to detect the effect of LINC00330 overexpression or knockdown on the EMT of EC109 cells. **K**‒**M** Overexpression of LINC00330 inhibited tumor growth in mice, and the tumors produced at the same time became increasingly smaller. **N**–**P** Knockdown of LINC00330 promoted subcutaneous tumor formation in nude mice, resulting in increased tumor size and increased tumor weight. The figure shows the mean ± SD of the statistical results from three independent experiments; * *P* < 0.05, ** *P* < 0.01, *** *P* < 0.001

The results showed that overexpression of LINC00330 significantly inhibited the proliferation (Fig. 2A, and Additional file 3: Figure S1D) and invasion (Fig. 2B, and Additional file 3: Figure S1E) of ESCC cells and promoted their apoptosis (Fig. 2C, and Additional file 3: Figure S1F). Conversely, knockdown of LINC00330 promoted the proliferation (Fig. 2D, and Additional file 3: Figure S1G) and invasion (Fig. 2E, and Additional file 3: Figure S1H) of ESCC cells and inhibited apoptosis (Fig. 2F and Additional file 3: Figure S1I).

Additionally, when culturing ESCC cells with LINC00330 knocked down, we unexpectedly observed that these cells acquired mesenchymal-like morphological characteristics (morphology changed from polygonal or pebble-like to elongated spindle or spindle-shaped), whereas ESCC cells overexpressing LINC00330 maintained the morphological characteristics of epithelial cells better (the cell morphology was mostly polygonal or pebble-like) (Additional file 3: Figure S1J&K). Therefore, we further assessed the changes in epithelial–mesenchymal transition (EMT) markers in ESCC cells by RT‒PCR and WB. The results indicated that upregulation of LINC00330 expression in ESCC cells promoted the expression of the epithelial marker E-cadherin and reduced the expression of mesenchymal markers (Fibronectin, β-catenin, Vimentin, ZEB1, Snail, and N-cadherin; Fig. 2G&H, and Additional file 3: Figure S1L). Conversely, in cells with downregulated LINC00330, the epithelial marker E-cadherin was decreased, while mesenchymal markers were upregulated to varying degrees (Fig. 2I, J, and Additional file 3: Figure S1M).

We also evaluated the function of LINC00330 in vivo using a subcutaneous xenograft model in nude mice. Subcutaneous injection of LINC00330-overexpressing ESCC cells suppressed tumor growth, resulting in decreased tumor volumes and decreased body weights (Fig. 2K–M). In contrast, mice injected with LINC00330-knockdown ESCC cells displayed enhanced tumor formation capacity, with larger tumor volumes and heavier body weights than those of the control group (Fig. 2N–P).

In conclusion, the study highlights the role of LINC00330 as a key inhibitor in the development of ESCC and demonstrates its ability to inhibit tumor progression both in vitro and in vivo, suggesting its potential as a therapeutic target and biomarker for ESCC treatment.

### LINC00330 plays a significant role in the reprogramming of TAMs in ESCC

**Fig. 3:**
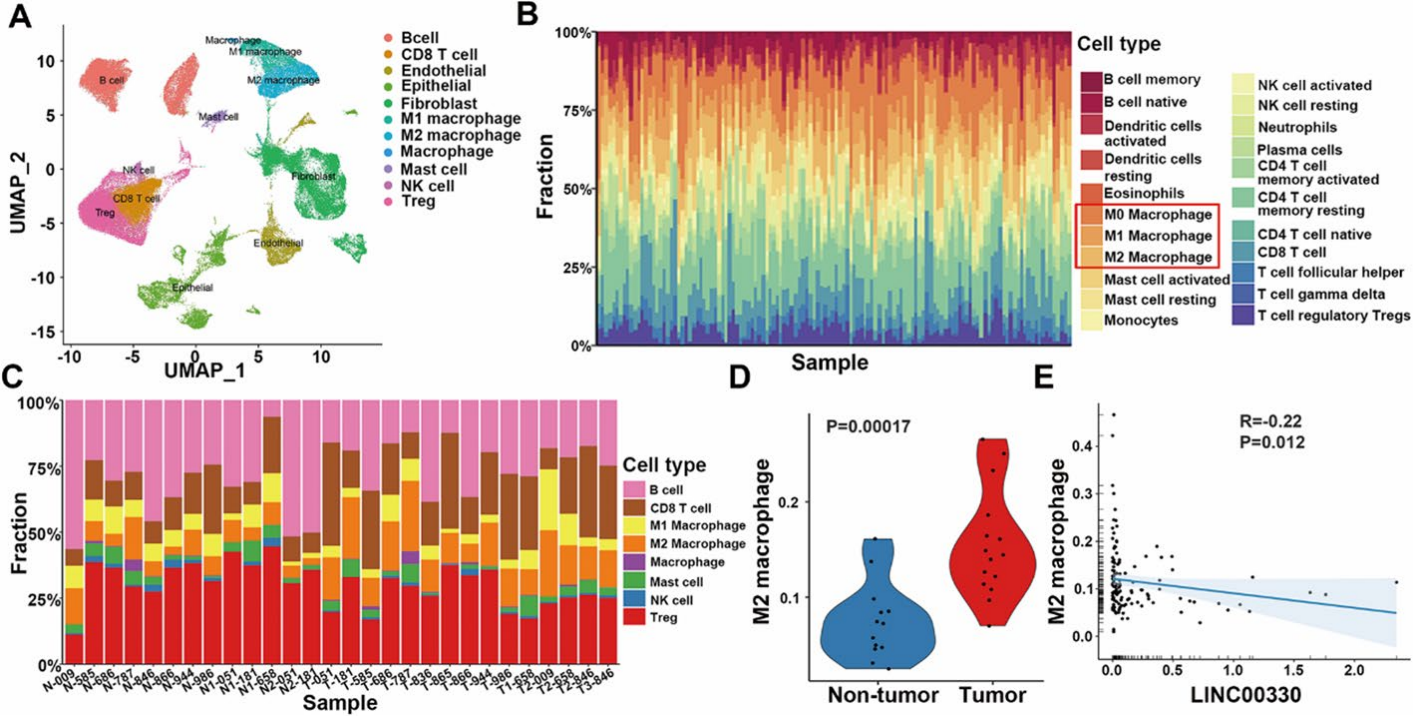
LINC00330 plays an important role in ESCC TAM reprogramming. **A** Cluster analysis and type annotation of esophageal cancer tissue cells from single-cell high-throughput sequencing data (PRJNA777911). **B** Evaluation of immune infiltration of 22 immune cell types in ESCA patient tumor tissues in the TCGA cohort using CIBERSORTx. **C** Histogram showing the proportion of immune cells in tumor tissue for each patient. **D** The proportion of M2 macrophages in tumor tissues was significantly greater than that in nontumor tissues (Student’s *t* test, *P* = 0.00017). **E** CIBERSORTx analysis showed that LINC00330 expression was negatively correlated with M2 macrophage infiltration

Next, we evaluated the correlation between LINC00330 and ESCC TAMs. High-throughput single-cell sequencing data from cancerous tissues (14 samples) and adjacent noncancerous tissues (13 samples) of patients with ESCC were collected (PRJNA777911), and a detailed analysis of the cellular composition within the ESCC tumor microenvironment (TME) was conducted. The results demonstrated a rich immune cell population in the ESCC TME, particularly groups of B cells, T cells, and macrophages (Fig. 3A), consistent with previous reports [12, 25]. Additionally, using CIBERSORTx (https://cibersortx.stanford.edu/), we quantified 22 types of immune cell infiltrates from RNA sequencing data of ESCC in The Cancer Genome Atlas (TCGA) database. Similar to the existing findings, the four predominant types of infiltrating immune cells in the ESCC TME were CD4 memory resting T cells, M0 macrophages, CD8 T cells, and M2 macrophages (Fig. 3B). Further analysis revealed that TAM infiltration was significantly higher in tumor tissues than in adjacent noncancerous tissues, with a predominance of M2 TAMs in ESCC tissues (Fig. 3C, D). The expression of LINC00330 was negatively correlated with the infiltration of M2 TAMs (Fig. 3E).

In addition, using the GEPIA database (http://gepia.cancer-pku.cn/), we evaluated the correlation between the expression of LINC00330 and specific markers of these immune cells within the ESCC immune microenvironment. There was no significant correlation between the expression of LINC00330 and the expression of CD4 memory resting T cells, such as CCR7 (*R* = −0.059, *P* = 0.43), CD8 T cells, CD8 (*R* = −0.075, *P* = 0.31), or CD27 (*R* = −0.07, *P* = 0.35). However, there were significant negative correlations with the panmacrophage markers CD106 (*R* = −0.33, *P* = 6.50 × 10^−9^) and CD81 (*R* = −0.47, *P* = 6.20 × 10^−40^). Notably, LINC00330 expression was significantly positively correlated with markers for M1 macrophages, such as IL-12α (*R* = 0.56, *P* = 0) and IRF5 (*R* = 0.48, *P* = 0), and negatively correlated with several M2 macrophage markers, including CD209 (*R* = −0.27, *P* = 8.50 × 10^−16^), CD163 (*R* = −0.24, *P* = 2.10 × 10^−10^), CD206 (*R* = −0.27, *P* = 1.60 × 10^−13^), and IL-10 (*R* = −0.2, *P* = 7.90 × 10^−08^) (Additional file 3: Table S3).

We also utilized RT–PCR to analyze the relationship between LINC00330 expression and the infiltration of different types of TAMs in ESCC clinical samples and mouse tumor tissues (collected from Figs. 2L and O). The analysis of ESCC clinical samples indicated that in the majority of tumor tissues with low expression of LINC00330, there was a significant increase in CD68^+^ TAM infiltration (Additional file 3: Figure S2A). Notably, the interstitial infiltration of CD80^+^ M1 macrophages was significantly reduced (Additional file 3: Figure S2B), while the accumulation of CD163^+^ M2 macrophages was more pronounced (Additional file 3: Figure S2C). Similarly, in mouse tumor tissues, the expression of the panmacrophage marker CD68 was lower in the LINC00330 group than in the control group (Additional file 3: Figure S2D) but was significantly greater in the sh-LINC00330 group (Additional file 3: Figure S2E). Furthermore, compared with those in the control group, the expression of the M1 markers CD80 and CD86 was significantly increased in the tumor tissues of the LINC00330 group (Additional file 3: Figure S2F, G), while M2 TAM infiltration was reduced (as indicated by the significant reduction in the expression of the M2 surface markers CD163 and CD206) (Additional file 3: Figure S2H, I). Conversely, compared with that in the control group, M1 TAM infiltration was reduced in the sh-LINC00330 group (Additional file 3: Figure S2J, K), while M2 TAM infiltration was significantly increased (Additional file 3: Figure S2L, M).

In summary, our study suggested that LINC00330 plays a significant role in the reprogramming of TAMs in ESCC. Increasing the expression of LINC00330 may improve the state of the ESCC TME, promoting the polarization of TAMs (especially M2 TAMs) toward the M1 phenotype.

### LINC00330-mediated TAM reprogramming inhibits ESCC proliferation and migration

**Fig. 4:**
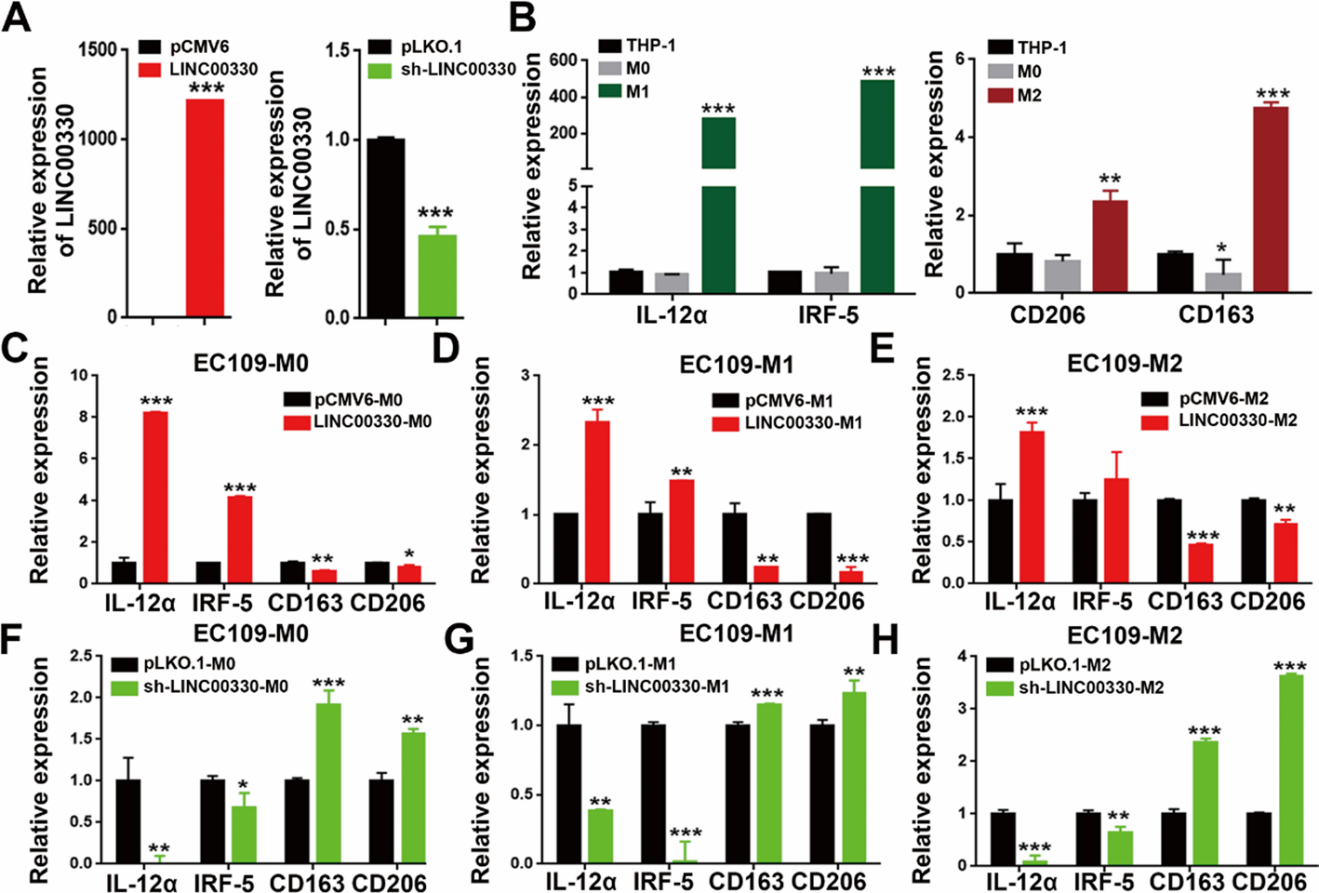
LINC00330 facilitates ESCC TAM reprogramming.** A** Overexpression or knockdown of LINC00330 in EC109 cells.** B** RT‒PCR was used to detect the expression of markers of M1 (IL-12α and IRF-5) and M2 (CD163 and CD206) macrophages. **C**, **F** M0 macrophages were cocultured with conditioned medium generated by the overexpression or knockdown of LINC00330 in EC109 cells, and the expression of M1 and M2 markers was detected by RT‒PCR. **D**, **G** M1 macrophages were cocultured with conditioned medium generated by the overexpression or knockdown of LINC00330 in EC109 cells, and the expression of M1 and M2 markers was detected by RT‒PCR. **E**, **H** M2 macrophages were cocultured with conditioned medium generated by the overexpression or knockdown of LINC00330 in EC109 cells, and the expression of M1 and M2 markers was detected by RT‒PCR. The data are presented as means ± SDs; * *P* < 0.05, ** *P* < 0.01, *** *P* < 0.001

To further clarify the role of LINC00330 in ESCC TAM reprogramming, we conducted “ESCC-macrophage coculture experiments.” Initially, we overexpressed or knocked down LINC00330 in ESCC cells (Fig. 4A, and Additional file 3: Figure S3A&E) and collected the conditioned medium. Subsequently, human mononuclear THP-1 cells were induced with PMA to differentiate into macrophages; IFN-γ and IL-4 were used to induce macrophage polarization into M1 and M2 TAMs, respectively. The success of the induction was confirmed by RT‒PCR detection of M1 TAM markers (IL-12α and IRF5) and M2 TAM markers (CD163 and CD206; Fig. 4B). Then, conditioned media from ESCC cells under different conditions were cocultured with various types of macrophages (M0, M1, M2) to assess the impact of LINC00330 on TAM reprogramming. The results demonstrated that conditioned media from ESCC cells overexpressing LINC00330 promoted polarization toward M1 TAMs, as characterized by increased expression of IL-12α and IRF5 and decreased expression of CD163 and CD206 (Fig. 4C–E, and Additional file 3: Figure S3B-D). Conversely, conditioned media from ESCC cells with LINC00330 knockdown promoted polarization toward M2 TAMs (Fig. 4F–H and Additional file 3: Figure S3F- H).

**Fig. 5:**
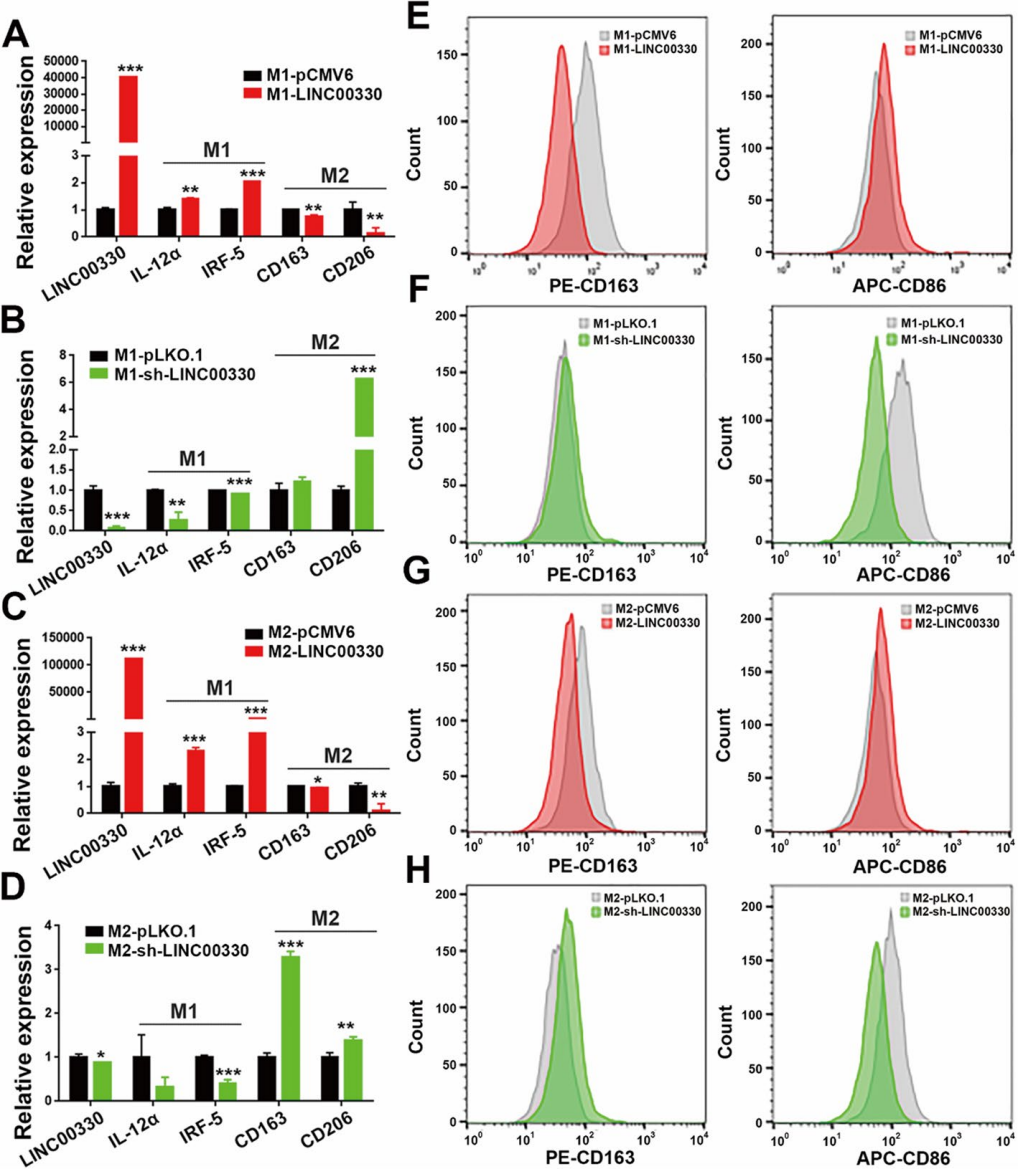
LINC00330 can mediate reprogramming of different types of TAMs. **A**, **E** Overexpression of LINC00330 in M1 macrophages. RT‒PCR and flow cytometry were used to detect the expression of LINC00330 and M1/M2 markers. **B**, **F** After LINC00330 was knocked down in M1 macrophages, RT‒PCR and flow cytometry were used to detect the expression of the LINC00330 and M1/M2 markers. **C**, **G** Overexpression of LINC00330 in M2 macrophages. RT‒PCR and flow cytometry were used to detect the expression of the LINC00330 and M1/M2 markers. **D**, **H** After LINC00330 knockdown in M2 macrophages, RT‒PCR and flow cytometry were used to detect the expression of LINC00330 and M1/M2 markers. The figure shows the mean ± SD of three independent experiments, * *P* < 0.05, ** *P* < 0.01, *** *P* < 0.001

Next, we overexpressed and knocked down LINC00330 in M1 and M2 macrophages to determine whether LINC00330 could endogenously affect macrophage polarization. As shown in Fig. 5A–D, overexpression of LINC00330 enhanced the expression of M1 markers (IL-12α and IRF-5) in both M1 and M2 macrophages while reducing the expression levels of M2 markers (CD163 and CD206). In contrast, knocking down LINC00330 inhibited M1 polarization and enhanced M2 polarization. We also performed similar experiments using flow cytometry and obtained consistent results: there was an increase in the number of CD86^+^ M1 macrophages and a significant decrease in the number of CD163^+^ M2 macrophages after LINC00330 overexpression (Fig. 5E, G). Conversely, after knockdown of LINC00330, the number of CD86^+^ M1 macrophages significantly decreased, while the number of CD163^+^ M2 macrophages increased (Fig. 5F, H). In summary, these data suggest that LINC00330 can promote TAM reprogramming. Upregulation of LINC00330 expression activates M1 polarization and inhibits M2 polarization.

Finally, we evaluated the effects of LINC00330-mediated TAM reprogramming on the progression of ESCC. We overexpressed or knocked out LINC00330 in M1 macrophages and cocultured the cells with ESCC cell lines. As shown in Additional file 3: Figure S4, compared with the control group, the overexpression of LINC00330 in M1 macrophages restricted the growth of ESCC cells (Additional file 3: Figure S4A), promoted apoptosis (Additional file 3: Figure S4B), and inhibited the invasion of ESCC cells (Additional file 3: Figure S4C). In contrast, sh-LINC00330 in M1 cells significantly promoted ESCC cell growth (Additional file 3: Figure S4D), inhibited apoptosis (Additional file 3: Figure S4E), and enhanced tumor cell invasion (Additional file 3: Figure S4F). We performed similar experiments in M2 macrophages and obtained the same results; that is, M1 polarization mediated by LINC00330 overexpression inhibited the proliferation and migration of ESCC cells and promoted apoptosis (Additional file 3: Figure S4G–I). However, knockdown of LINC00330 led to suppressed M1 polarization, activated M2 polarization, and consequently promoted the proliferation and invasion of ESCC cells while inhibiting apoptosis in ESCC cells (Additional file 3: Figure S4J–L).

In summary, our study demonstrated that LINC00330 plays a significant role in TAM reprogramming and that LINC00330-mediated TAM reprogramming can inhibit the progression of ESCC.

### LINC00330 inhibits the expression of CCL2 protein and the activation of its downstream signaling pathway

**Fig. 6:**
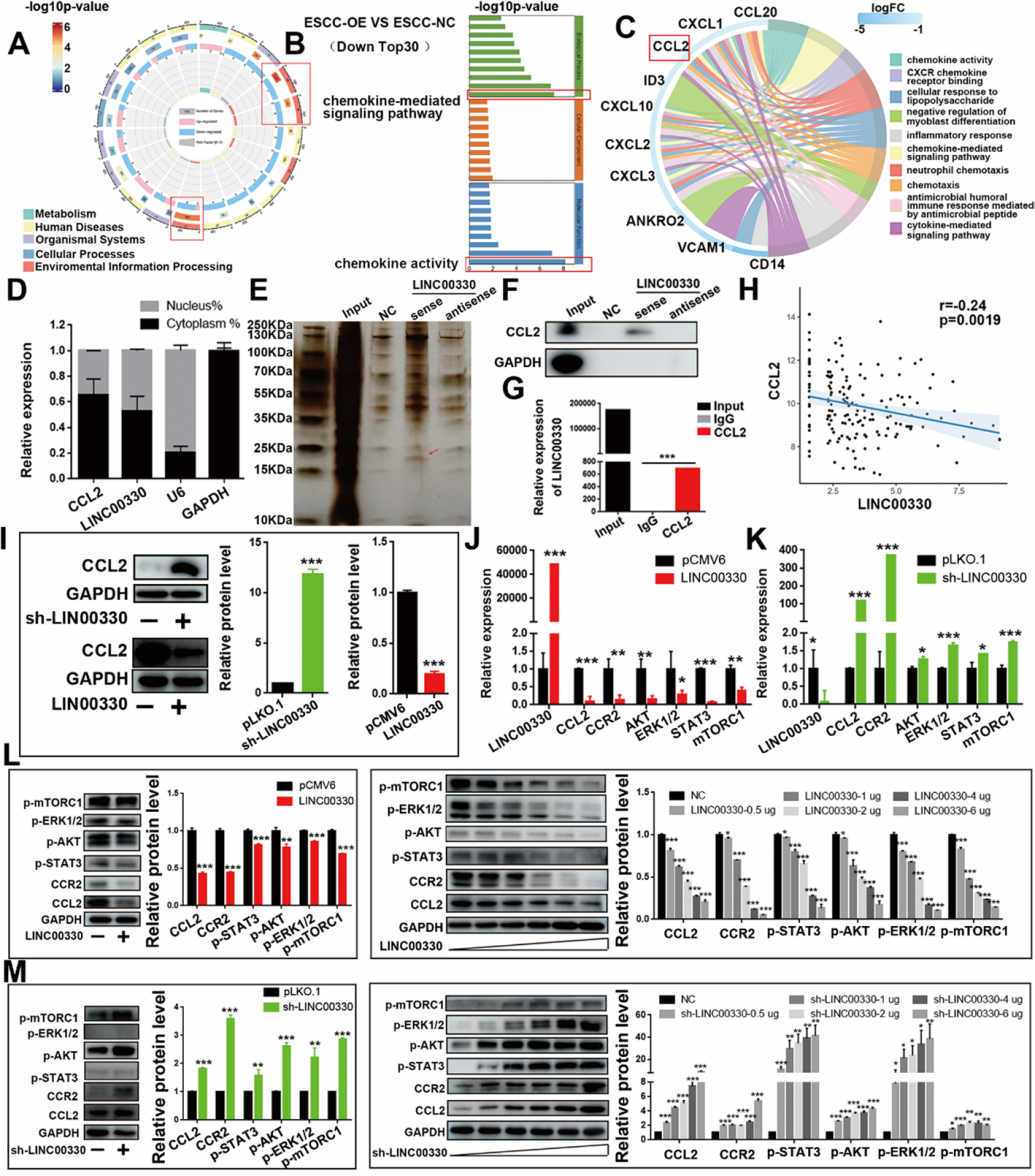
LINC00330 can inhibit the expression of CCL2 and the activation of its downstream signaling pathway. **A** KEGG analysis of the physiological pathways associated with the main regulation of LINC00330. **B**, **C** The top 30 genes downregulated by LINC00330 were “pathway” enriched according to the Reactome database. **D** The subcellular localization of CCL2 and LINC00330 was determined by RT‒PCR after cytoplasmic nucleus isolation. **E** Silver staining analysis after the LINC00330 RNA pulldown experiment. **F** The interaction between CCL2 and LINC00330 was detected by WB after the RNA pulldown experiment.** G** The interaction between CCL2 and LINC00330 was detected by RT‒PCR after RIP. **H** Pearson correlation analysis was used to analyze the correlation between LINC00330 and CCL2 expression.** I** WB was used to detect the effect of LINC00330 overexpression or knockdown on CCL2 protein expression in EC109 cells. **J**, **K** The effect of LINC00330 overexpression or knockdown in ESCC cells on the CCL2/CCR2 axis and its downstream signaling pathway was detected via RT‒PCR. **L**, **M** The effect of LINC00330 overexpression or knockdown on the CCL2/CCR2 axis and its downstream signaling pathway in ESCC cells was detected via WB. The figure shows the mean ± SD of the statistical results from three independent experiments; * *P* < 0.05, ** *P* < 0.01, *** *P* < 0.001

To elucidate the molecular mechanisms underlying the effects of LINC00330. We collected a control group (NC) and LINC00330-overexpressing Kyse450 cells for transcriptomic sequencing and bioinformatics analysis. KEGG analysis revealed that LINC00330 primarily affected “environment information processing” (Fig. 6A). GO enrichment analysis of the top 30 downregulated genes related to LINC00330 revealed that LINC00330 significantly affected the “chemokine activity” and “chemokine-mediated signaling pathway” in the immune system (Fig. 6B), with CCL2 being the gene with the most overlap (Fig. 6C).

To further elucidate the relationship between LINC00330 and CCL2, we first determined the subcellular localization of these proteins. Nuclear-cytoplasmic fractionation experiments revealed that LINC00330 and CCL2 were colocalized in the cytoplasm of ESCC cells (Fig. 6D). According to previous studies, the subcellular localization of lncRNAs is closely related to their functions. Cytoplasmic localization of lncRNAs often involves signal transduction, posttranscriptional regulation, translation, and posttranslational modifications [16]. Therefore, we hypothesize that LINC00330 exerts diverse functions by forming RNA‒protein complexes with proteins. To further clarify the relationship between LINC00330 and CCL2 proteins, we constructed sense and antisense probes for LINC00330, biotin-labeled them, and performed RNA pulldown experiments, silver staining (Fig. 6E) and CCL2-specific WB (Fig. 6F). The results showed that only the sense probe for LINC00330 immunoprecipitated with the CCL2 protein, while the negative control group and the antisense probe group did not show this interaction (Fig. 6E, F). This indicates that there is direct targeted binding between LINC00330 and the CCL2 protein. In addition, through RIP experiments, we confirmed the interaction between LINC00330 and the CCL2 protein (Fig. 6G). Subsequently, we also analyzed the correlation between LINC00330 and CCL2 through clinical correlation analysis, Pearson correlation analysis, and WB detection. We found that CCL2 was more highly expressed in ESCC tissues (Additional file 3: Figure S5A) and that its high expression was closely correlated with a higher stage grade (Additional file 3: Figure S5B) and poor prognostic survival (Additional file 3: Figure S5C, D), as opposed to LINC00330 (Fig. 1H, I). Pearson correlation analysis revealed that LINC00330 expression was negatively correlated with CCL2 expression (Fig. 6H). In addition, LINC00330 overexpression inhibited CCL2 protein expression, whereas LINC00330 knockdown significantly upregulated CCL2 protein expression (Fig. 6I). These results suggest that CCL2 is a target of LINC00330. LINC00330 can directly bind to the CCL2 protein to form RNA‒protein complexes that inhibit its translation and function.

Additionally, CCL2 preferentially binds to CCR2 receptors and initiates a variety of signal transduction pathways (including more classical M1/M2 activation pathways, such as the PI3K/AKT, ERK, JAK-STAT and NF-κB signaling pathways [26]), stimulating cell migration and mediating tumor pathogenesis [27]. In our study, GSEA and KEGG mapping revealed that LINC00330 can negatively regulate “chemokine-mediated signaling pathways,” such as the “JAK–STAT signaling pathway,” “MAPK signaling pathway,” and “PI3K–AKT signaling pathway” (Additional file 3: Figure S5E, F). Therefore, we examined the effect of changes in LINC00330 expression on the expression of key genes in the CCL2/CCR2 axis and downstream signaling pathways (such as CCL2/CCR2, JAK/STAT3, ERK1/2, and AKT/mTORC1). We first evaluated the correlation between the expression of LINC00330 and CCR2, as well as between CCL2 and CCR2, in ESCC clinical samples. The results showed a weak negative correlation between CCR2 and LINC00330 expression (Additional file 3: Figure S5G) and a positive correlation with CCL2 expression (Additional file 3: Figure S5H). Furthermore, CCR2 was found to be highly expressed in ESCC tumor tissues (Additional file 3: Figure S5I), and its high expression was indicative of poorer survival (Additional file 3: Figure S5J), similar to that of CCL2 (Additional file 3: Figure S5C&D), but in contrast to that of LINC00330 (Fig. 1I). Next, we examined the effect of changes in LINC00330 expression on the expression of key genes in the CCL2/CCR2 axis and downstream signaling pathways (such as CCL2/CCR2, JAK/STAT3, ERK1/2, and AKT/mTORC1). The results showed that overexpression of LINC00330 suppressed the expression of CCL2/CCR2 and inhibited the activation of key genes in signaling pathways. Conversely, knockdown of LINC00330 activated the CCL2/CCR2 axis and related downstream signaling pathways (Fig. 6J–M & Additional file 3: Figure S5K–M).

In summary, our research demonstrated a negative regulatory relationship between LINC00330 and CCL2. LINC00330 binds to the CCL2 protein and inhibits the expression of CCL2 and downstream signaling pathways. This finding suggested that LINC00330 can suppress the progression of ESCC by inhibiting the expression of CCL2, thereby blocking the activation of the CCL2/CCR2 axis and its downstream signal transduction pathways, promoting TAM reprogramming, and inhibiting ESCC progression.

### CCL2 is critical for LINC00330-mediated TAM reprogramming and ESCC progression

**Fig. 7:**
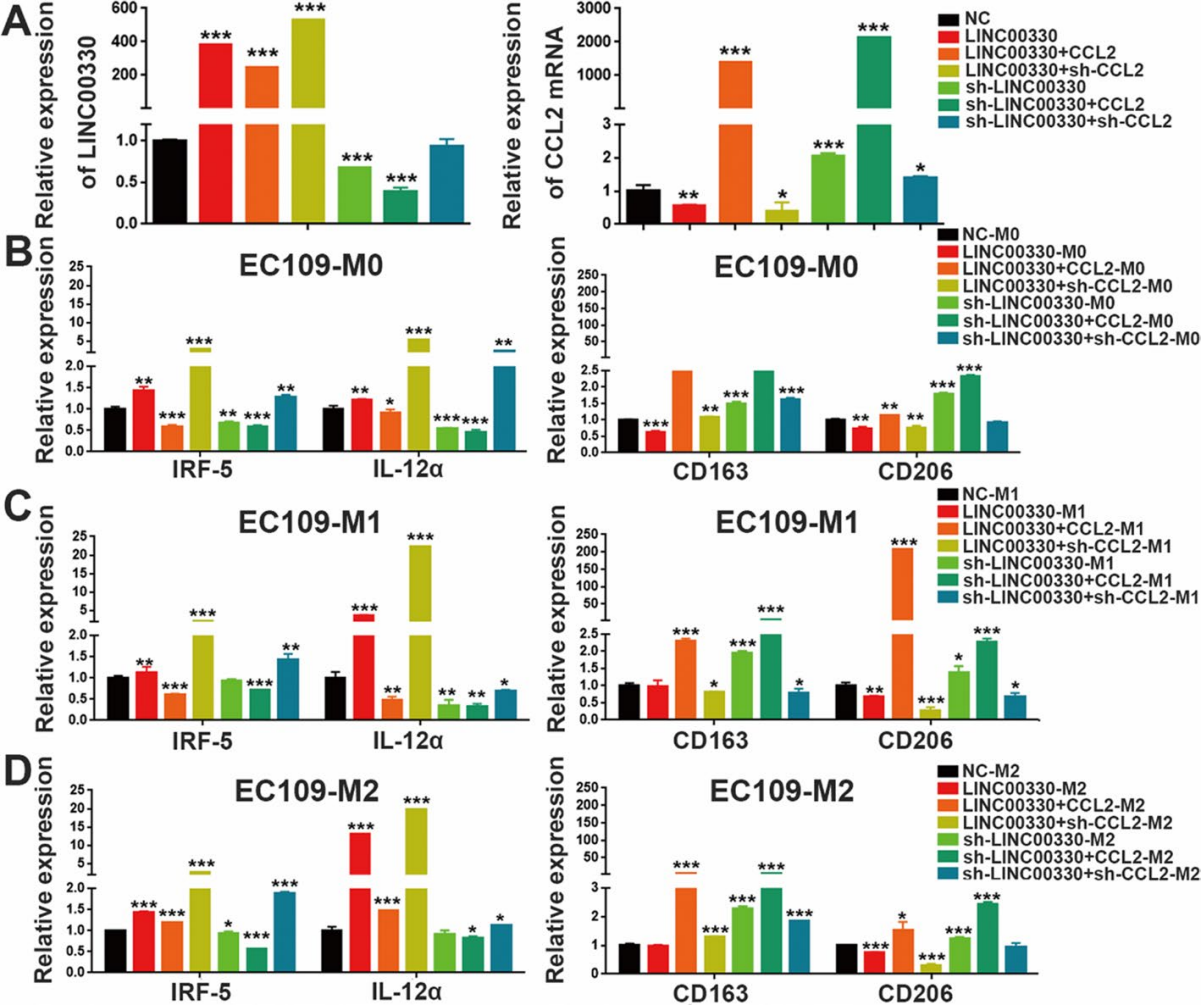
CCL2 plays an important role in LINC00330-mediated TAM reprogramming in ESCC. **A** RT‒PCR was used to detect the expression of LINC00330 and CCL2 in EC109 cells under different treatment conditions. **B** M0 macrophages successfully induced were cocultured with conditioned medium generated by different experimental groups, and the expression of M1 and M2 markers was detected by RT‒PCR. **C** Successfully induced M1 macrophages were cocultured with conditioned medium generated by different experimental groups, and the expression of M1 and M2 markers was detected by RT‒PCR. **D** Successfully induced M2 macrophages were cocultured with conditioned medium generated by different experimental groups, and the expression of M1 and M2 markers was detected by RT‒PCR. The figure shows the mean ± SD of three independent experiments; * *P* < 0.05, ** *P* < 0.01, *** *P* < 0.001

Finally, we performed “rescue” experiments to investigate the role of CCL2 in LINC00330-mediated TAM reprogramming. We overexpressed or knocked down LINC00330 in EC109 cells and subsequently overexpressed or knocked down CCL2 (Fig. 7A, and Figure S6A&E). We collected conditioned media under these various conditions. These conditioned media were then cocultured with M0 macrophages (Fig. 7B, and Additional file 3: Figure S6B&F), M1 macrophages (Fig. 7C and Additional file 3: Figure S6C&G), and M2 macrophages (Fig. 7D and Additional file 3: Figure S6D, H). We assessed the expression of M1 and M2 markers by RT‒PCR. The results indicated that overexpression of LINC00330 promoted the polarization of various types of macrophages toward the M1 phenotype, as evidenced by increased expression of M1 markers and decreased expression of M2 markers. Conversely, knocking down LINC00330 promoted M2 polarization and inhibited M1 macrophage accumulation. Overexpression of CCL2 reversed the LINC00330-mediated reprogramming of TAMs, which was characterized by inhibited M1 polarization and significantly increased M2 polarization. Knocking down CCL2 further promoted polarization toward the M1 phenotype and suppressed the expression of M2 markers; however, overexpression of CCL2 aggravated the M2 polarization of TAMs caused by knockdown of LINC00330, promoting TAM reprogramming. Knocking down CCL2 somewhat alleviated the M2 polarization caused by sh-LINC00330 and promoted TAM reprogramming toward M1 (Fig. 7 and Additional file 3: Figure S6).

**Fig. 8:**
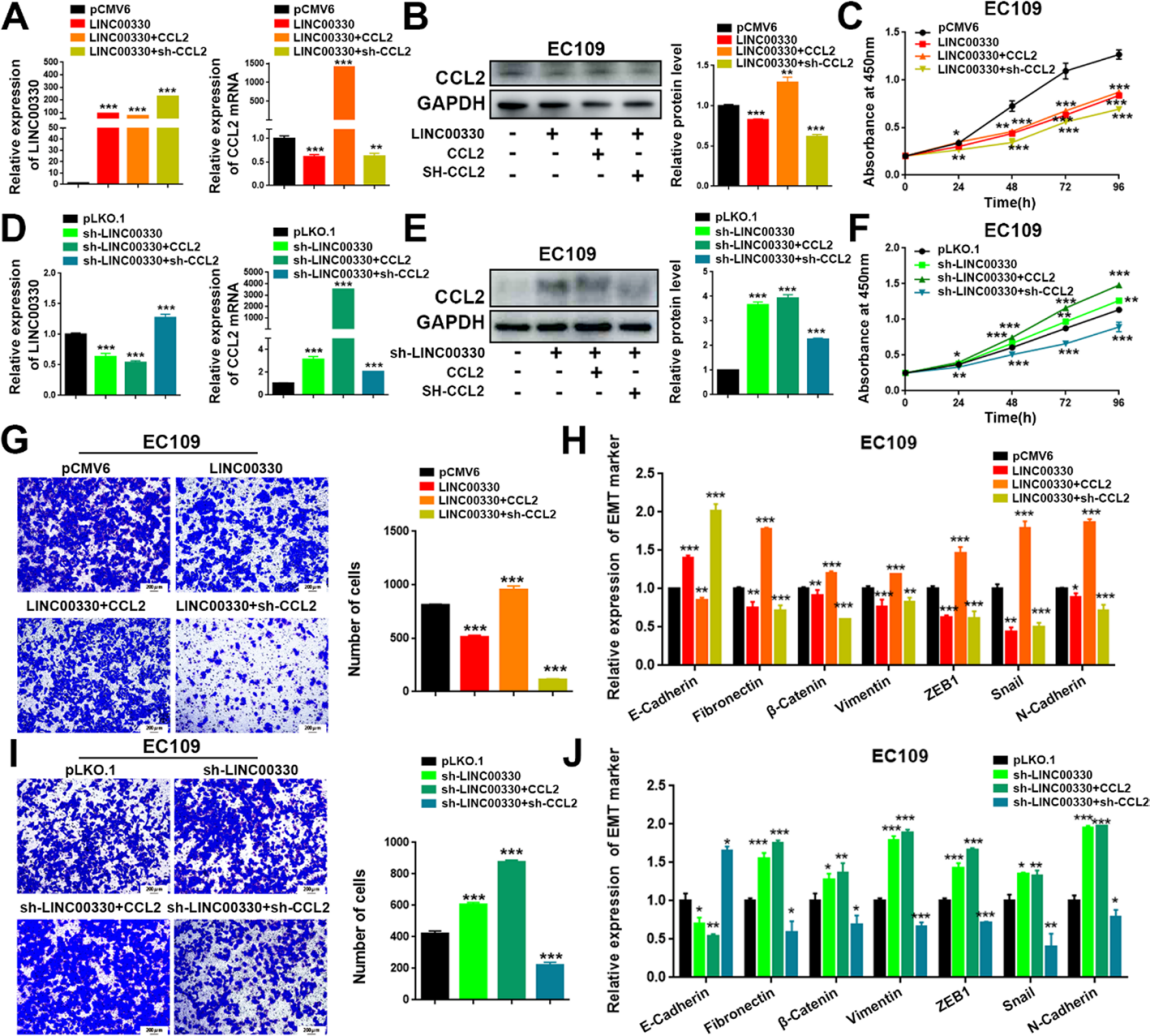
CCL2 plays an important role in LINC00330-mediated ESCC progression. **A**, **D** RT–PCR was used to detect the expression levels of LINC00330 and CCL2 mRNA in EC109 cells. **B**, **E** WB was used to detect the expression level of CCL2 protein in EC109 cells. **C**, **F** CCK-8 assay was used to detect the effect of different treatments on cell proliferation in EC109 cells. **G**, **I** Transwell assays were used to detect the effect of different treatments on the invasion of EC109 cells. Scale bar , 200 μm. **H**, **J** RT‒PCR was used to detect the effect of different treatments on the EMT ability of EC109 cells. * *P* < 0.05, ** *P* < 0.01, *** *P* < 0.001

We also detected the critical role of CCL2 in LINC00330-mediated ESCC progression. Initially, we overexpressed and knocked down CCL2 on the basis of LINC00330 overexpression (Fig. 8A, B, and Additional file 3: Figure S7A&B) and evaluated proliferation, invasion, and EMT of ESCC cells. The results revealed that overexpression of CCL2 could reverse the inhibitory effects of LINC00330 on ESCC cell proliferation (Fig. 8C, and Additional file 3: Figure S7C), invasion (Fig. 8G, and Additional file 3: Figure S7G), and EMT (Fig. 8H, and Additional file 3: Figure S7H). Conversely, knockdown of CCL2 further enhanced the anticancer effects mediated by LINC00330.

Additionally, we overexpressed or knocked down CCL2 on the basis of LINC00330 knockdown (Fig. 8D&E, and Additional file 3: Figure S7D&E) and assessed their impact on ESCC cell proliferation, invasion, and EMT. The results indicated that overexpression of CCL2 significantly exacerbated the promoting effects of LINC00330 knockdown on ESCC cell proliferation (Fig. 8F, and Additional file 3: Figure S7F), invasion (Fig. 8I, and Additional file 3: Figure S7I), and EMT (Fig. 8J, and Additional file 3: Figure S7J). Conversely, knockdown of CCL2 partially reversed this phenomenon. In summary, our study demonstrates that CCL2 is critical for LINC00330-mediated TAM reprogramming and ESCC progression.

**Fig. 9:**
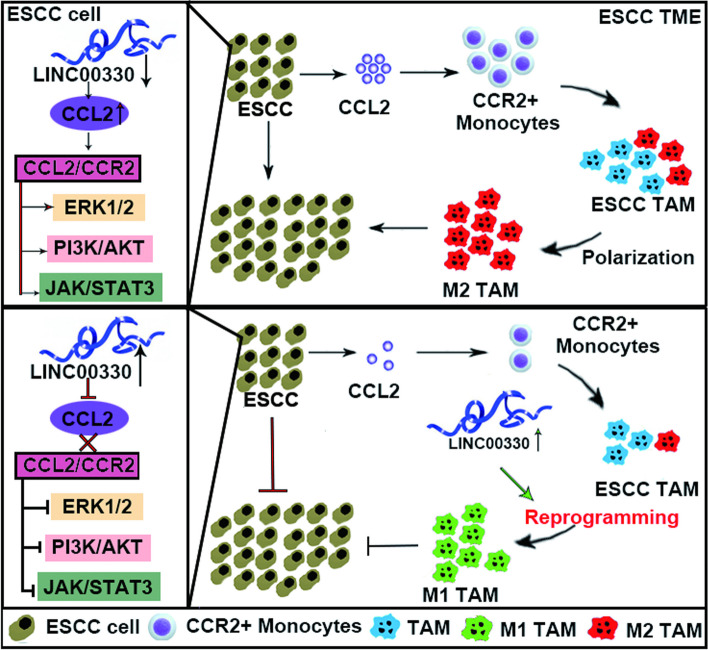
Graphical Abstract showing that LINC00330 inhibited ESCC progression by downregulating the CCL2/CCR2 signaling pathway via the autocrine pathway and suppressed the deterioration of ESCC by mediating TAM reprogramming in the TME via the paracrine pathway

In conclusion, as shown in the “Graphical Abstract” (Fig. 9), our study showed that LINC00330 was expressed at significantly lower levels in ESCC. Low expression of LINC00330 can upregulate the expression of endogenous CCL2 and activate downstream signaling pathways, leading to the deterioration of ESCC. Deteriorated ESCCs secrete CCL2 and recruit CCR2+ mononuclear cells to reside in ESCC tissues to form TAMs [28] and promotes the polarization of TAMs to the M2 phenotype in the ESCC TME, further promoting ESCC immune escape and progression [14]. Conversely, overexpression of LINC00330 in ESCC can suppress CCL2 expression, block the binding of CCL2 to CCR2 and activate its downstream signaling pathway to a certain extent, ultimately promoting a reduction in ESCC malignancy. Additionally, LINC00330 in the ESCC TME can also inhibit the formation of CCL2-mediated M2 TAMs, promoting the reprogramming of ESCC TAMs into anticancer M1 TAMs and further reducing the malignancy of ESCC.

## Discussion

Recently, an increasing number of studies have shown that a comprehensive understanding of the molecular composition and pathogenesis of ESCC requires attention not only to tumor cells, but also to the TME [29]. TAMs are the predominant innate immune cells within the TME, constituting approximately 30–50% of the infiltrating immune cells [30]. They form the crux of immunosuppressive networks and cytokine milieus, significantly affecting tumor progression, metastasis, immunomodulation, angiogenesis, TME remodeling, and the response to cancer therapy [31]. In our study, through single-cell sequencing analysis and CIBERSORTx analysis, we found that there was a large amount of macrophage infiltration in the ESCC TME and that the degree of M2 polarization in ESCC tissues was greater than that in adjacent tissues, which was consistent with existing reports [12, 25], indicating the presence of a mechanism of action in ESCC cells that promotes the polarization of TAMs to M2 macrophages. Therefore, identifying novel biomarkers to induce TAM reprogramming (promoting M2-type TAM redifferentiation, weakening the protumor effects of M2-type TAMs, or increasing the number of tumor-killing M1-type TAMs in ESCC) and delay tumor development may be feasible.

LncRNAs have emerged as critical regulatory molecules in cancer biology. Owing to their wide range of expression profiles, high tumor specificity, and stability in circulating body fluids, lncRNAs are uniquely positioned to screen for tumor diagnostic and prognostic markers [17, 32]. Studies have indicated that dysregulation of lncRNAs is often associated with clinical features, such as tumor size, lymphatic metastasis, and tumor stage, which are closely related to the progression of multiple cancers [17]. LncRNAs can act as oncogenes or tumor suppressors, regulating the physiological and pathological processes of tumor cells [32, 33]. In recent years, an increasing number of studies have confirmed that lncRNAs are significant modulators of macrophage polarization [34]. LncRNAs can affect tumor progression by regulating TAM infiltration, activation, polarization, and functional reprogramming [34, 35]. For instance, lncRNA-HOXC-AS2 regulates TAM polarization through the STAT1/SOCS1 and STAT1/CIITA pathways to promote the progression of non-small cell lung cancer [36]. The NR_109/FUBP1/c-Myc axis regulates TAM polarization and remodels the TME to promote cancer development [37]. However, there is limited research on the regulatory roles of lncRNAs in macrophage activation and polarization in ESCC and the mechanisms by which they mediate TAM reprogramming. In our study, we identified six ESCC TAM-associated lncRNAs—two upregulated (NR2F1-AS1 and ZFHX4-AS1) and four downregulated (HAND2-AS1, LINC00330, RP11-834C11.4, and TTTY10)—that are solely related to TAM infiltration, with no correlation or a weak correlation with the infiltration of other immune cells. Through a literature review, bioinformatics analysis, and preliminary experimental testing, we ultimately focused on LINC00330.

LINC00330 is a poorly characterized intergenic long noncoding RNA, and only a few reports have suggested that LINC00330 may be a key regulator of tumor origin and progression and has potential as a prognostic biomarker [24]. It has also been proposed that LINC00330 may be related to the activation of signaling pathways, such as the PI3K/Akt pathway [23]. However, owing to research limitations, more studies are needed to characterize the function and mechanisms of LINC00330 in different tumors. In our study, we found that LINC00330 was significantly downregulated in ESCC tissues and that low expression of LINC00330 was strongly associated with poor prognosis. LINC00330 functions as a tumor suppressor gene in ESCC. LINC00330 overexpression inhibited ESCC progression in vitro and in vivo. These findings highlight the crucial role of LINC00330 in ESCC progression, suggesting its potential as a novel therapeutic target and biomarker for ESCC. In addition, we also found that LINC00330 is a novel regulator of TAM reprogramming. The expression of LINC00330 was negatively correlated with the panmacrophage markers CD106 and CD81, significantly positively correlated with the M1 macrophage markers IL-12α and IRF5, and negatively correlated with the M2 macrophage markers CD209, CD163, CD206, and IL-10. Coculture experiments of ESCC cells with macrophages revealed that upregulating LINC00330 expression activated M1 polarization while inhibiting M2 polarization. Conversely, knocking down LINC00330 resulted in the opposite phenotype. These findings suggest that LINC00330 can reset macrophage polarization in the esophageal cancer microenvironment and promote TAM reprogramming toward the M1 phenotype. These findings suggest that activating or upregulating LINC00330 may represent a novel therapeutic strategy for targeting TAMs. Additionally, numerous studies have established that lncRNAs have a modular structure and are rich in repeats that are associated with their function [32]. Many lncRNAs interact with chromatin modification complexes, are transcribed from enhancers and nuclear condensates and are closely related to nuclear gene expression and spatial control [32]. LncRNAs also play important roles both inside and outside the cytoplasm, including in the regulation of translation, metabolism, and signaling [16, 38]. Our study revealed that LINC00330 is localized in the cytoplasm. LINC00330 can bind to CCL2 proteins that colocalize with the cytoplasm to form RNA‒protein complexes and inhibit the translation of CCL2 proteins. However, the structure of LINC00330 still needs to be analyzed, and more functions need to be studied in depth.

CCL2 is also known as monocyte chemoattractant protein-1 (MCP-1). Earlier studies aimed to employ MCP-1 as an inducer of macrophage-mediated tumor cell cytotoxicity, indicating that MCP-1 contributes to the induction of antitumor activity [39]. However, an increasing number of recent studies strongly suggest that CCL2 acts as a tumor-promoting factor [41]. Elevated levels of CCL2 are often associated with the progression of diseases, such as esophageal cancer, breast cancer, colorectal cancer, prostate cancer, melanoma, gastric cancer, and ovarian cancer [40]. Excessive expression of CCL2 or the presence of CCL2 genetic variants correlates with poor patient prognosis [22]. Moreover, CCL2-CCR2 signaling is a crucial initial event leading to cancer metastasis [27]. The binding of CCL2 to the CCR2 receptor initiates various signaling pathways that stimulate cell migration and mediate tumor pathogenesis [27]. In addition, as the most well-known CC chemokine, CCL2 exhibits chemotactic activity toward monocytes and basophils, playing a key role in regulating the migration and infiltration of monocytes/macrophages [27, 41]. In solid tumor TMEs, CCL2 can interact with its receptors to recruit CCR2^+^ TAMs, MDSCs, and Th2 cells, creating an immunosuppressive microenvironment that favors tumor progression and accelerates the colonization and growth of metastatic tumor cells, thus contributing to tumor development [42]. Inhibition of CCL2 depletes inflammatory monocytes and macrophages, reducing tumor growth and dissemination in different experimental models, such as prostate cancer, melanoma, breast cancer, lung cancer, and liver cancer [28]. In this study, we discovered that CCL2 is significantly overexpressed in ESCC tumor tissues, and its high expression is closely associated with advanced TNM staging and poor survival prognosis, confirming its oncogenic role in ESCC [14, 27]. Furthermore, we found that LINC00330 can target CCL2 in an autocrine manner to inhibit the interaction between CCL2 and CCR2 and the activation of downstream signaling pathways. LINC00330/CCL2 can also circulate in the ESCC TME in a paracrine fashion, mediating TAM reprogramming through regulation of the LINC00330/CCL2 axis. In conclusion, our study suggested that the LINC00330/CCL2 axis has potential value in the diagnosis and treatment of ESCC and deserves further discussion.

## Conclusions

LINC00330 is not only a critical inhibitor of the progression of ESCC but also a regulator of TAM reprogramming. LINC00330 inhibited ESCC progression by disrupting the CCL2/CCR2 axis and its downstream signaling pathways in an autocrine fashion and by impeding CCL2-mediated TAM reprogramming in a paracrine manner. The new mechanism of TAM reprogramming mediated by the LINC00330/CCL2 axis may provide potential strategies for targeted and immunocombo therapies for patients with ESCC.

### Supplementary Information

Supplementary Material 1.

Supplementary Material 2.

Supplementary Material 3.

Supplementary Material 4.

## Data Availability

The data supporting the conclusions of this article are available from the corresponding author on reasonable request.

## Abbreviations

ESCC: Esophageal squamous cell carcinoma
TAMs: Tumor-associated macrophages
TME: Tumor microenvironment
LncRNAs: Long noncoding RNAs
EMT: Epithelial–mesenchymal transition
DEGs: Differentially expressed genes
ESCC DE-lncRNAs: Differentially expressed lncRNAs in ESCC
Imm-LncRNAs: Immune-associated lncRNAs
ESCC Imm-LncRNAs: Immune-related DE-lncRNAs in ESCC
M1 TAMs: M1 macrophages
M2 TAMs: M2 macrophages
CCL2: C–C motif ligand 2
CCR2: C–C motif chemokine receptor 2
NC: Negative control
FBS: Fetal bovine serum
TCGA: The Cancer Genome Atlas
GEO: Gene Expression Database
RT–PCR: Real-time polymerase chain reaction
WB: Western blotting
RIP: RNA immunoprecipitation

## References

[1] An L, Li M, Jia Q (2023). Mechanisms of radiotherapy resistance and radiosensitization strategies for esophageal squamous cell carcinoma. Mol Cancer.

[2] Da Silva LL, Aguiar PN, de Lima Lopes G (2021). Immunotherapy for advanced esophageal squamous cell carcinoma-renewed enthusiasm and a lingering challenge. JAMA Oncol.

[3] Chen Y, Yu R, Liu Y (2023). Combine radiotherapy and immunotherapy in esophageal squamous cell carcinoma. Crit Rev Oncol Hematol.

[4] Li R, Huang B, Tian H, Sun Z (2022). Immune evasion in esophageal squamous cell cancer: from the perspective of tumor microenvironment. Front Oncol.

[5] de Visser KE, Joyce JA (2023). The evolving tumor microenvironment: from cancer initiation to metastatic outgrowth. Cancer Cell.

[6] Zheng S, Liu B, Guan X (2022). The role of tumor microenvironment in invasion and metastasis of esophageal squamous cell carcinoma. Front Oncol.

[7] Zhang H, Liu L, Liu J, Dang P, Hu S, Yuan W, Sun Z, Liu Y, Wang C (2023). Roles of tumor-associated macrophages in anti-PD-1/PD-L1 immunotherapy for solid cancers. Mol Cancer.

[8] Kumari N, Choi SH (2022). Tumor-associated macrophages in cancer: recent advancements in cancer nanoimmunotherapies. J Exp Clin Cancer Res.

[9] Cheng N, Bai X, Shu Y, Ahmad O, Shen P (2021). Targeting tumor-associated macrophages as an antitumor strategy. Biochem Pharmacol.

[10] Yunna C, Mengru H, Lei W, Weidong C (2020). Macrophage M1/M2 polarization. Eur J Pharmacol.

[11] Cassetta L, Pollard JW (2023). A timeline of tumour-associated macrophage biology. Nat Rev Cancer.

[12] Zheng Y, Chen Z, Han Y, Han L, Zou X, Zhou B, Hu R, Hao J, Bai S, Xiao H, Li WV, Bueker A, Ma Y, Xie G, Yang J, Chen S, Li H, Cao J, Shen L (2020). Immune suppressive landscape in the human esophageal squamous cell carcinoma microenvironment. Nat Commun.

[13] Wang Y, Lyu Z, Qin Y, Wang X, Sun L, Zhang Y, Gong L, Wu S, Han S, Tang Y, Jia Y, Kwong DL, Kam N, Guan XY (2020). FOXO1 promotes tumor progression by increased M2 macrophage infiltration in esophageal squamous cell carcinoma. Theranostics.

[14] Yang H, Zhang Q, Xu M, Wang L, Chen X, Feng Y, Li Y, Zhang X, Cui W, Jia X (2020). CCL2-CCR2 axis recruits tumor associated macrophages to induce immune evasion through PD-1 signaling in esophageal carcinogenesis. Mol Cancer.

[15] Zhang J, Dong Y, Di S, Xie S, Fan B, Gong T (2023). Tumor associated macrophages in esophageal squamous carcinoma: promising therapeutic implications. Biomed Pharmacother.

[16] Winkler L, Dimitrova N (2022). A mechanistic view of long noncoding RNAs in cancer. Wiley Interdiscip Rev RNA.

[17] Adnane S, Marino A, Leucci E (2022). LncRNAs in human cancers: signal from noise. Trends Cell Biol.

[18] Park EG, Pyo SJ, Cui Y, Yoon SH, Nam JW (2022). Tumor immune microenvironment lncRNAs. Brief Bioinform.

[19] Mohapatra S, Pioppini C, Ozpolat B, Calin GA (2021). Non-coding RNAs regulation of macrophage polarization in cancer. Mol Cancer.

[20] Lim YH, Yoon G, Ryu Y, Jeong D, Song J, Kim YS, Ahn Y, Kook H, Kim YK (2023). Human lncRNA SUGCT-AS1 regulates the proinflammatory response of macrophage. Int J Mol Sci.

[21] Arab I, Park J, Shin JJ, Shin HS, Suk K, Lee WH (2023). Macrophage lncRNAs in cancer development: Long-awaited therapeutic targets. Biochem Pharmacol.

[22] Liu J, Liu ZX, Li JJ, Zeng ZL, Wang JH, Luo XJ, Wong CW, Zheng JB, Pu HY, Mo HY, Sheng H, Wu QN, Li H, Wan G, Li B, Wang DS, Xu RH, Ju HQ (2023). The Macrophage-associated LncRNA MALR facilitates ILF3 liquid-liquid phase separation to promote HIF1alpha signaling in esophageal cancer. Cancer Res.

[23] Nie Q, Cao H, Yang J, Liu T, Wang B (2023). PI3K/Akt signalling pathway-associated long noncoding RNA signature predicts the prognosis of laryngeal cancer patients. Sci Rep.

[24] Zhu N, Hou J, Wu Y, Liu J, Li G, Zhao W, Ma G, Chen B, Song Y (2018). Integrated analysis of a competing endogenous RNA network reveals key lncRNAs as potential prognostic biomarkers for human bladder cancer. Medicine (Baltimore).

[25] Zhang X, Peng L, Luo Y, Zhang S, Pu Y, Chen Y, Guo W, Yao J, Shao M, Fan W, Cui Q, Xi Y, Sun Y, Niu X, Zhao X, Chen L, Wang Y, Liu Y, Yang X, Wang C, Zhong C, Tan W, Wang J, Wu C, Lin D (2021). Dissecting esophageal squamous-cell carcinoma ecosystem by single-cell transcriptomic analysis. Nat Commun.

[26] Zhang SM, Wei CY, Wang Q, Wang L, Lu L, Qi FZ (2021). M2-polarized macrophages mediate wound healing by regulating connective tissue growth factor via AKT, ERK1/2, and STAT3 signaling pathways. Mol Biol Rep.

[27] Xu M, Wang Y, Xia R, Wei Y, Wei X (2021). Role of the CCL2-CCR2 signalling axis in cancer: mechanisms and therapeutic targeting. Cell Prolif.

[28] O’Connor T, Heikenwalder M (2021). CCL2 in the tumor microenvironment. Adv Exp Med Biol.

[29] Bilotta MT, Antignani A, Fitzgerald DJ (2022). Managing the TME to improve the efficacy of cancer therapy. Front Immunol.

[30] Lopez-Yrigoyen M, Cassetta L, Pollard JW (2020). Macrophage targeting in cancer. Ann N Y Acad Sci.

[31] Song J, Xiao T, Li M, Jia Q (2023). Tumor-associated macrophages: potential therapeutic targets and diagnostic markers in cancer. Pathol Res Pract.

[32] Mattick JS, Amaral PP, Carninci P, Carpenter S, Chang HY, Chen LL, Chen R, Dean C, Dinger ME, Fitzgerald KA, Gingeras TR, Guttman M, Hirose T, Huarte M, Johnson R, Kanduri C, Kapranov P, Lawrence JB, Lee JT, Mendell JT, Mercer TR, Moore KJ, Nakagawa S, Rinn JL, Spector DL, Ulitsky I, Wan Y, Wilusz JE, Wu M (2023). Long non-coding RNAs: definitions, functions, challenges and recommendations. Nat Rev Mol Cell Biol.

[33] Anderson KM, Anderson DM (2022). LncRNAs at the heart of development and disease. Mamm Genome.

[34] Xu J, Liu XY, Zhang Q, Liu H, Zhang P, Tian ZB, Zhang CP, Li XY (2021). Crosstalk among YAP, LncRNA, and tumor-associated macrophages in tumorigenesis development. Front Oncol.

[35] Zhou Z, Wang Z, Gao J, Lin Z, Wang Y, Shan P, Li M, Zhou T, Li P (2022). Noncoding RNA-mediated macrophage and cancer cell crosstalk in hepatocellular carcinoma. Mol Ther Oncolytics.

[36] Yin C, Li J, Li S, Yang X, Lu Y, Wang C, Liu B (2024). LncRNA-HOXC-AS2 regulates tumor-associated macrophage polarization through the STAT1/SOCS1 and STAT1/CIITA pathways to promote the progression of non-small cell lung cancer. Cell Signal.

[37] Zhang C, Wei S, Dai S, Li X, Wang H, Zhang H, Sun G, Shan B, Zhao L (2023). The NR_109/FUBP1/c-Myc axis regulates TAM polarization and remodels the tumor microenvironment to promote cancer development. J Immunother Cancer.

[38] Choudhuri S (2023). Long noncoding RNAs: biogenesis, regulation, function, and their emerging significance in toxicology, Toxicol Mech. Methods.

[39] Manome Y, Wen PY, Hershowitz A, Tanaka T, Rollins BJ, Kufe DW, Fine HA (1995). Monocyte chemoattractant protein-1 (MCP-1) gene transduction: an effective tumor vaccine strategy for non-intracranial tumors. Cancer Immunol Immunother.

[40] Yoshimura T, Li C, Wang Y, Matsukawa A (2023). The chemokine monocyte chemoattractant protein-1/CCL2 is a promoter of breast cancer metastasis. Cell Mol Immunol.

[41] Toya M, Zhang N, Tsubosaka M, Kushioka J, Gao Q, Li X, Chow SK, Goodman SB (2023). CCL2 promotes osteogenesis by facilitating macrophage migration during acute inflammation. Front Cell Dev Biol.

[42] Li X, He G, Liu J, Yan M, Shen M, Xu L, An M, Huang J, Gao Z (2022). CCL2-mediated monocytes regulate immune checkpoint blockade resistance in pancreatic cancer. Int Immunopharmacol.

