# Supplementary material for: LINC00330/CCL2 axis-mediated ESCC TAM reprogramming affects tumor progression
Source: Cell Mol Biol Lett. 2024 May 20;29:77. doi: 10.1186/s11658-024-00592-8 (PMC11103861; doi:10.1186/s11658-024-00592-8)
Supplement: Supplementary file 2 — Supplementary Material 2. [file 11658_2024_592_MOESM2_ESM.docx]

**Additional file 2. List of primers sequences used in this research.**

**A. Primer for construct knockdown plasmids**

| Gene | Symbol | Sequence |
| --- | --- | --- |
| LINC00330 | LINC00330- sh1 | F:CCGGGTTCCAAGATTAGCCAATTTACTCGAGTAAATTGGCTAATCTTGGAAC TTTTT |
|  |  | R: AATT AAAAAGTTCCAAGATTAGCCAATTTACTC  GAGTAAATTGGCTAATCTTGGAAC |
|  | LINC00330- sh2 | F: CCGG GGTTGCATGAAGACGTAAGTTCTCGAG AACTTACGTCTTCATGCAACC TTTTT |
|  |  | R: AATT AAAAAGGTTGCATGAAGACGTAAGTTC  TCGAGAACTT ACGTCTTCATGCAACC |
|  | LINC00330- sh3 | F: CCGG GTTGCATGAAGACGTAAGTTT CTCGAG  AAACTTACGTCTTCATGCAAC TTTTT |
|  |  | R: AATT AAAAGTTGCATGAAGACGTAAGTTT  CTCGAGAAACTTACGTCTTCATGCAAC |
| CCL2 | CCL2-sh1 | F: CGGGCCCAGTCACCTGCTGTTATAACTC  GAGTTTATAGCATCACGAACTGTCTTTTT |
|  |  | R: AATTAAAAAGACAGTTCGTGATGCTATAAAC  TCGAGTTATAA CAGCAGGTGACTGGG |
|  | CCL2-sh2 | F: CGGGCTCGCGAGCTATAGAAGAATCTCGAG  AATAATCCTCTTGTCTCCATATTTTT |
|  |  | R: AATTAAAAATATGG AGACAAGAGG ATTATT  CTCGAGATTCTT CTATAGCTCGCG AGC |
|  | CCL2-sh3 | F: CGGGCTGTGATCTTCAAGACCATTCTCGAG  AATTGAACGCAGGACCTTCCATTTTT |
|  |  | R: ATTAAAAATGGAAGGTCCTGCGTTCAATTCTC  GAGAATGGTCTTGAAGATCACAGC |

**B. Primer for RT-PCR**

| Species | Gene | Sequence |
| --- | --- | --- |
| Homo sapiens | LINC00330 | F: AGCCAGAAGGTCAGATGTC |
|  |  | R: TTGAAGCTCCTTGTTTGCC |
|  | E-cadherin | F: CACAGCAGAACTAACACACGGG |
|  |  | R: AGTCACCCACCTCTAAGGCCAT |
|  | β-catenin | F: CTTGGACTTGATATTGGTGCCC |
|  |  | R: GGCCATATCCACCAGAGTGAAA |
|  | Fibronectin | F: CAGCAGAGGCATAAGGTTCG |
|  |  | R: TCAAAGCACGAGTCATCCG |
|  | Vimentin | F: TCTGGATTCACTCCCTCTGGTT |
|  |  | R: TCGTGATGCTGAGAAGTTTCGT |
|  | ZEB1 | F: GCTGCCAATAAGCAAACGATTC |
|  |  | R: CCTGATTTCCATTTGGCTGG |
|  | Snail | F: AGACCCTGGTTGCTTCAAGG |
|  |  | R: GACCTGTCTGCAAATGCTCTG |
|  | N-cadherin | F: TGTGGACAGGATTGTGGGTG |
|  |  | R: CGGCGTTTCATCCATACCAC |
|  | CD68 | F: ATTCATGCAGGACCTCCAG |
|  |  | R: TTCTGAGCCGAGAATGTCC |
|  | IL-12 | F: CTGATGGATCCTAAGAGGCAG |
|  |  | R: GTTGAAATTCAGGGCCTGC |
|  | IRF5 | F: AACACCATCTTCAAGGCCT |
|  |  | R: CTCTTGTTAAGGGCACAGC |
|  | CD80 | F: CACTTCTGTTCAGGTGTTATCC |
|  |  | R: AACAGAAACATTGTGACCACAG |
|  | CD86 | F: AGTGCTTGCTAACTTCAGTC |
|  |  | R: CGTGTATAGATGAGCAGGTC |
|  | CD163 | F: TTGCACAGATATTTCAGTGCAG |
|  |  | R: ACTGCAATAAAGGATGACTGAC |
|  | CD206 | F: GATGAAAGGCAAGGATGGG |
|  |  | R: CTTCGTGATTTCATCTTGCAG |
|  | CCL2 | F: CCAGATGCAATCAATGCCC |
|  |  | R: TGGTCTTGAAGATCACAGCT |
|  | CCR2 | F: ACAAGCTGAACAGAGAAAGTG |
|  |  | R: AACCGAGAACGAGATGTGG |
|  | AKT | F: CTTCTTTGCCGGTATCGTG |
|  |  | R: TGCTGTCATCTTGGTCAGG |
|  | ERK1/2 | F: GATCTCAAGATCTGTGACTTTGG |
|  |  | R: CACATATTCTGTCAGGAACCC |
|  | STAT3 | F: CGCACTTTAGATTCATTGATGC |
|  |  | R: AGGTGAGGGACTCAAACTG |
|  | mTORC1 | F: AAACCTTTCCAAGCAAGCA |
|  |  | R: GGGAAGATGTTGATTCCGG |
|  | GAPDH | F: AAGGTGAAGGTCGGAGTCAACGGATTTG |
|  |  | R: CTCGCTCCTGGAAGATGGTGATGGGATT |
| Mus musculus | CD68 | F:CTTTGGATTCAAACAGGACCT |
|  |  | R: AAGGACACATTGTATTCCACC |
|  | CD80 | F: TCAAGTTTCCATGTCCAAGG |
|  |  | R: GTTCATCAACATCTGAAGACAC |
|  | CD86 | F: CTGATCTCAGATGCTGTTTCC |
|  |  | R: AGCCTTTGTAAATGGGCAC |
|  | CD163 | F: TCAGCGACTTACAGTTTCCT |
|  |  | R: TCATCCGCCTTTGAATCCA |
|  | CD206 | F: TACACAAATTCAGGGTTCTGG |
|  |  | R: GATGCTGCTGTTATGTCTCTG |
|  | GAPDH | F: ACTCTTCCACCTTCGATGC |
|  |  | R: CCGTATTCATTGTCATACCAGG |

**C. Primer for LINC00330 RNA pulldown**

| Gene | Symbol | Sequence |
| --- | --- | --- |
| LINC00330 | Forward: | CAGTTGAGAACAGACTCCACCTTG |
|  | Reverse: | AGTAAAGACAGGGTTTCACCGTG |
|  | F+T7: | AAATAATACGACTCACTATAGGGCAGTTGAGAACAGACTCCACCTTG |
|  | R+T7: | AAATAATACGACTCACTATAGGGAGTAAAGACAGGGTTTCACCGTG |
