# Supplementary material for: LINC00330/CCL2 axis-mediated ESCC TAM reprogramming affects tumor progression
Source: Cell Mol Biol Lett. 2024 May 20;29:77. doi: 10.1186/s11658-024-00592-8 (PMC11103861; doi:10.1186/s11658-024-00592-8)
Supplement: Supplementary file 3 — Supplementary Material 3. [file 11658_2024_592_MOESM3_ESM.docx]

**LINC00330/CCL2 axis-mediated ESCC TAM reprogramming affects tumor progression**

Lijun Zhao^1#, *^, Gengchao Wang^2#^，Haonan Qi^1#^, Lili Yu^1^, Huilong Yin^1^, Ruili Sun^1^, Hongfei Wang^1^, Xiaofei Zhu^1*^, Angang Yang^3*^

1 Henan Key Laboratory of Immunology and Targeted Drugs, Xinxiang Key Laboratory of Tumor Microenvironment and Immunotherapy, School of Medical Technology, Xinxiang Medical University, Xinxiang, Henan, China.

2 State Key Laboratory of Liver Research, Department of Pathology, Li Ka Shing Faculty of Medicine, The University of Hong Kong, Hong Kong, China.

3 The State Key Laboratory of Cancer Biology, Department of Immunology, Fourth Military Medical University, Xi’an, Shanxi, China.

^#^ Contributed equally.

*Corresponding author:

[agyang@fmmu.edu.cn,](mailto:agyang@fmmu.edu.cn,) [lijun_zhao123@163.com](mailto:lijun_zhao123@163.com), [zhuxf@xxmu.edu.cn](mailto:zhuxf@xxmu.edu.cn).

**Contents**

1. Supplementary Figure and Legends: ………………………………………2 - 9

2. Supplementary Tables: ……………………………………………. ……… 10 - 12

**1. Supplementary Figure and Legends**


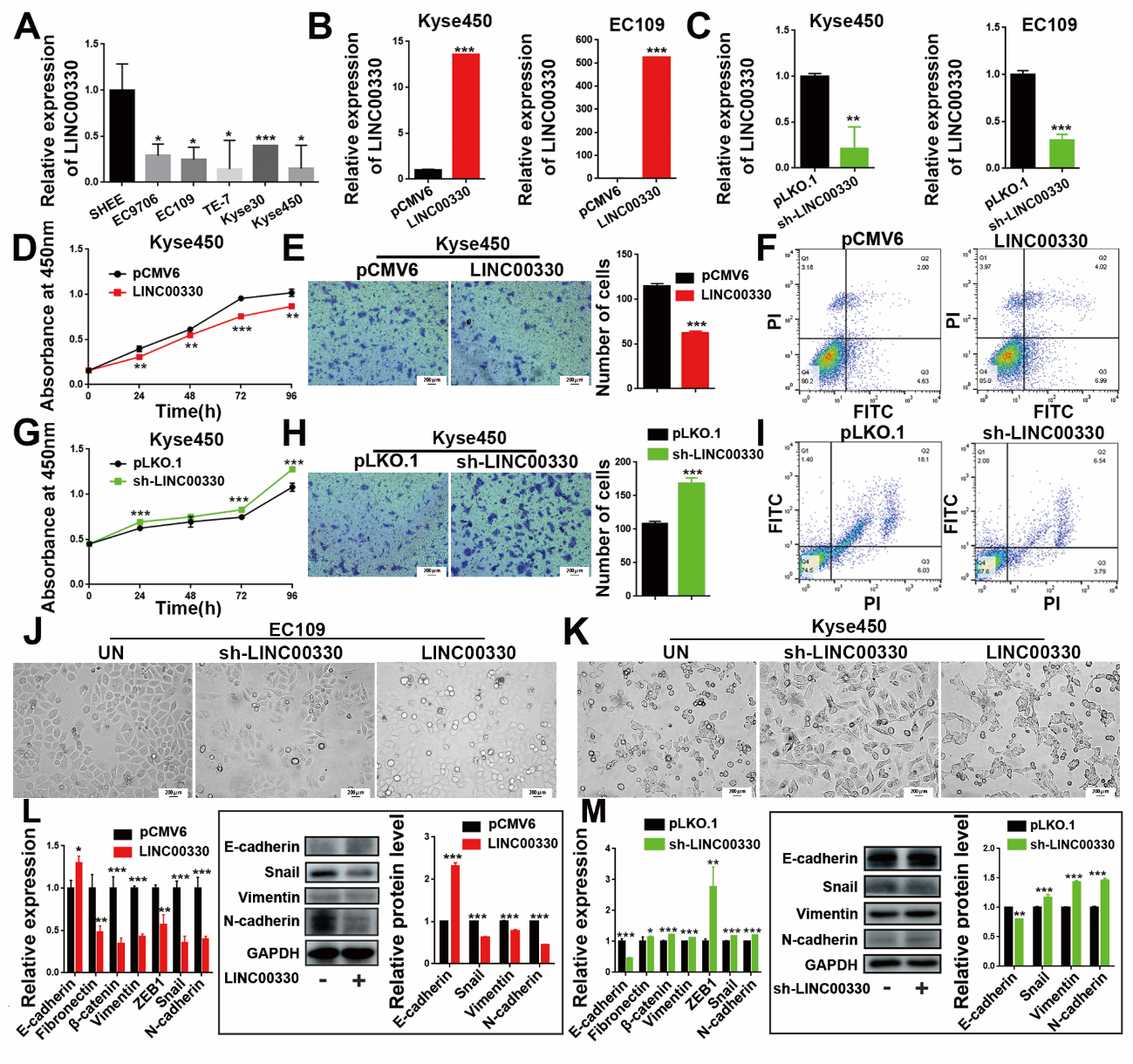


**Figure S1** Overexpression of LINC00330 inhibits Kyse450 cell proliferation and migration. A RT‒PCR was used to detect the expression of LINC00330 in the normal esophageal epithelial cell line SHEE and five ESCC cell lines (EC9706, EC109, TE-7, Kyse30, and Kyse450). B Overexpression of LINC00330 in the ESCC cell lines Kyse450 and EC109. C LINC00330 knockdown in the ESCC cell lines Kyse450 and EC109. D&G A CCK-8 assay was used to detect the effect of LINC00330 overexpression or knockdown on the proliferation of Kyse450 cells. E&H Transwell assays were used to detect the effect of LINC00330 overexpression or knockdown on the invasion of Kyse450 cells. Scale bar = 200 μm. F&I Flow cytometry was used to detect the effect of LINC00330 overexpression or knockdown on Kyse450 cell apoptosis. J&K Morphological evidence of the effect of LINC00330 on EMT. Scale bar = 200 μm. L&M RT‒PCR and WB were used to detect the effect of LINC00330 overexpression or knockdown on the EMT ability of Kyse450 cells. * P <0.05, ** P <0.01, *** P <0.001.


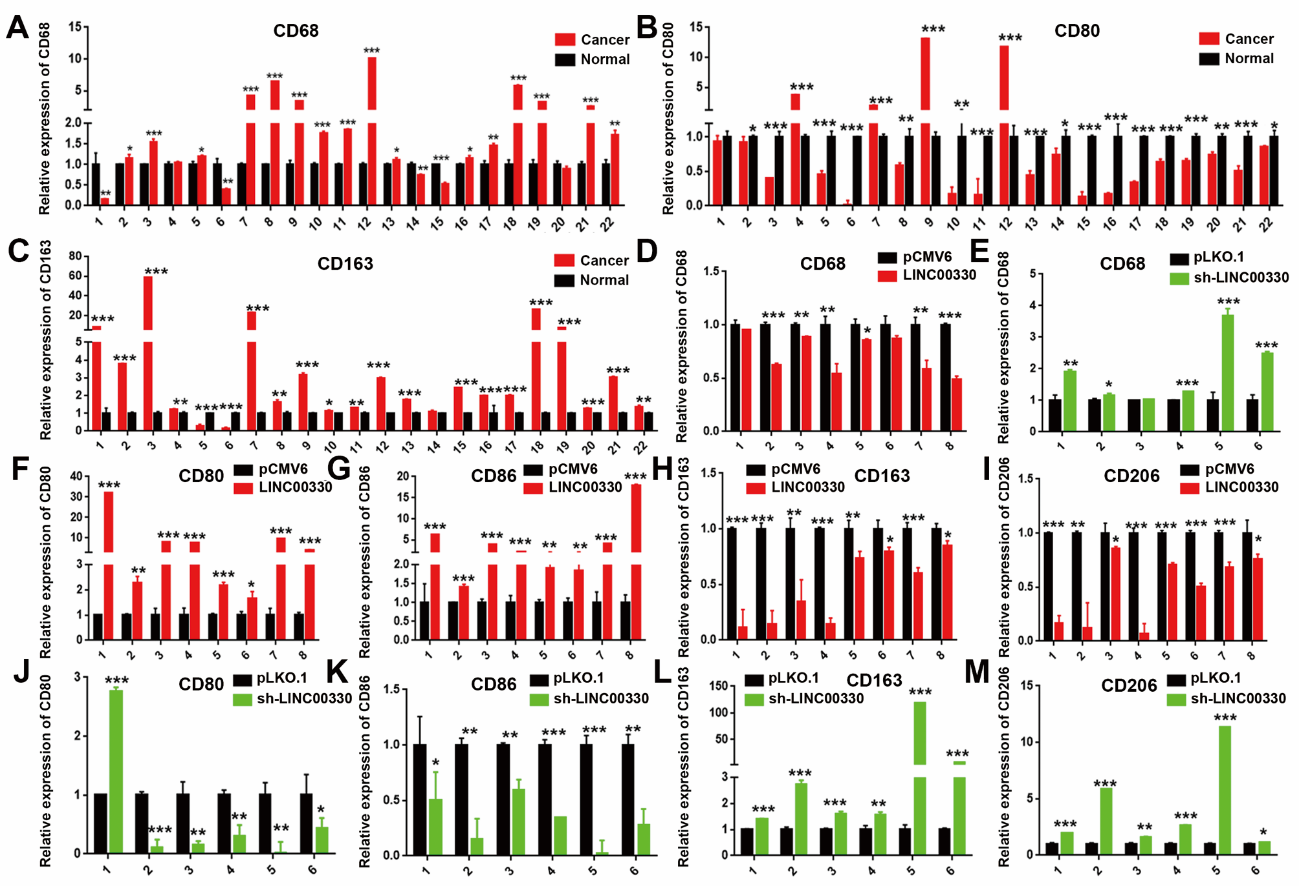


**Figure S2** Evaluation of the relationship between the expression of LINC00330 and TAM infiltration in ESCC clinical samples and animal tumors. **A-C** RT‒PCR was used to detect the expression of the macrophage marker CD68, the M1 marker CD80 and the M2 marker CD163 in ESCC clinical samples. **D&E** RT‒PCR was used to detect the infiltration of macrophages (CD68) in tumor tissues generated by subcutaneous tumorigenesis in nude mice, as shown in Fig. 2L&O. **F-I** Tumor tissue generated from the mice described in Fig. 2L was collected, and RT‒PCR was used to detect the expression of M1 (CD80, CD86) and M2 (CD163, CD206) markers. **J-M** Tumor tissues generated from the mice described in Fig. 2O were collected, and RT‒PCR was used to detect the expression of M1 (CD80, CD86) and M2 (CD163, CD206) markers. * P <0.05, ** P <0.01, *** P <0.001.


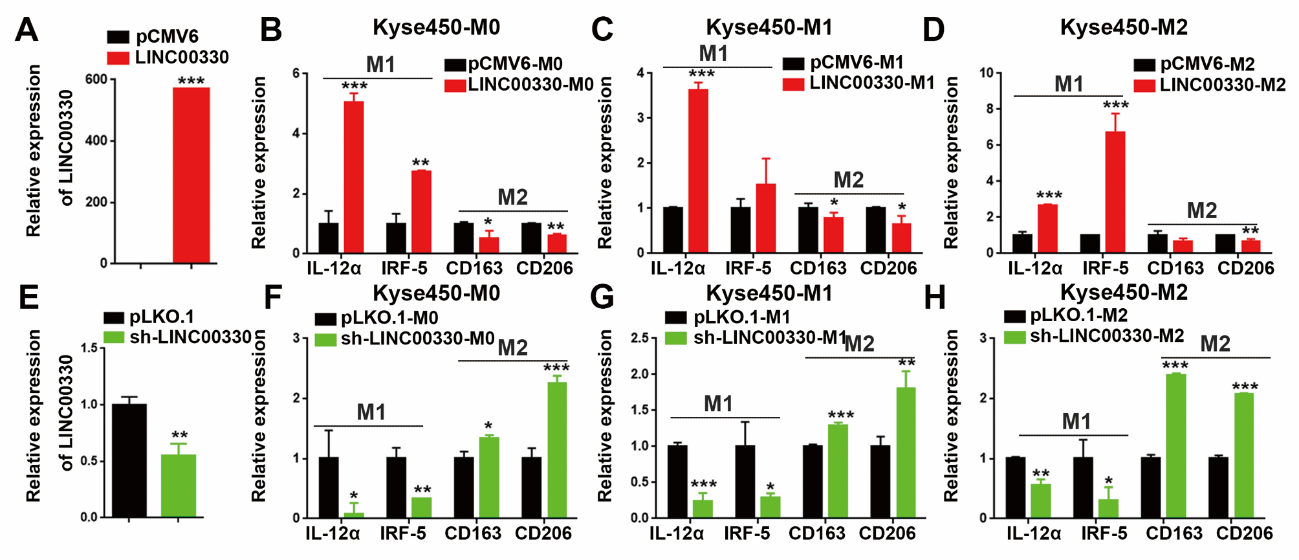


**Figure S3** LINC00330 facilitates TAM reprogramming in Kyse450 cells. **A&E** Overexpression or knockdown of LINC00330 in Kyse450 cells. **B&F** M0 macrophages were cocultured with conditioned medium generated by overexpression or deletion of LINC00330 in Kyse450 cells, and the expression of M1 and M2 markers was detected by RT‒PCR. **C&G** M1 macrophages were cocultured with conditioned medium generated by overexpression or deletion of LINC00330 in Kyse450 cells, and the expression of M1 and M2 markers was detected by RT‒PCR. **D&H** M2 macrophages were cocultured with conditioned medium generated by overexpression or deletion of LINC00330 in Kyse450 cells, and the expression of M1 and M2 markers was detected by RT‒PCR. * P <0.05, ** P <0.01, *** P <0.001.


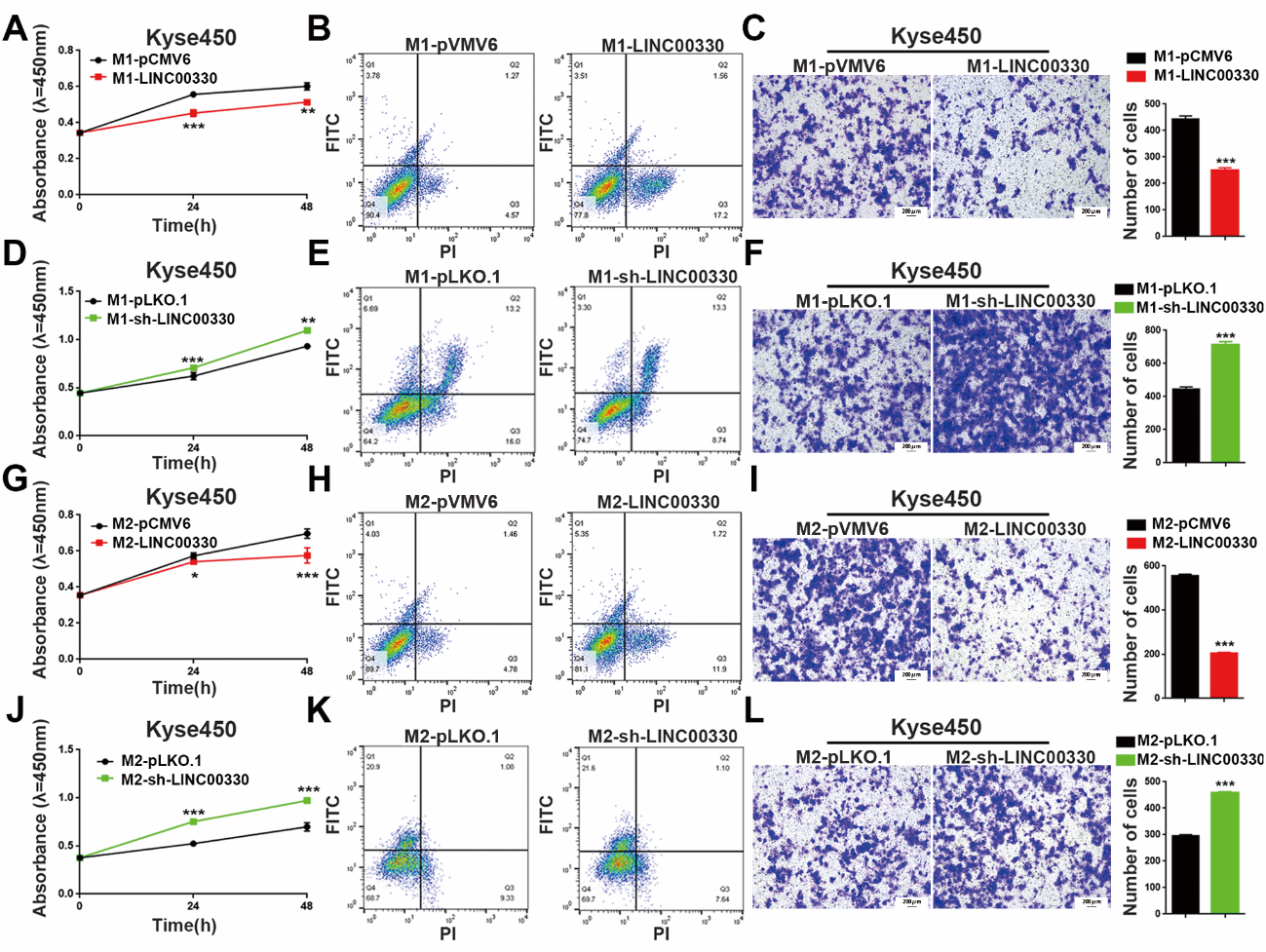


**Figure S4** LINC00330-mediated TAM reprogramming inhibits the progression of ESCC. **A&G** CCK-8 assays were used to detect the effect of LINC00330 overexpression in M1 and M2 macrophages on the proliferation capacity of Kyse450 cells. **B&H** Flow cytometry was used to detect the effect of LINC00330 overexpression in M1 and M2 macrophages on Kyse450 cell apoptosis. **C&I** Transwell assays were used to analyze the effect of LINC00330 overexpression in M1 and M2 macrophages on Kyse450 cell invasion. Scale bar = 200 μm. **D&J** CCK-8 assays were used to detect the effect of LINC00330 knockdown in M1 and M2 macrophages on the proliferation capacity of Kyse450 cells. **E&K** Flow cytometry was used to detect the effect of LINC00330 knockdown in M1 and M2 macrophages on Kyse450 cell apoptosis. **F&L** Transwell assays were used to analyze the effect of LINC00330 knockdown in M1 and M2 macrophages on Kyse450 cell invasion. Scale bar = 200 μm. The figure shows the mean ± SD of three independent experiments; * P <0.05, ** P <0.01, *** P <0.001.


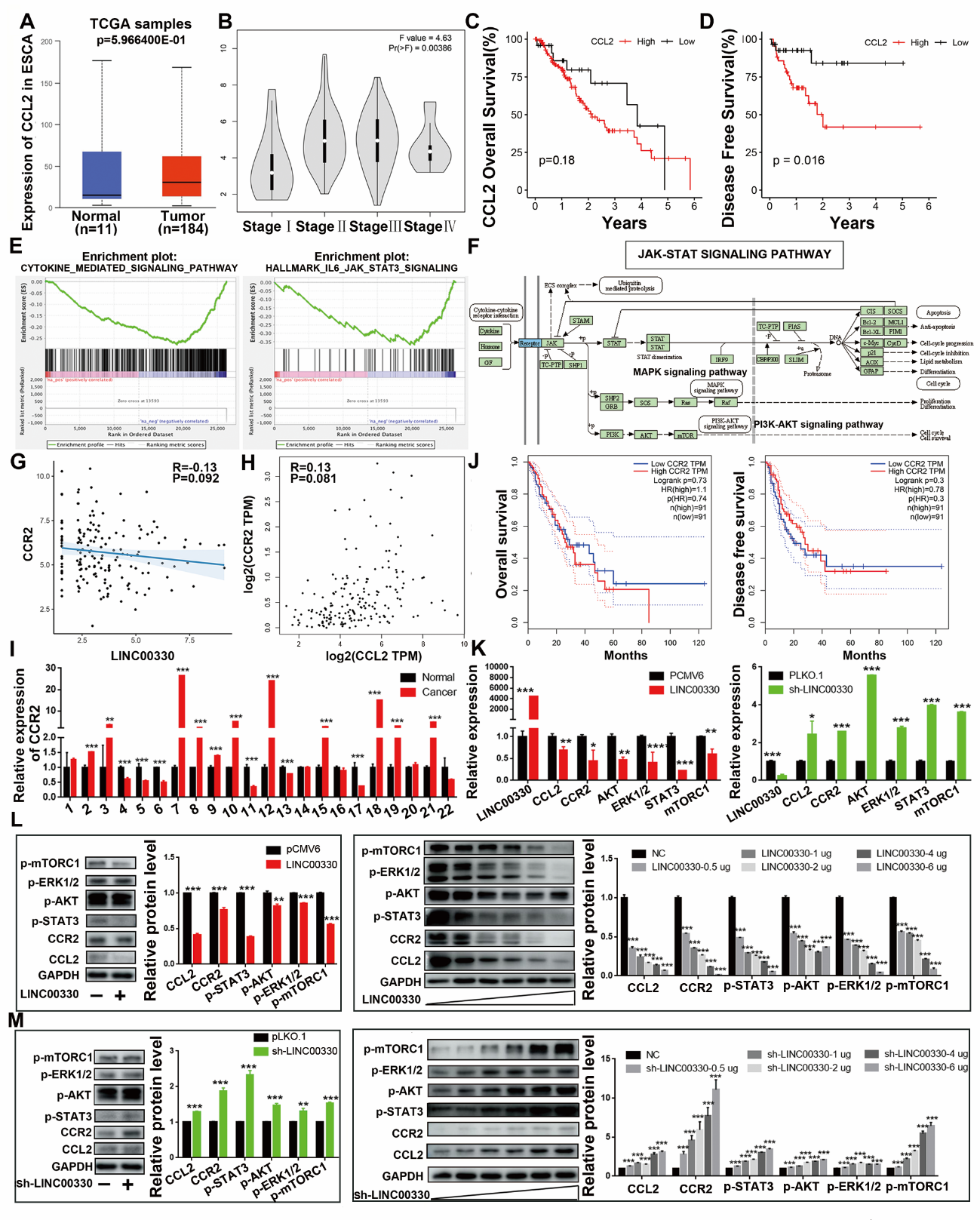


**Figure S5** LINC00330 inhibits the activation of CCL2 and its downstream signaling pathway. **A** The expression of CCL2 in ESCC tissues and adjacent tissues in the TCGA database. **B** The expression of CCL2 at different stages of ESCC in the TCGA database. **C&D** Kaplan‒Meier analysis of the relationship between CCL2 expression and survival prognosis in patients with ESCC. The OS data were obtained from xena, and the RFS data were obtained from cBioPortal. **E** GSEA showed that LINC00330 negatively regulated the "chemokine-mediated signaling pathway" and "IL-6-JAK-STAT signaling". **F** KEGG map showing the "JAK-STAT signaling pathway" involved in LINC00330. **G** Pearson correlation analysis of the correlation between LINC00330 and CCR2 expression in ESCC tumor tissues. **H** Pearson correlation analysis of the correlation between CCL2 and CCR2 expression in ESCC tumor tissues. **I** RT‒PCR was used to detect the expression of CCR2 in 22 ESCC clinical samples. **J** Kaplan‒Meier analysis of the relationship between CCR2 expression and survival prognosis in ESCC patients. **K** RT‒PCR was used to detect the effect of LINC00330 overexpression or knockdown on the CCL2/CCR2 axis and its downstream signaling pathway in Kyse450 cells. **L&M** Western blot analysis of the effect of LINC00330 overexpression or knockdown on the CCL2/CCR2 axis and its downstream signaling pathway in ESCC cells. * P <0.05, ** P <0.01, *** P <0.001.

**
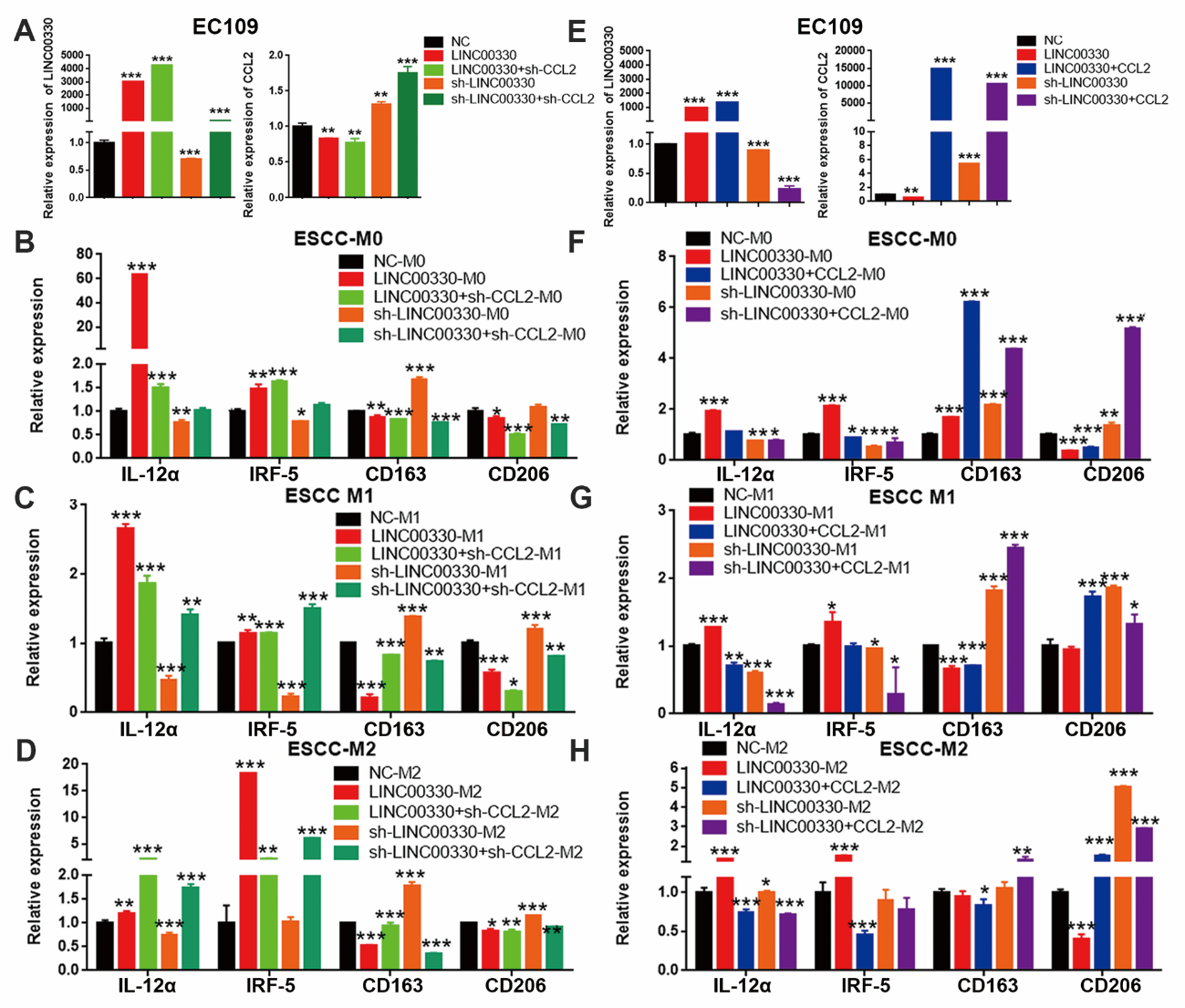
**

**Figure S6** CCL2 can affect LINC00330-mediated TAM reprogramming to some extent. **A&E** RT‒PCR was used to detect the expression of LINC00330 and CCL2 in EC109 cells under different treatment conditions. **B&F** M0 macrophages were cocultured with CM generated by different experimental groups, and the expression of M1 and M2 markers was detected by RT‒PCR. **C&G** M1 macrophages were cocultured with CM generated by different experimental groups, and the expression of M1 and M2 markers was detected by RT‒PCR. **D&H** M2 macrophages were cocultured with CM generated by different experimental groups, and the expression of M1 and M2 markers was detected by RT‒PCR. The figure shows the mean ± SD of three independent experiments, * P <0.05, ** P <0.01, *** P <0.001.


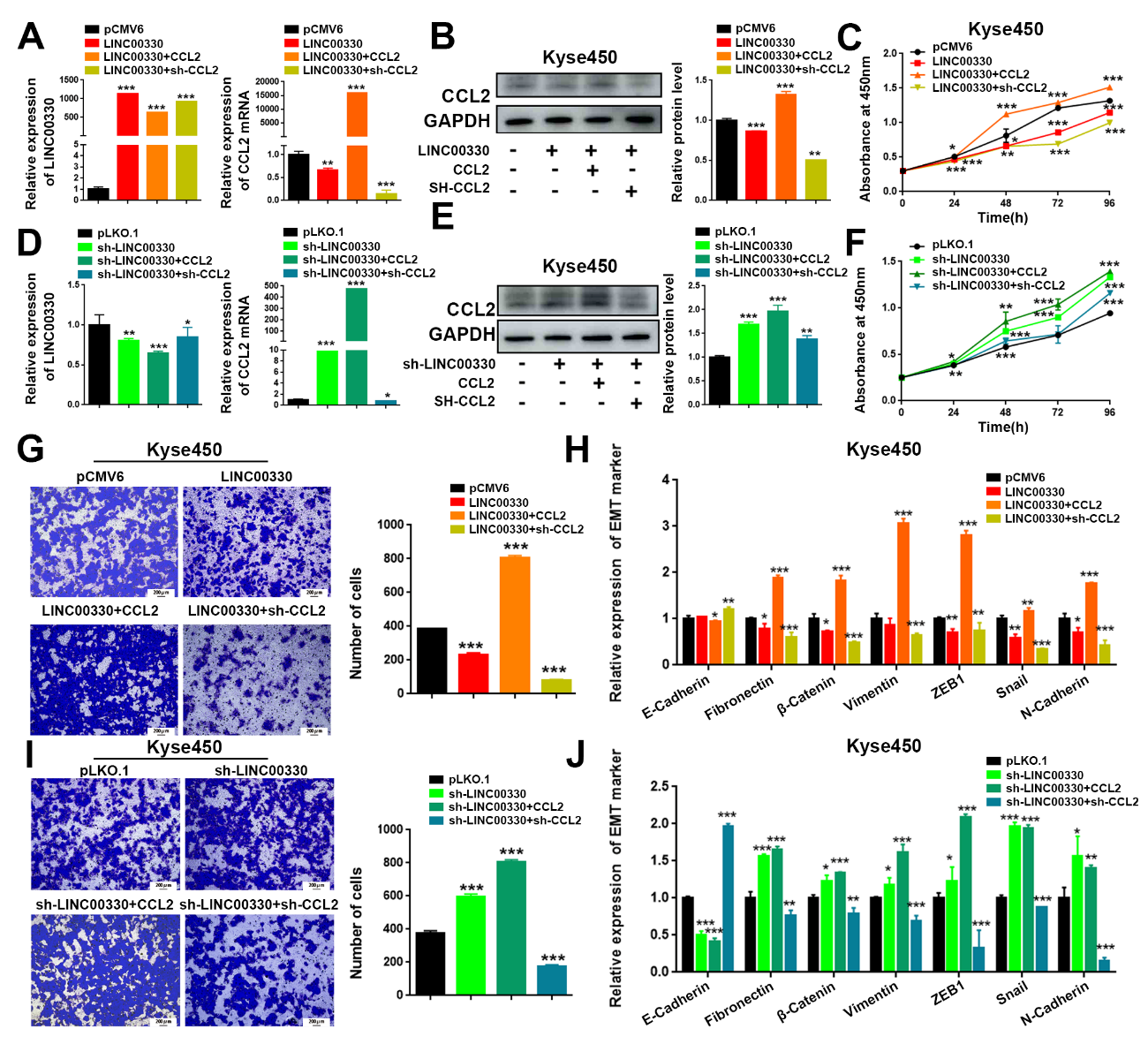


**Figure S7** CCL2 plays an important role in LINC00330-mediated ESCC progression. **A&D** RT-PCR was used to detect the expression levels of LINC00330 and CCL2 mRNA in Kyse450 cells. **B&E** WB was used to detect the expression level of CCL2 protein in Kyse450 cells. **C&F** CCK-8 assay was used to detect the effect of different treatments on cell proliferation in Kyse450 cells. **G&I** Transwell assays were used to detect the effect of different treatments on the invasion of Kyse450 cells. Scale bar = 200 μm. **H&J** RT‒PCR was used to detect the effect of different treatments on the EMT ability of Kyse450 cells. * P <0.05, ** P <0.01, *** P <0.001.

**2. Supplementary Tables**

**Table S1** The correlation between the expression of lncRNAs and the macrophage infiltration level in ESCC.

|  |  | **LncRNA symbol** | | | | | |  |
| --- | --- | --- | --- | --- | --- | --- | --- | --- |
|  |  | **Up** | | **Down** | | | |  |
|  |  | **NR2F1**  **-AS1** | **ZFHX4**  **-AS1** | **HAND2-AS1** | **LINC00330** | **RP11-834C11.4** | **TTTY10** | |
| **CD8**  **Tcell** | P Value | 0.413 | 0.538 | 0.145 | **0.646** | 0.004 | 0.477 | |
|  | Rs Value | -0.065 | 0.049 | 0.115 | **-0.036** | 0.226 | 0.056 | |
| **Dendritic** | P Value | 0.373 | 0.456 | 0.001 | **0.204** | 0.935 | 0.066 | |
|  | Rs Value | 0.071 | 0.059 | 0.259 | **-0.1** | 0.006 | 0.145 | |
| **Neutrophil** | P Value | 0.699 | 0.375 | 0.002 | **0.558** | 0.049 | 0.002 | |
|  | Rs Value | -0.031 | 0.07 | 0.239 | **0.046** | 0.155 | 0.246 | |
| **CD4**  **Tcell** | P Value | 0.195 | 0.339 | 0.04 | **0.373** | 0.164 | 0.001 | |
|  | Rs Value | 0.102 | 0.076 | 0.161 | **-0.07** | 0.11 | 0.261 | |
| **B_cell** | P Value | 0.187 | 0.039 | 0.99 | **0.789** | 0 | 0.001 | |
|  | Rs Value | 0.104 | 0.163 | -0.001 | **-0.021** | 0.271 | 0.258 | |
| **Macrophage** | P Value | 0 | 0 | 0 | **0** | 0 | 0 | |
|  | Rs Value | 0.391 | 0.416 | 0.353 | **-0.33** | 0.31 | 0.329 | |

**Table S2** Correlations between LINC00330 expression and clinicopathological characteristics of 83 ESCC patients in the TCGA cohort.

| Characteristics | n | LINC00330 high expression (42) | LINC00330 low expression (41) | P value |
| --- | --- | --- | --- | --- |
| Age  <60  ≥60 | 49  34 | 24  18 | 25  16 | 0.3230 |
| Sex  Male | 70 | 34 | 36 | 0.7392 |
| Female | 13 | 8 | 5 |  |
| TNM stage  StageⅠ  StageⅡ-Ⅳ | 55  25 | 24  16 | 31  9 | 0.2317 |
| No reported | 3 | 2 | 1 |  |
| Tumor grade  G1-2  G3-4 | 53  19 | 27  10 | 26  9 | 0.0616 |
| Abbreviations: TNM, tumor node metastasis; | | | | |

**Table S3** Correlation analysis between LINC00330 and related immune cell genes and markers using GEPIA.

|  |  | **ESCA** | | | |
| --- | --- | --- | --- | --- | --- |
|  |  | **Tumor** | | **Normal** | |
|  |  | **Rs Value** | **P Value** | **Rs Value** | **P Value** |
| **CD4 T cell** | CCR7 | -0.059 | 0.43 | 0.56 | 0.046 |
| **CD8 T cell** | CD8 | -0.075 | 0.31 | 0.026 | 0.93 |
|  | CD27 | -0.07 | 0.35 | 0.41 | 0.17 |
| **TAM** | CD106 | -0.33 | 6.50E-09 | -0.16 | 0.59 |
|  | CD81 | -0.47 | 6.20E-40 | -0.13 | 0.67 |
| **M1 Macrophages** | IL-12α | 0.56 | 0 | 0.92 | 7.70E-06 |
|  | IRF5 | 0.48 | 0 | 0.97 | 6.60E-08 |
| **M2 Macrophages** | CD209 | -0.27 | 8.50E-16 | -0.18 | 0.56 |
|  | CD163 | -0.24 | 2.10E-10 | -0.17 | 0.58 |
|  | CD206 | -0.27 | 1.60E-13 | -0.25 | 0.41 |
|  | IL10 | -0.2 | 7.90E-08 | 0.31 | 0.3 |
