# Supplementary material for: LINC00330/CCL2 axis-mediated ESCC TAM reprogramming affects tumor progression
Source: Cell Mol Biol Lett. 2024 May 20;29:77. doi: 10.1186/s11658-024-00592-8 (PMC11103861; doi:10.1186/s11658-024-00592-8)
Supplement: Supplementary file 4 — Supplementary Material 4. [file 11658_2024_592_MOESM4_ESM.pptx]

## Slide 1
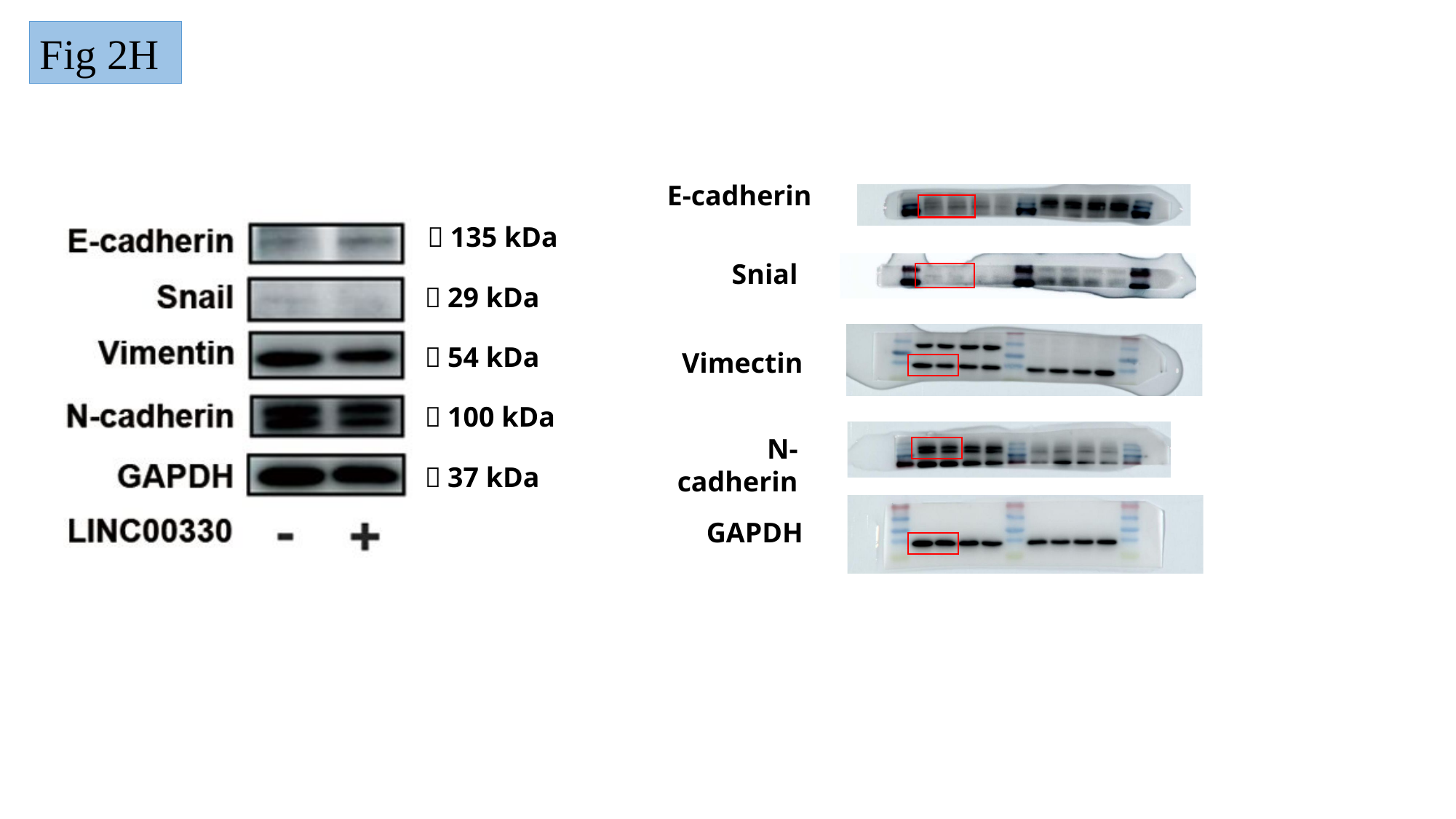

Fig 2H
E-cadherin
〜135 kDa
Snial
〜29 kDa
〜54 kDa
Vimectin
〜100 kDa
N-cadherin
〜37 kDa
GAPDH

## Slide 2
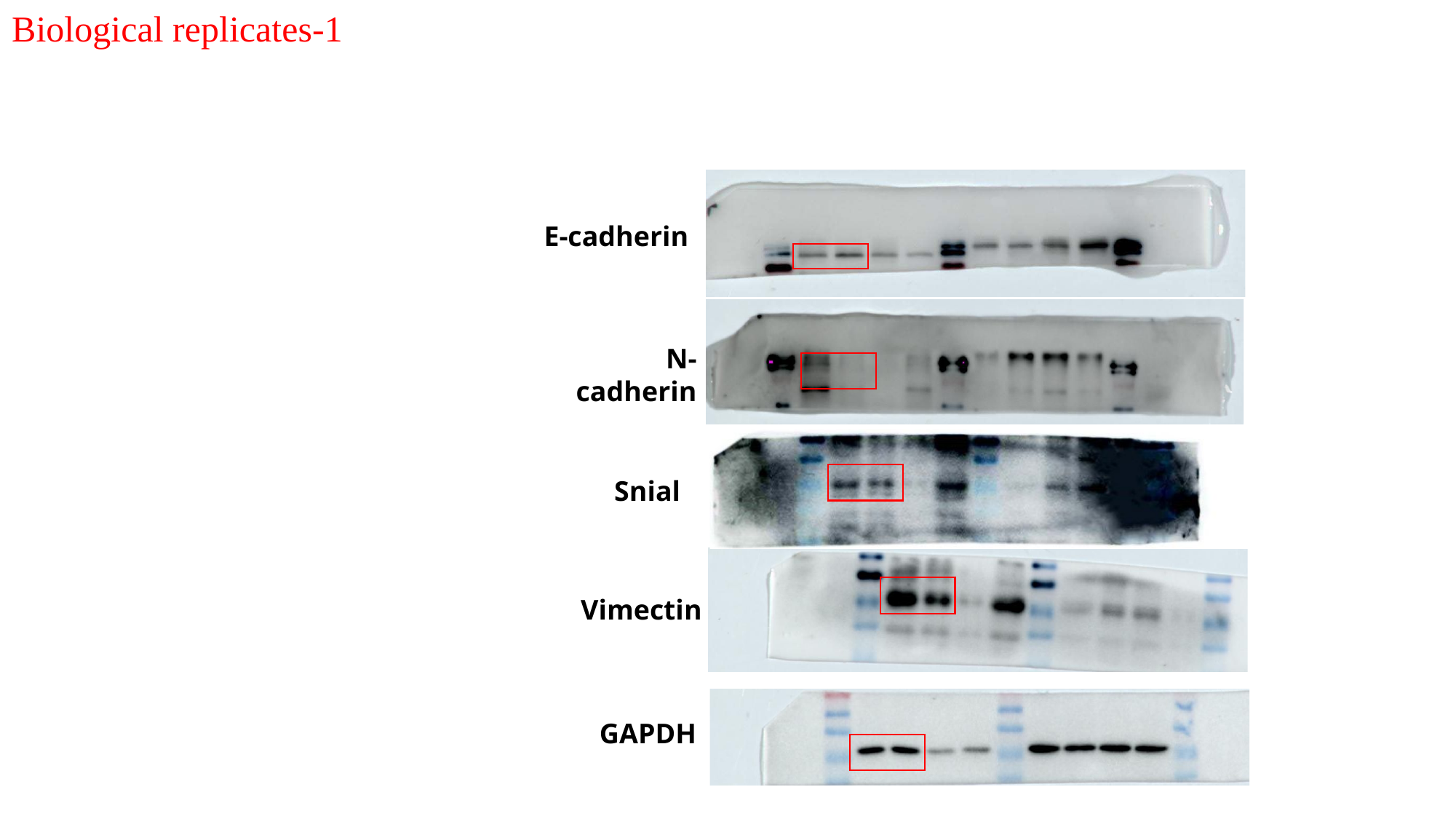

Biological replicates-1
E-cadherin
N-cadherin
Snial
Vimectin
GAPDH

## Slide 3
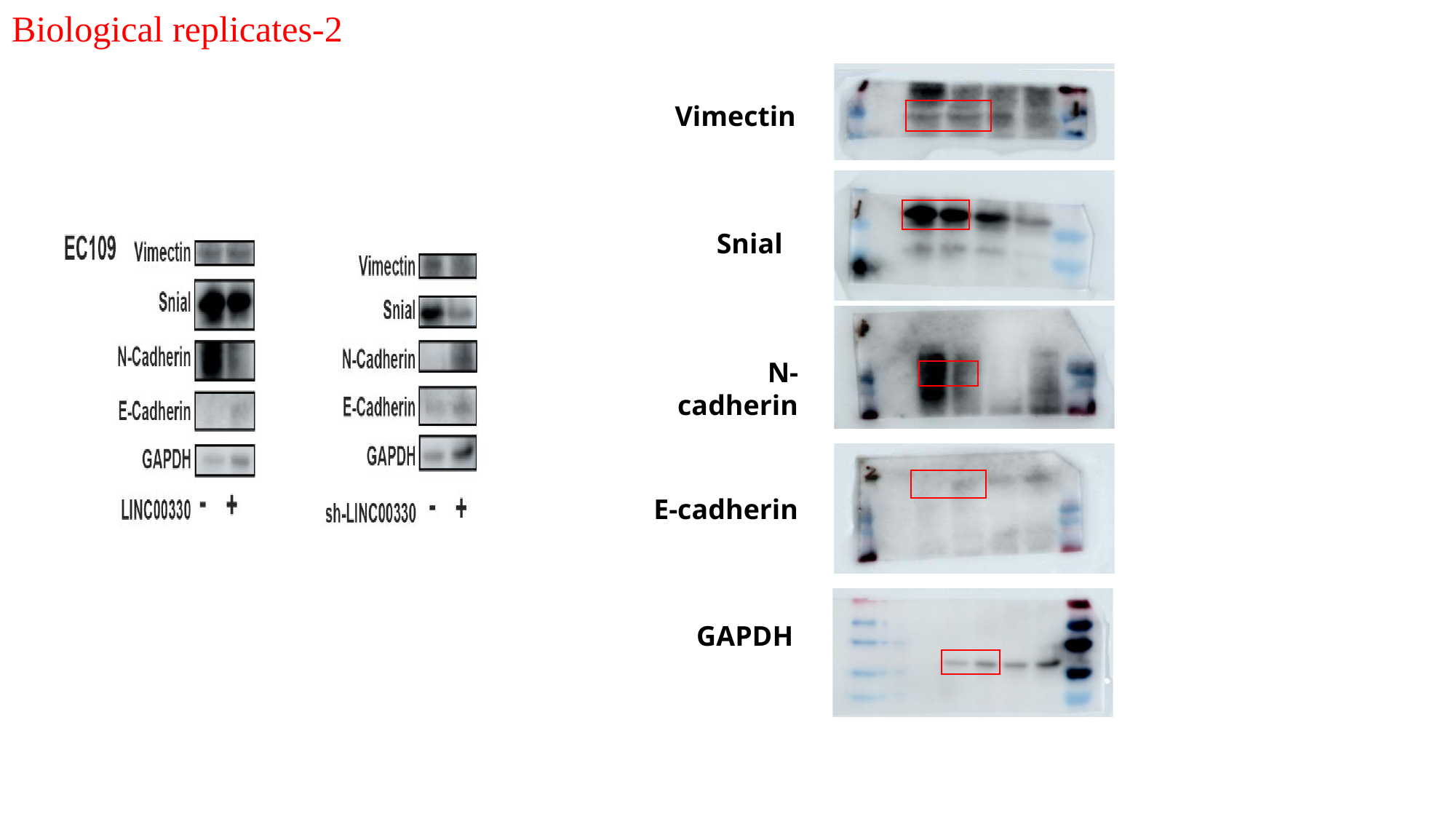

Biological replicates-2
Vimectin
Snial
N-cadherin
E-cadherin
GAPDH

## Slide 4
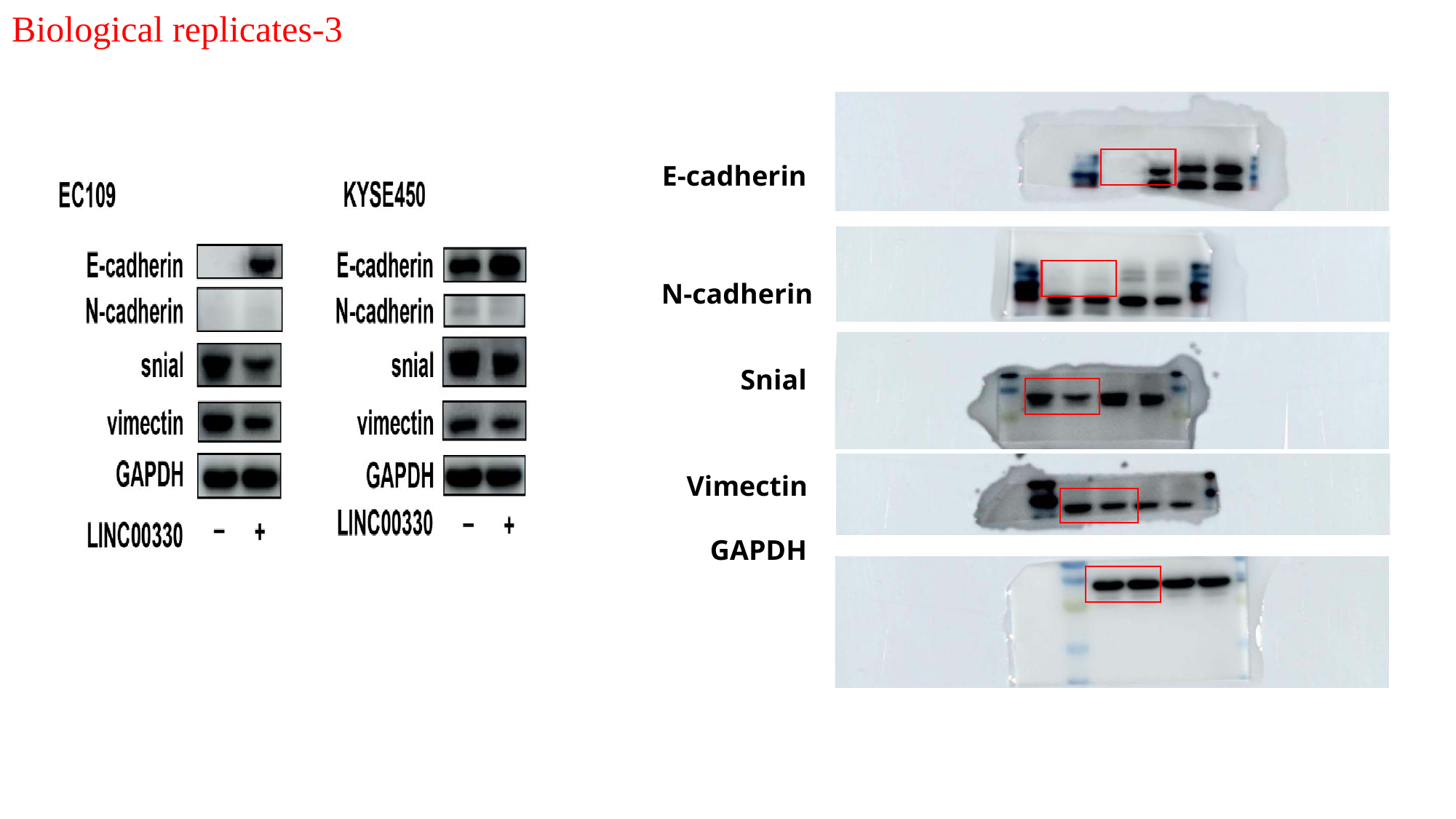

Biological replicates-3
E-cadherin
N-cadherin
Snial
Vimectin
GAPDH

## Slide 5
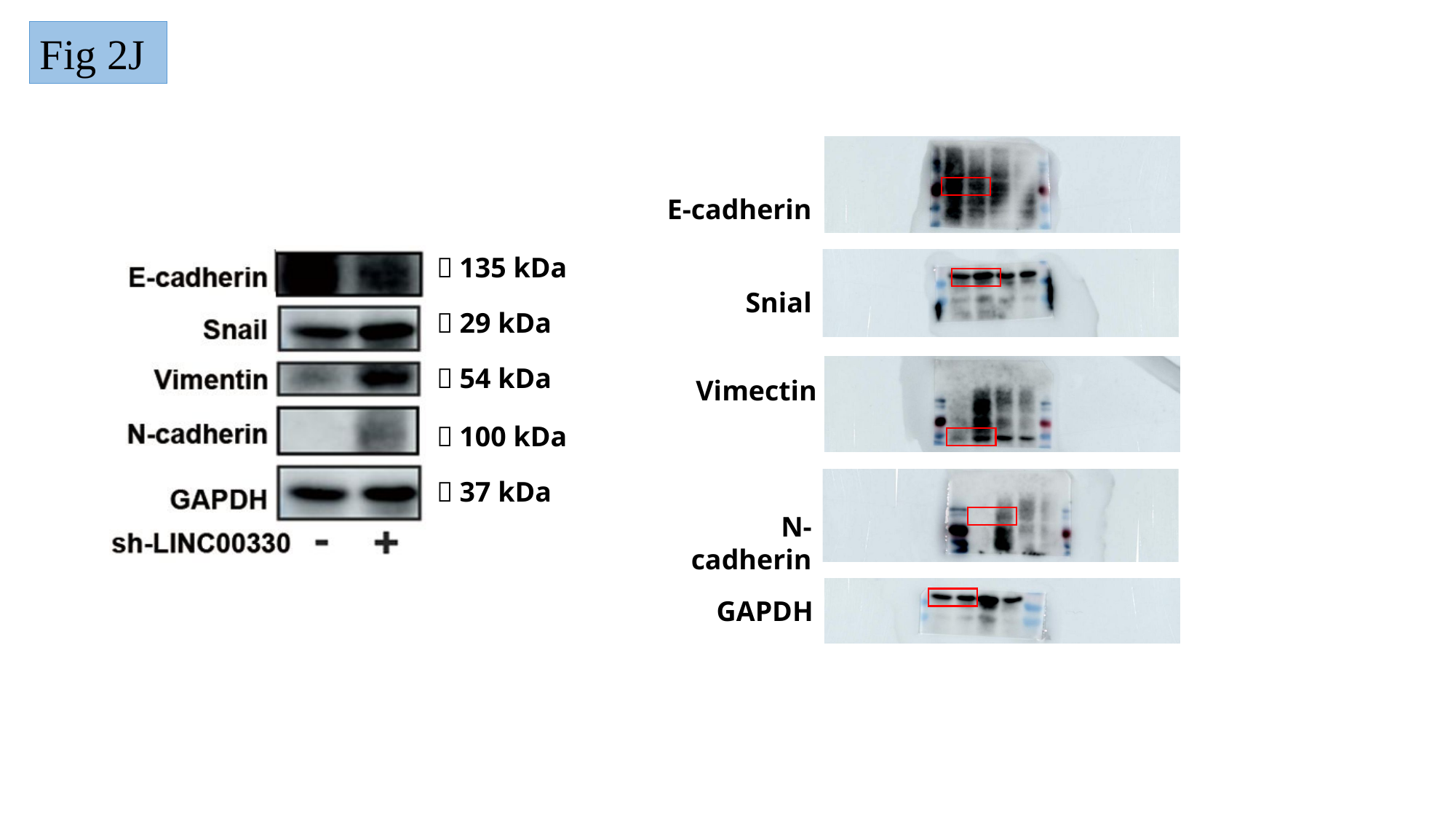

Fig 2J
E-cadherin
〜135 kDa
Snial
〜29 kDa
〜54 kDa
Vimectin
〜100 kDa
〜37 kDa
N-cadherin
GAPDH

## Slide 6
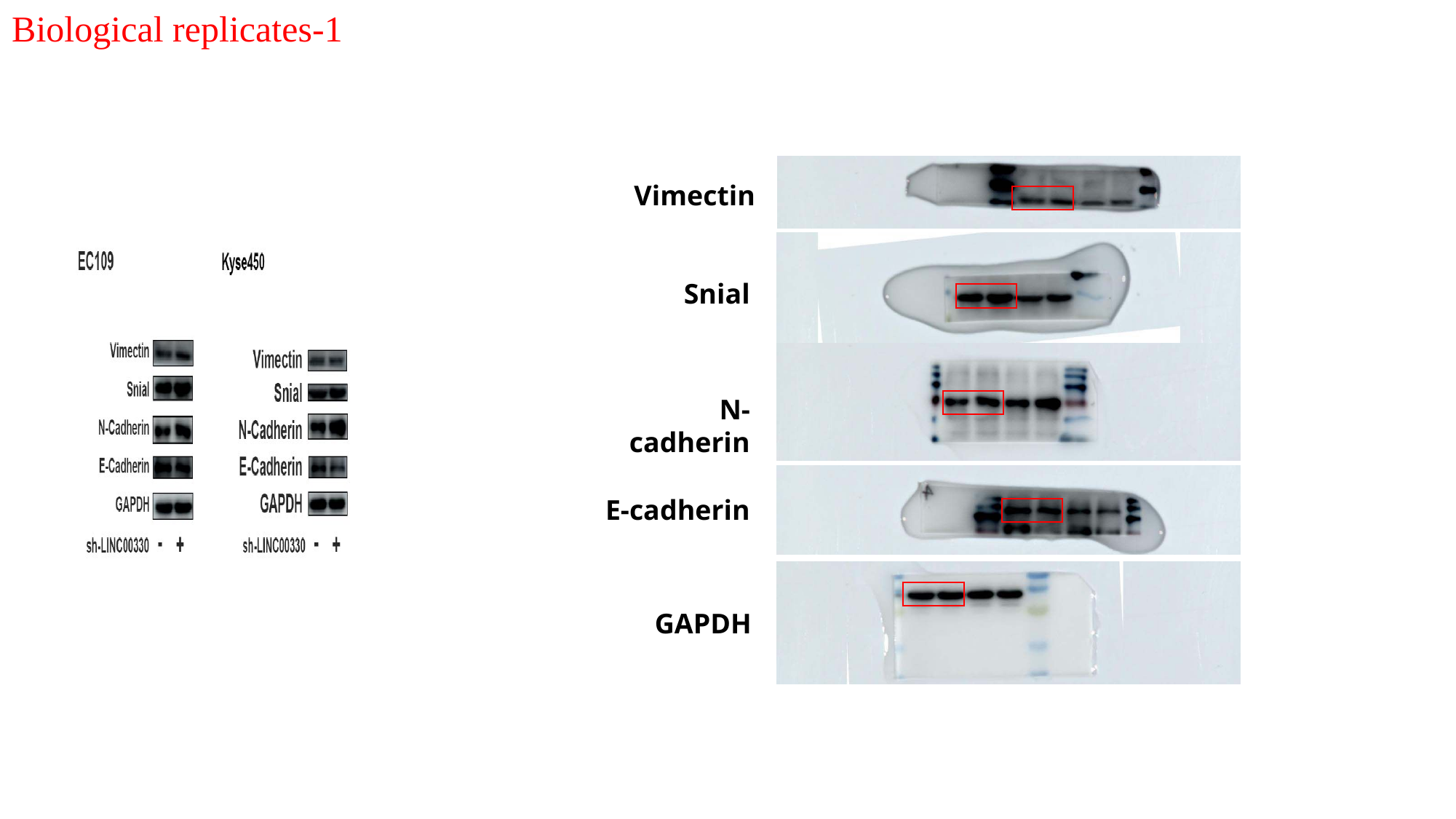

Biological replicates-1
Vimectin
Snial
N-cadherin
E-cadherin
GAPDH

## Slide 7
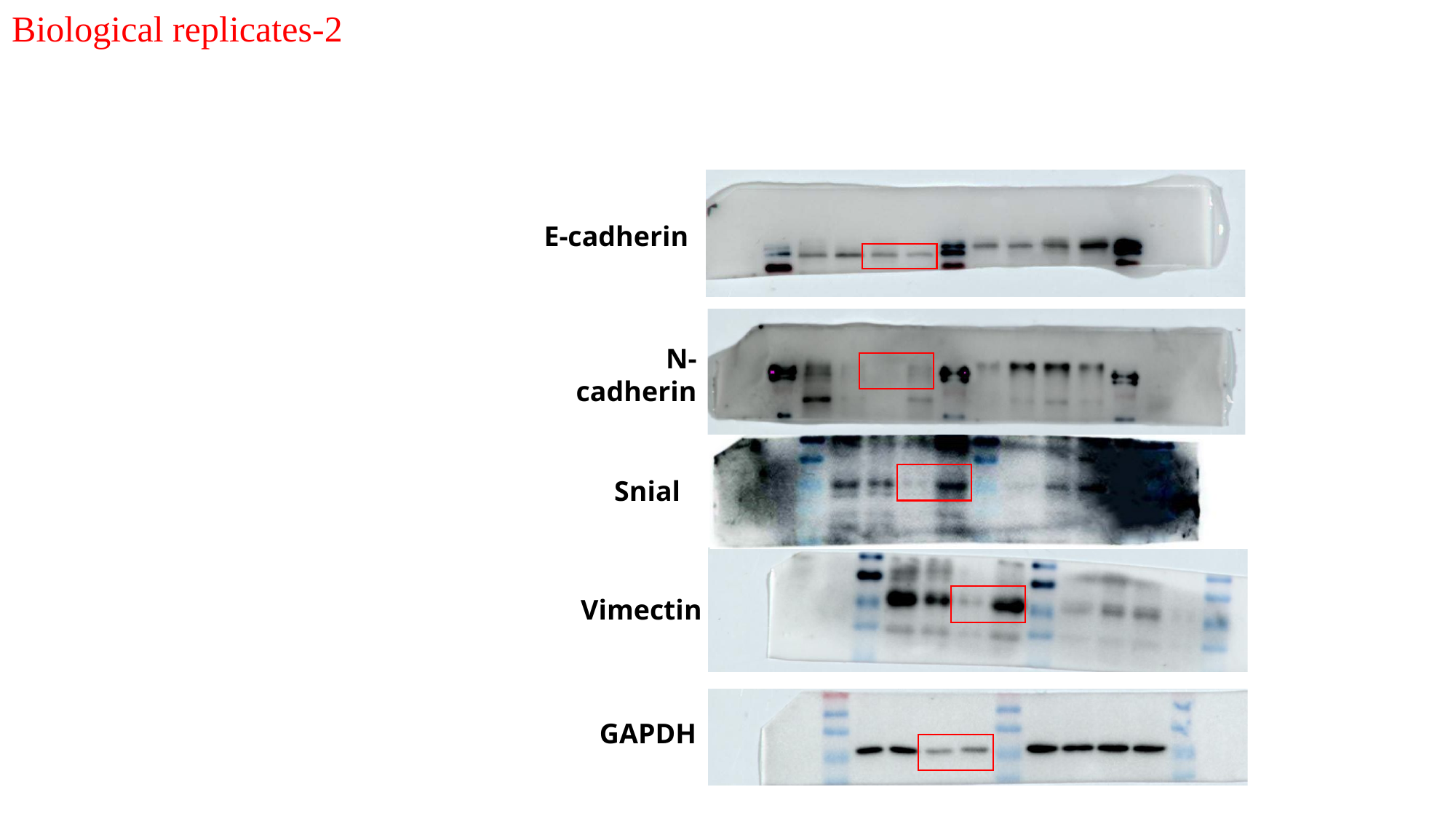

Biological replicates-2
E-cadherin
N-cadherin
Snial
Vimectin
GAPDH

## Slide 8
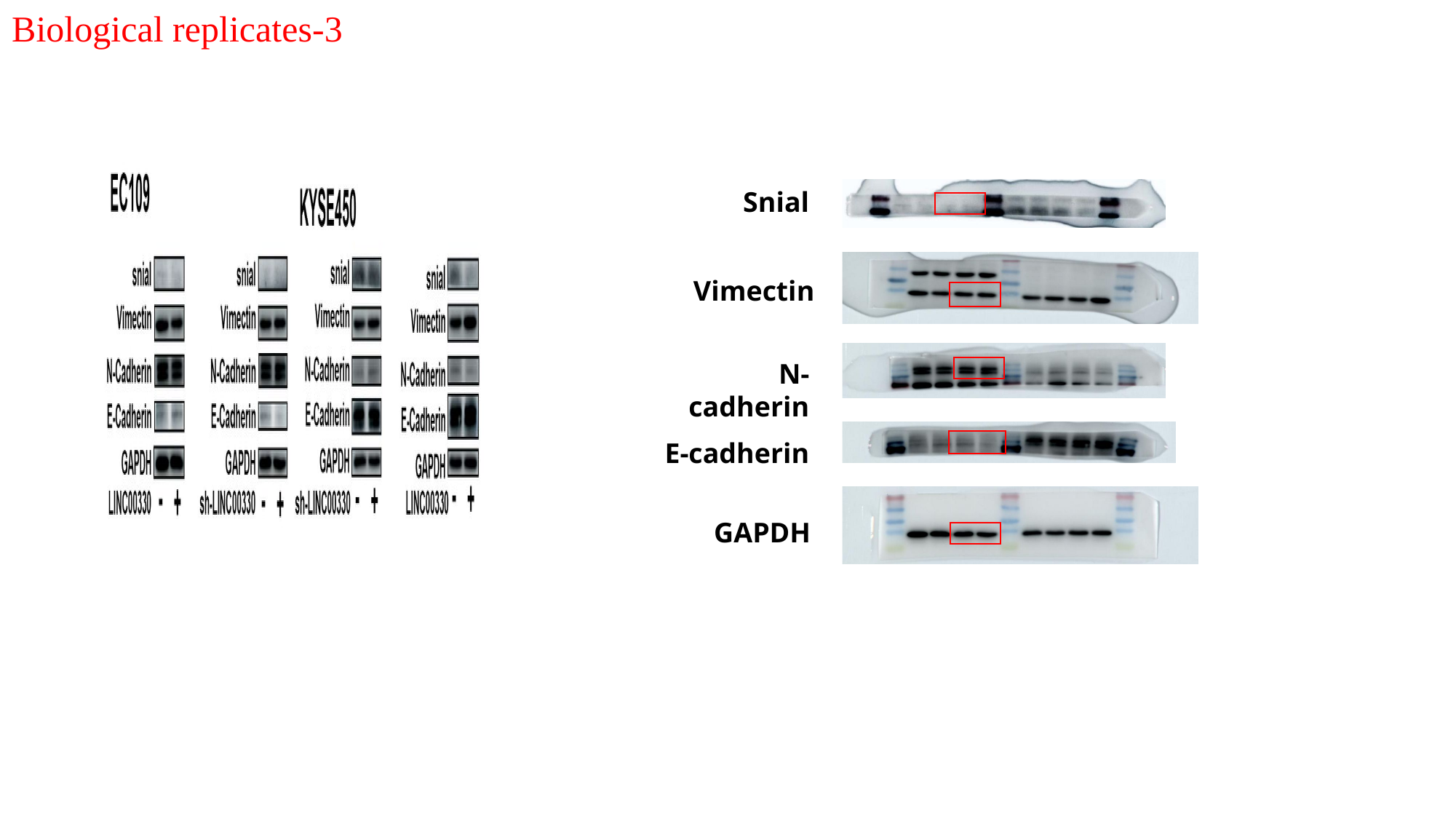

Biological replicates-3
Snial
Vimectin
N-cadherin
E-cadherin
GAPDH

## Slide 9
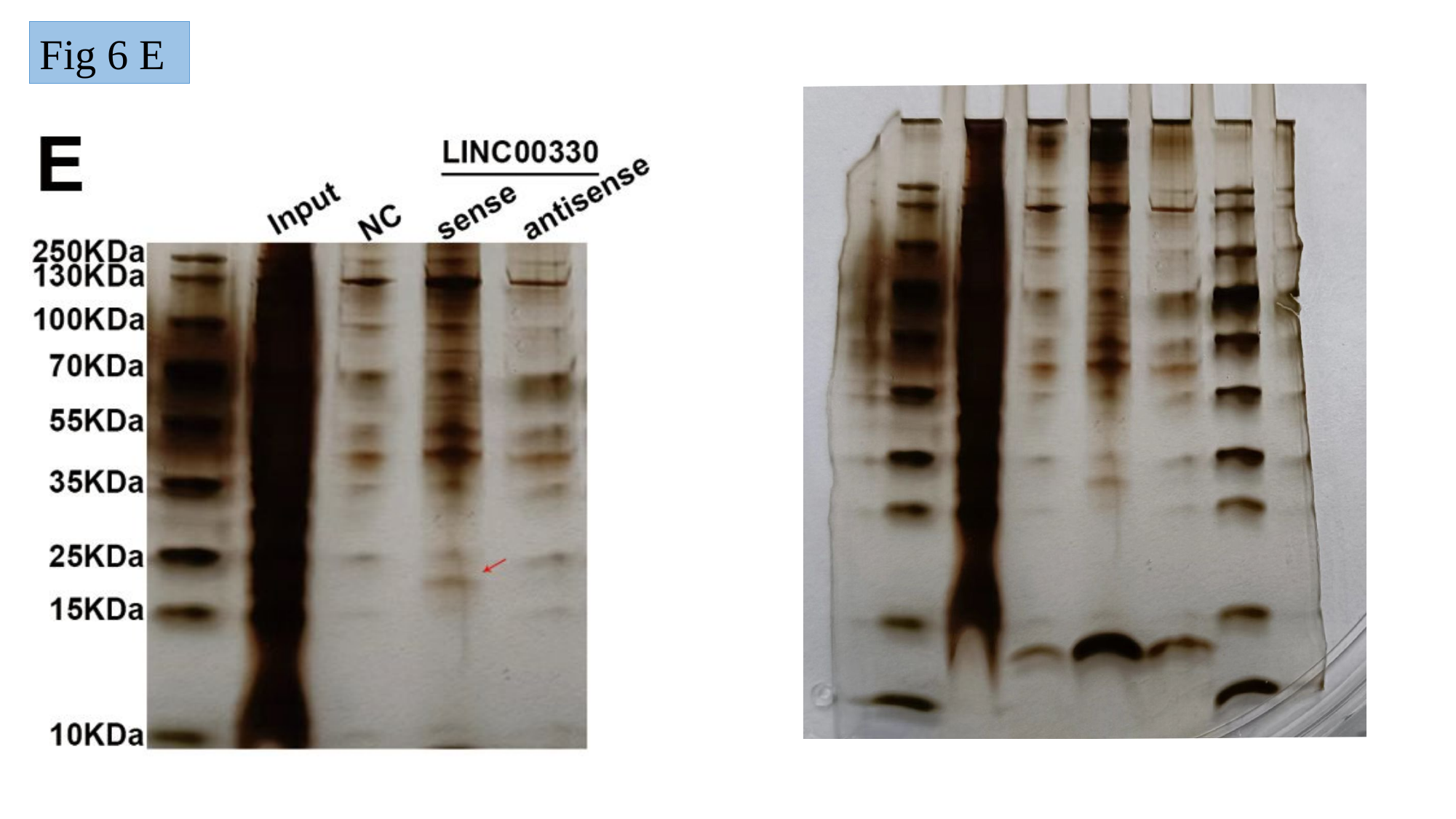

Fig 6 E

## Slide 10
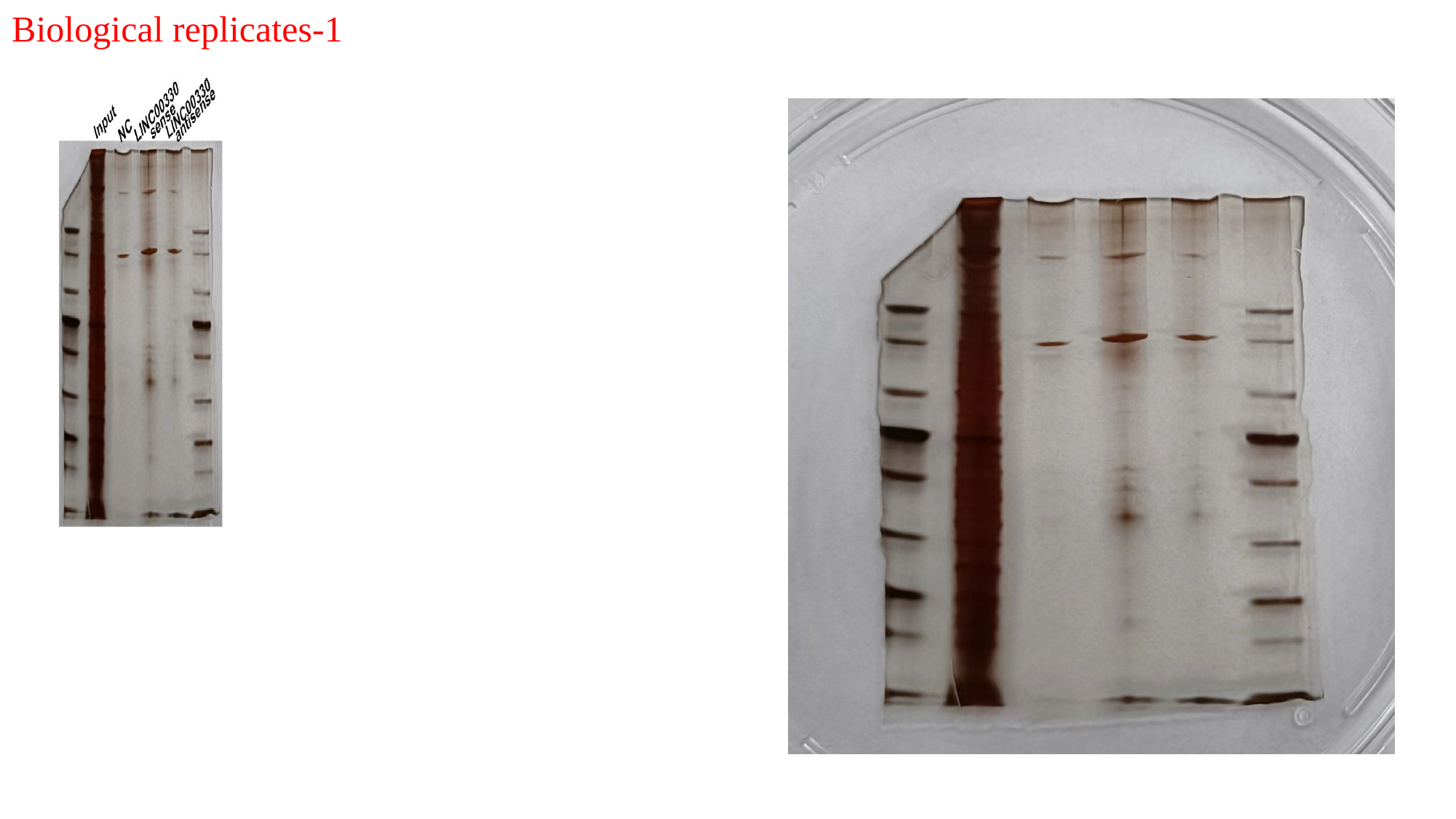

Biological replicates-1

## Slide 11
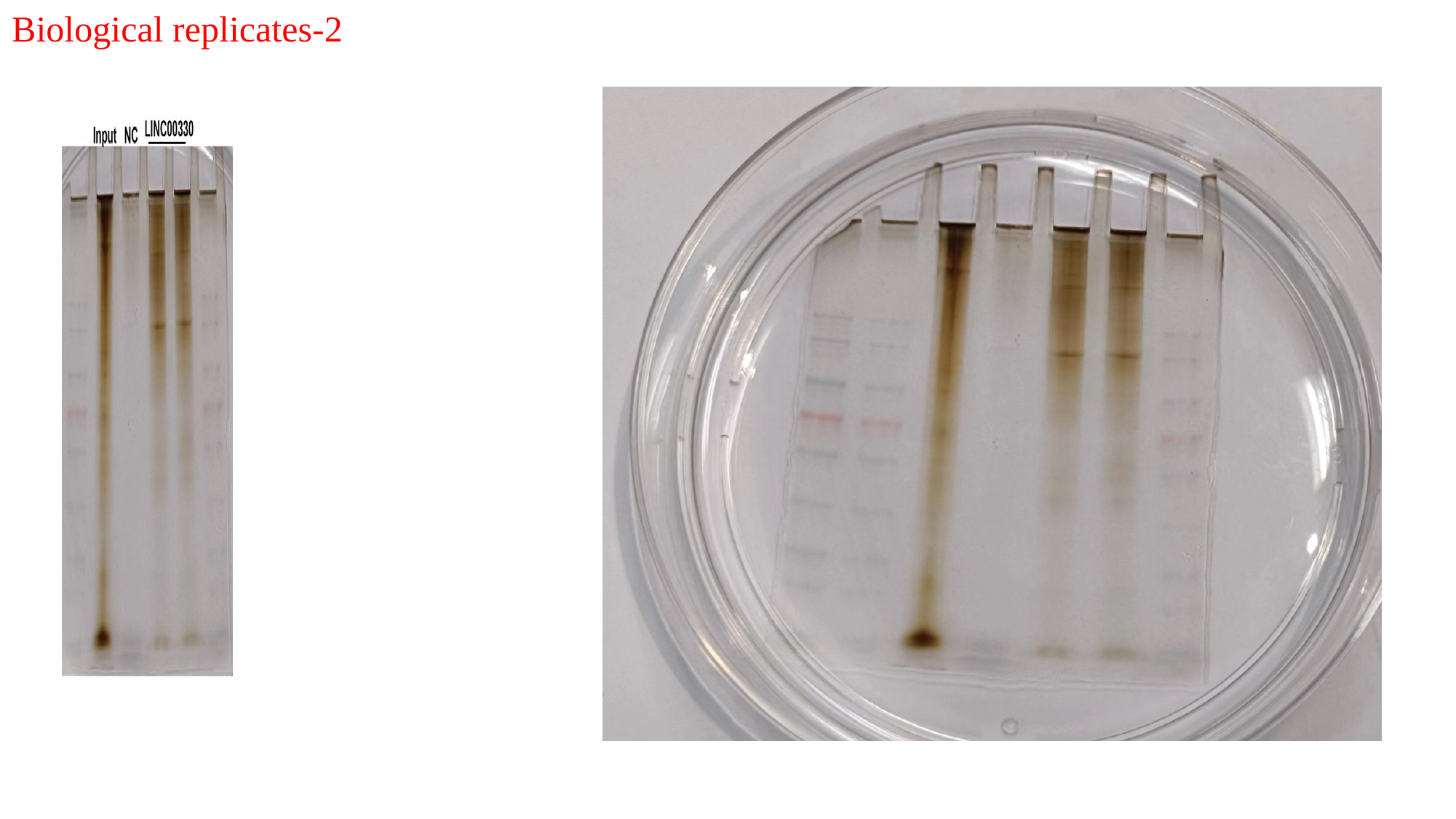

Biological replicates-2

## Slide 12
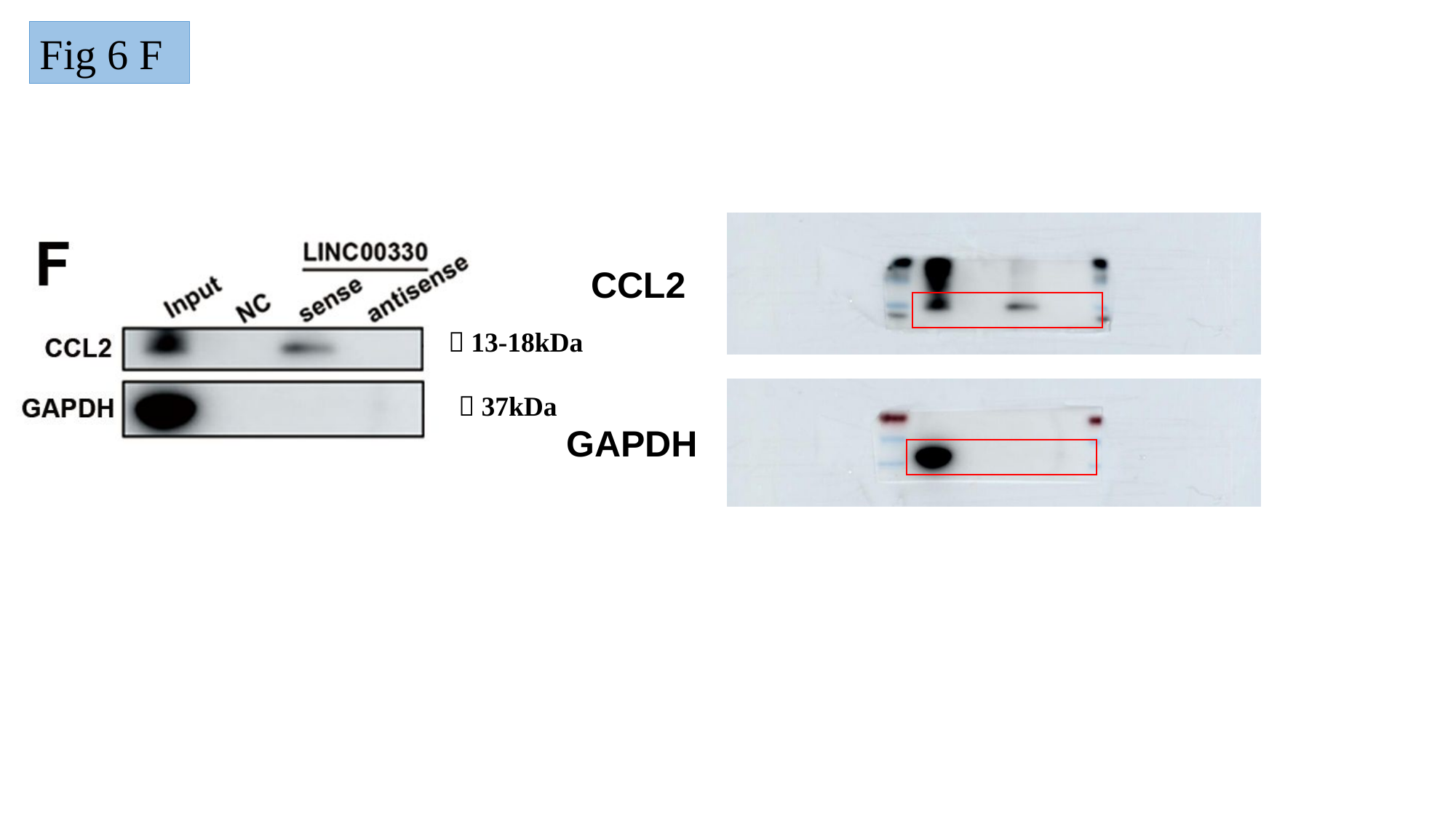

Fig 6 F
CCL2
〜13-18kDa
〜37kDa
GAPDH

## Slide 13
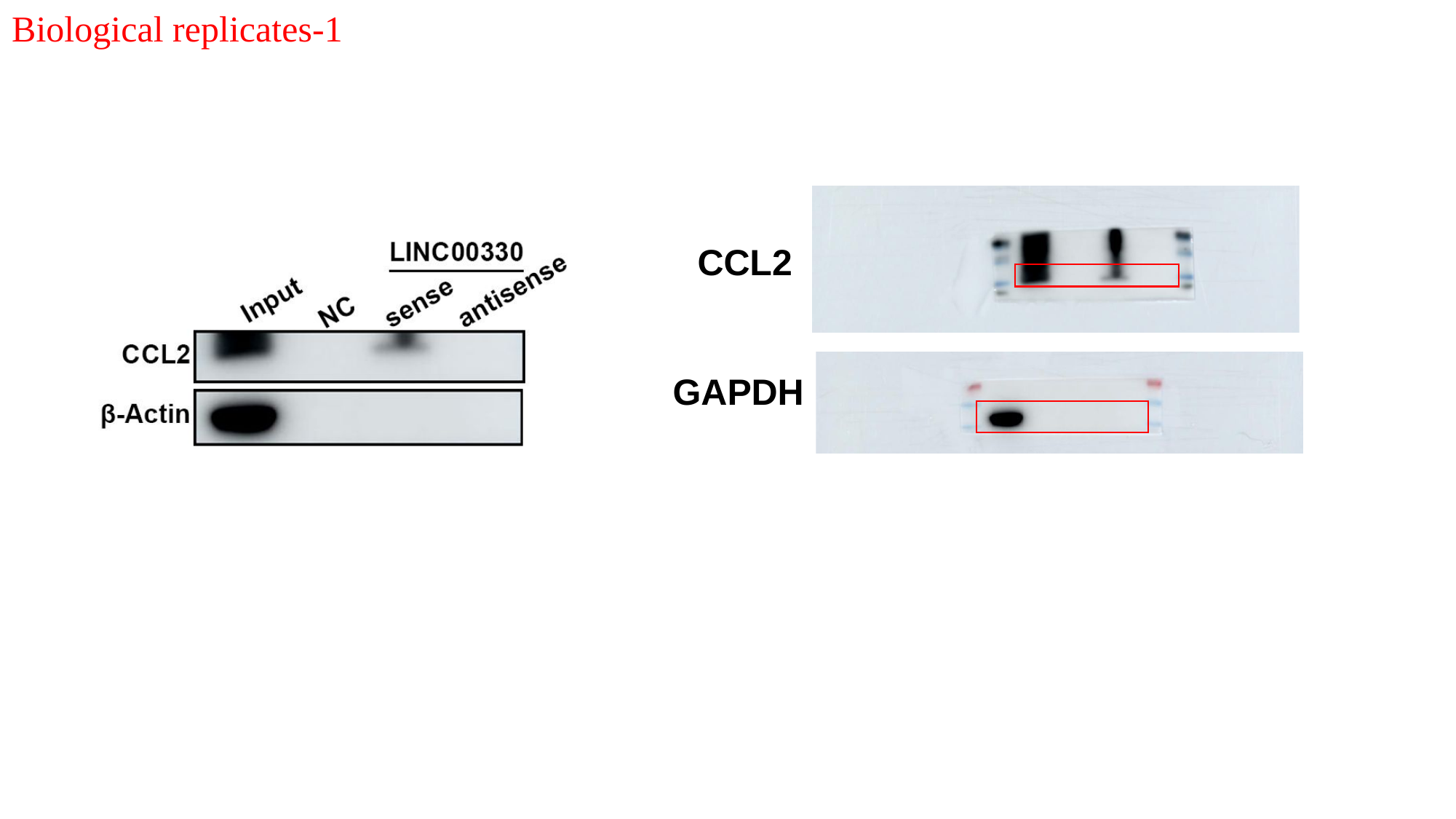

Biological replicates-1
CCL2
GAPDH

## Slide 14
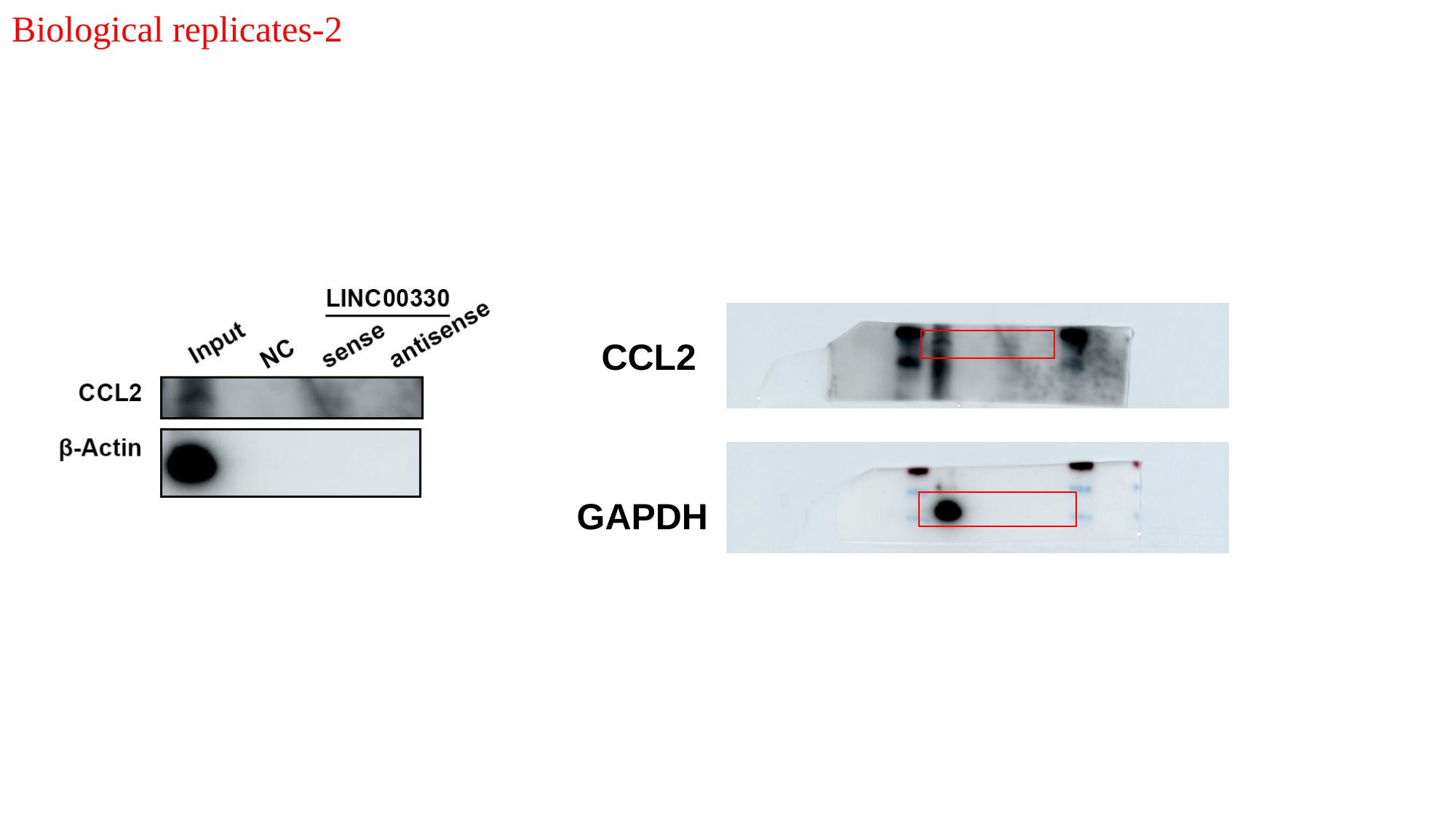

Biological replicates-2
CCL2
GAPDH

## Slide 15
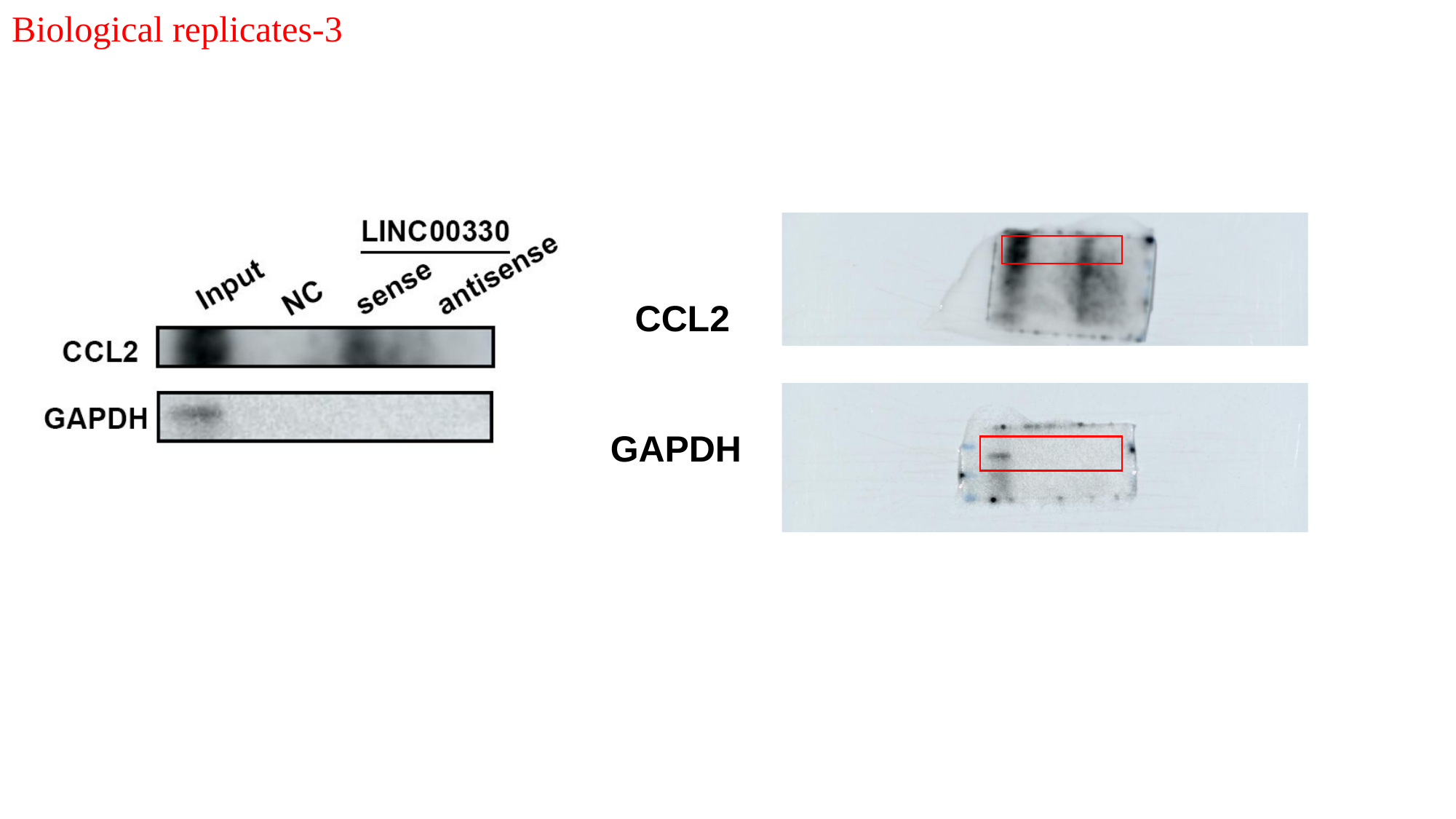

Biological replicates-3
CCL2
GAPDH

## Slide 16
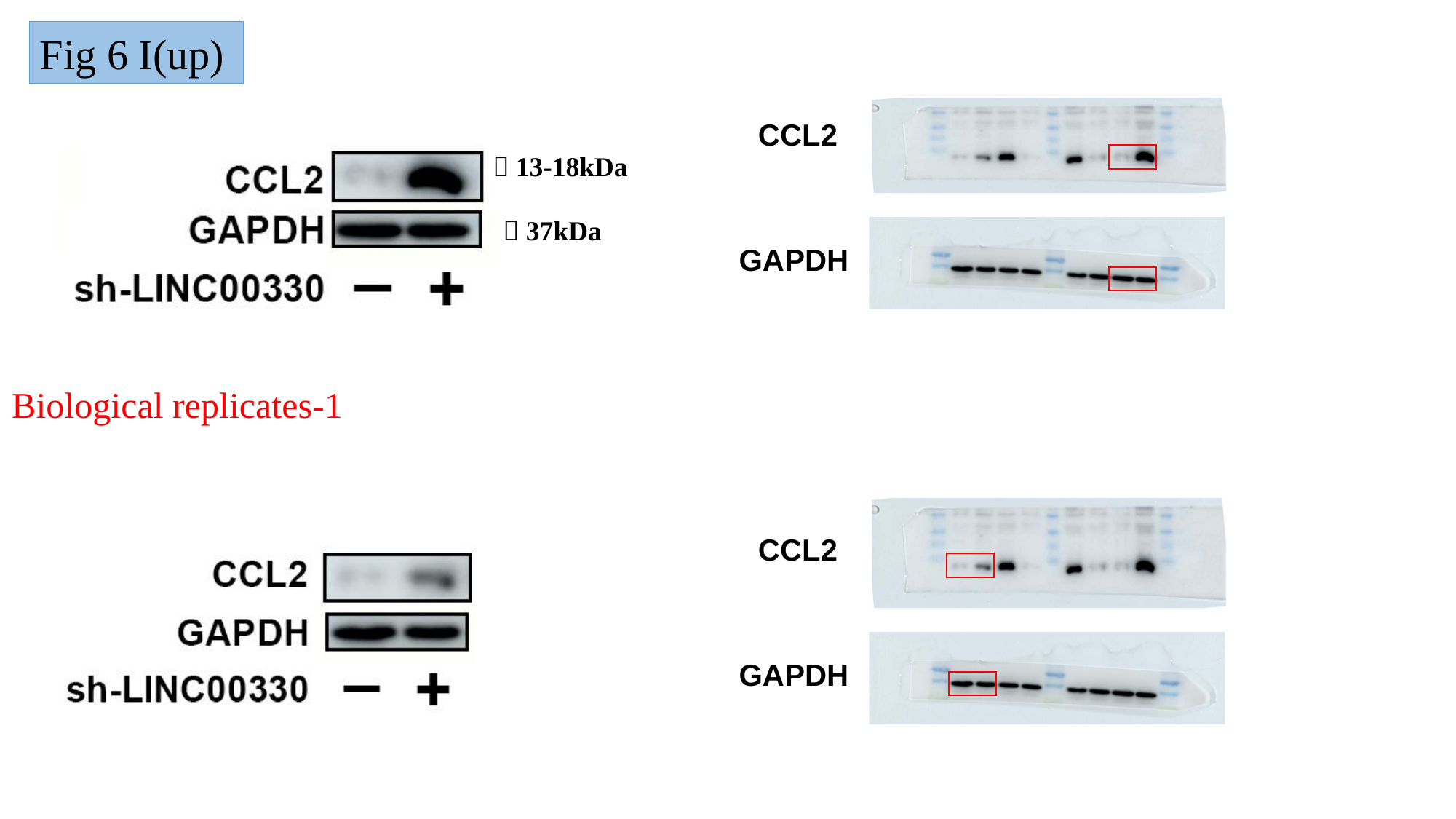

Fig 6 I(up)
CCL2
〜13-18kDa
〜37kDa
GAPDH
Biological replicates-1
CCL2
GAPDH

## Slide 17
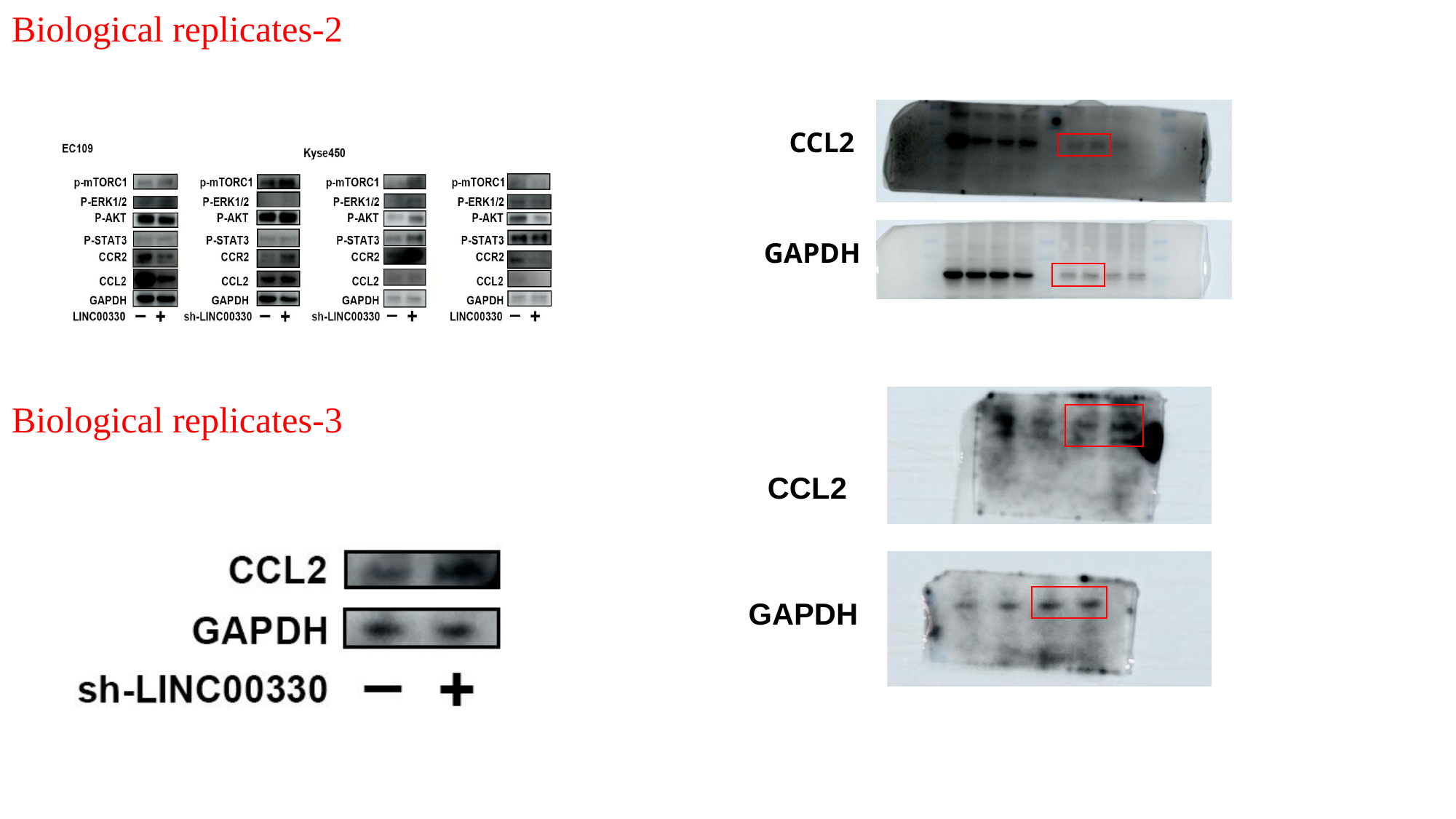

Biological replicates-2
CCL2
GAPDH
Biological replicates-3
CCL2
GAPDH

## Slide 18
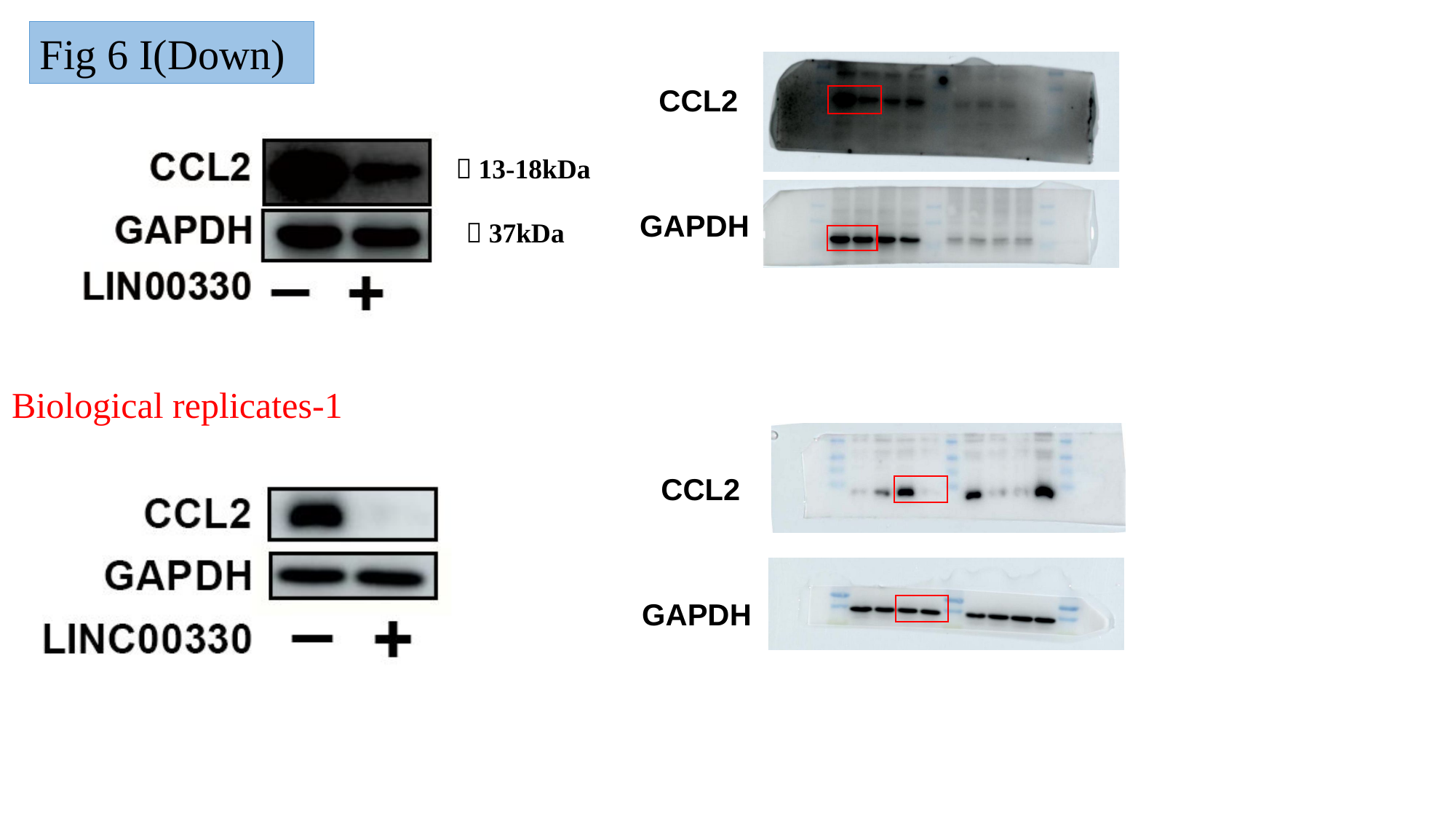

Fig 6 I(Down)
CCL2
〜13-18kDa
GAPDH
〜37kDa
Biological replicates-1
CCL2
GAPDH

## Slide 19
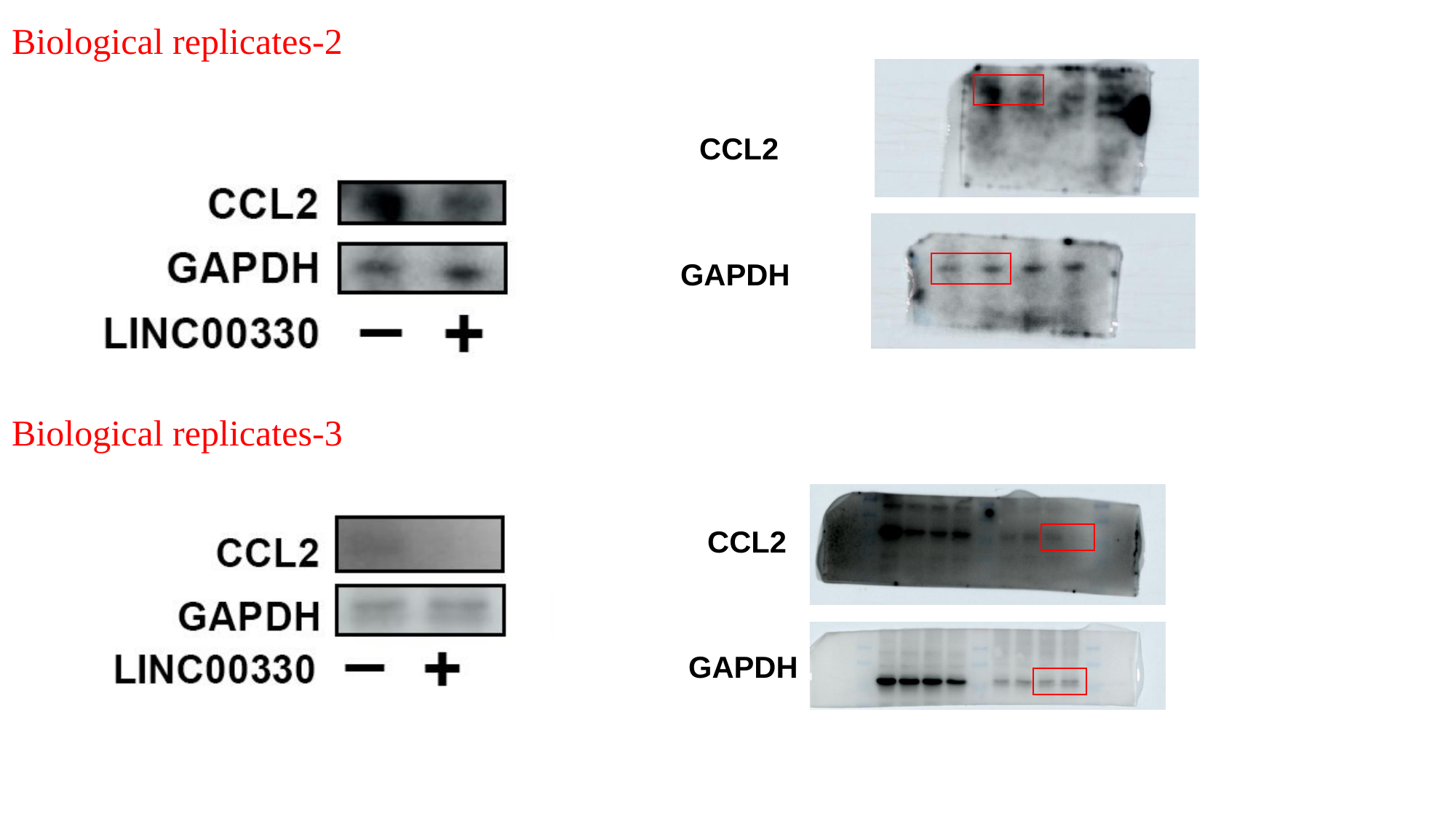

Biological replicates-2
CCL2
GAPDH
Biological replicates-3
CCL2
GAPDH

## Slide 20
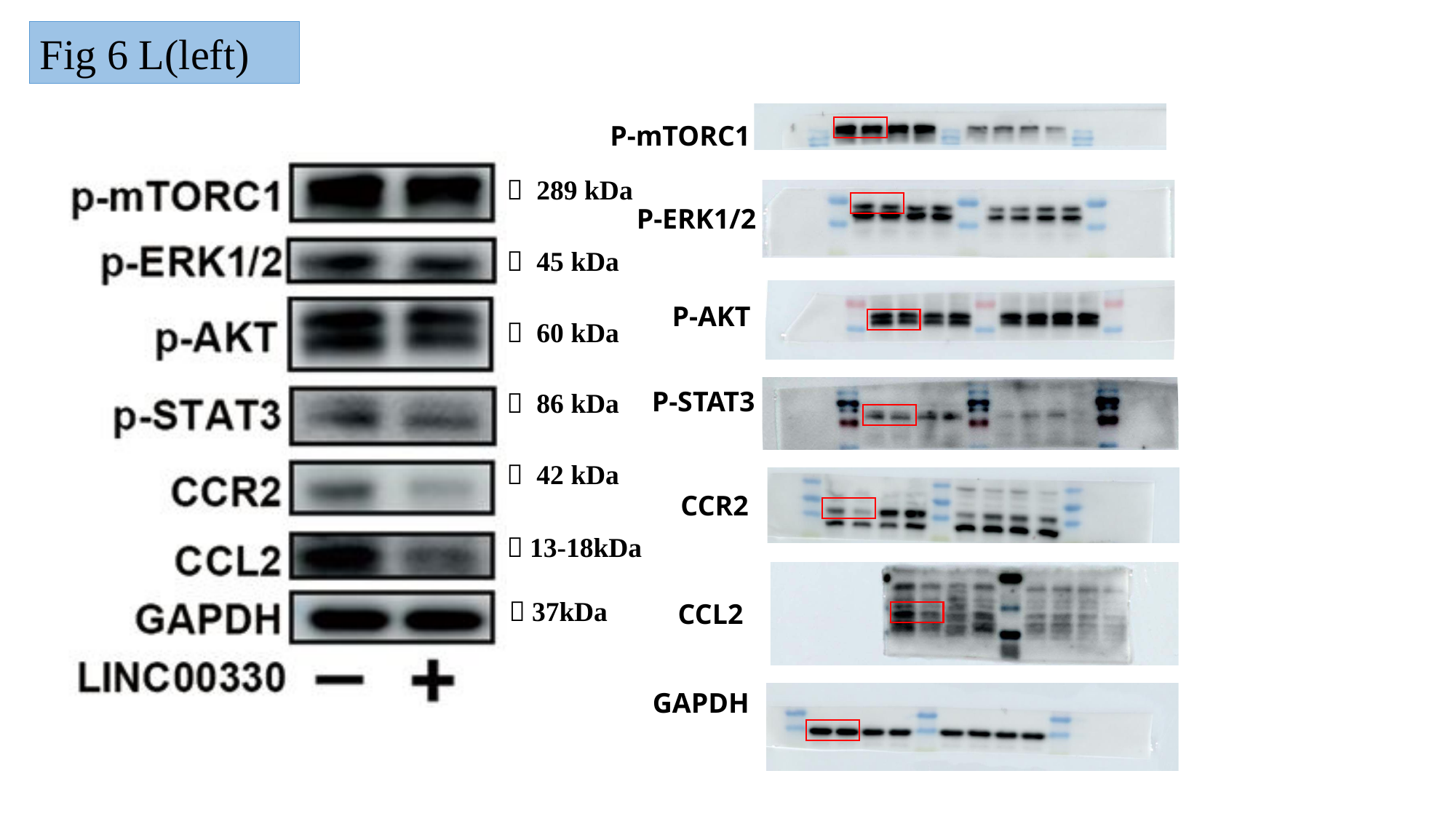

Fig 6 L(left)
P-mTORC1
〜 289 kDa
P-ERK1/2
〜 45 kDa
P-AKT
〜 60 kDa
P-STAT3
〜 86 kDa
〜 42 kDa
CCR2
〜13-18kDa
〜37kDa
CCL2
GAPDH

## Slide 21
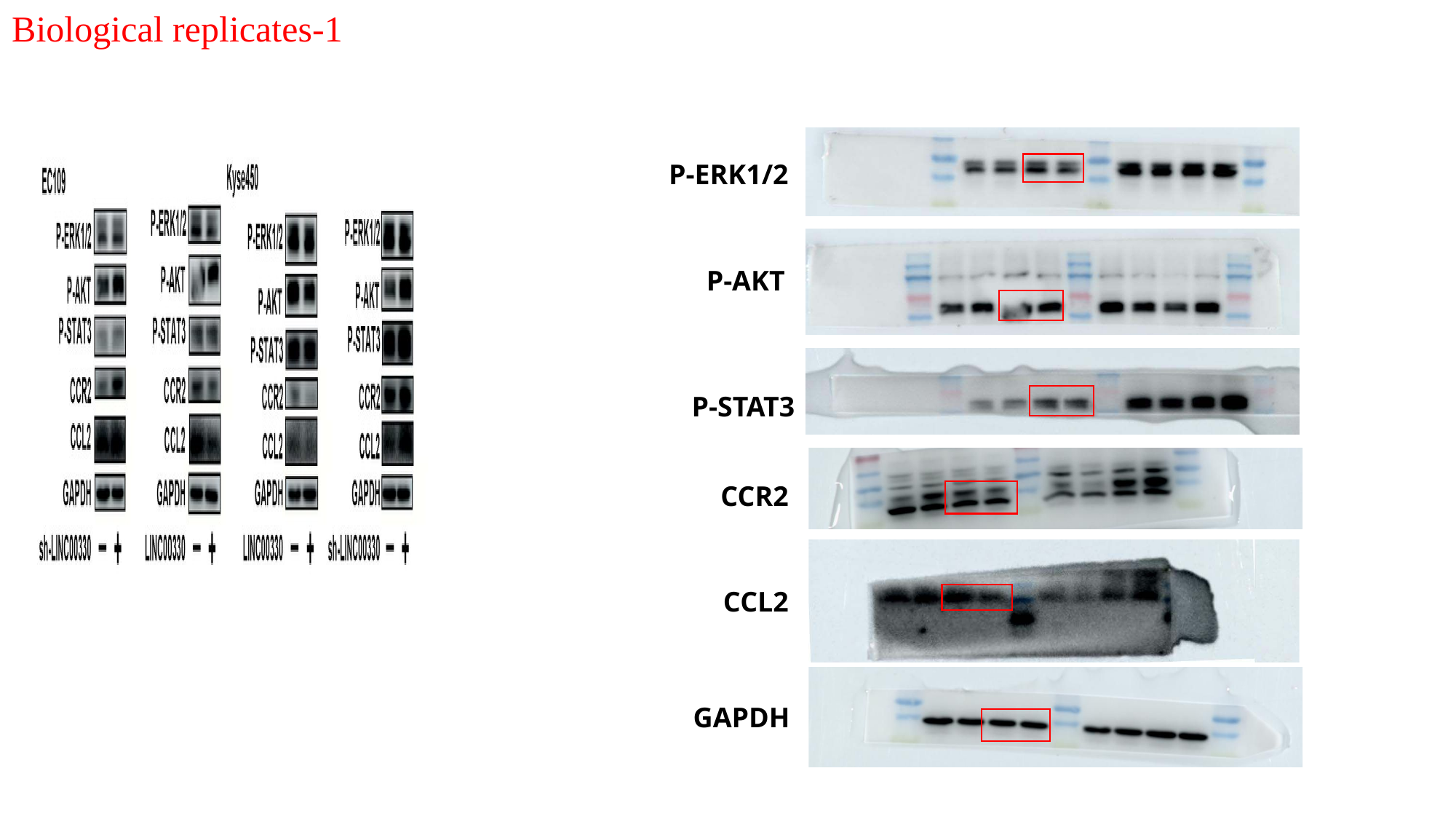

Biological replicates-1
P-ERK1/2
P-AKT
P-STAT3
CCR2
CCL2
GAPDH

## Slide 22
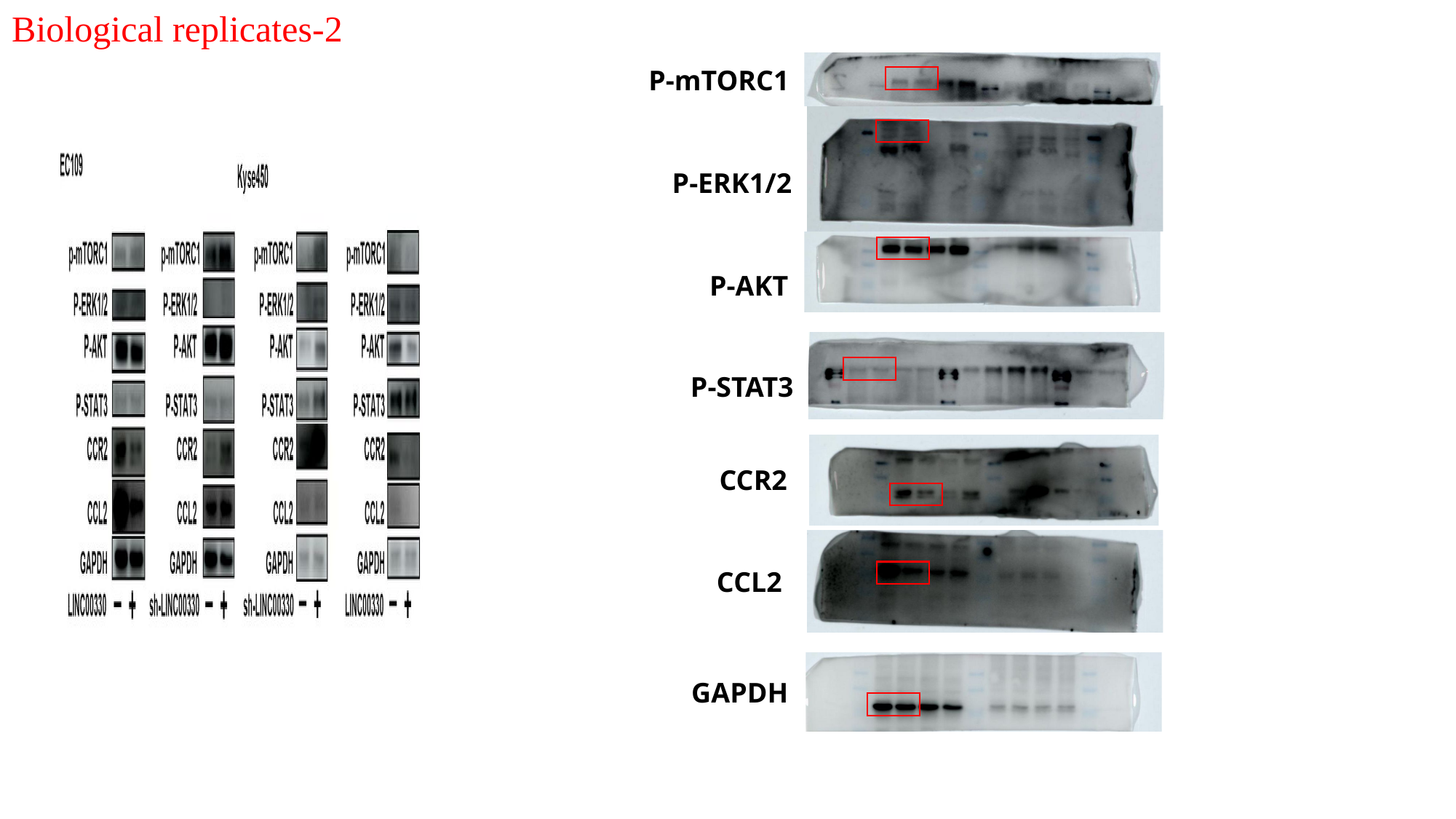

Biological replicates-2
P-mTORC1
P-ERK1/2
P-AKT
P-STAT3
CCR2
CCL2
GAPDH

## Slide 23
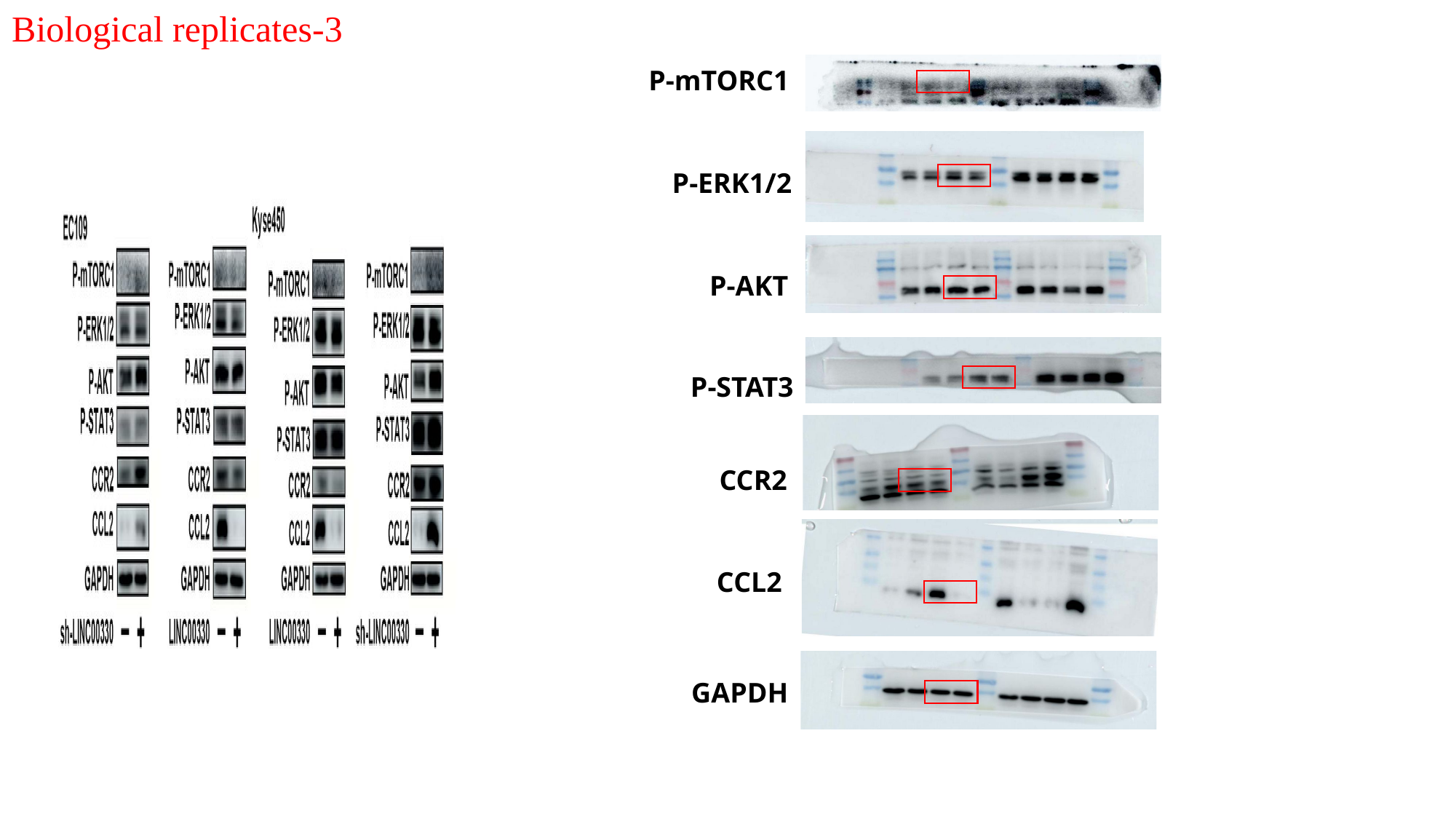

Biological replicates-3
P-mTORC1
P-ERK1/2
P-AKT
P-STAT3
CCR2
CCL2
GAPDH

## Slide 24
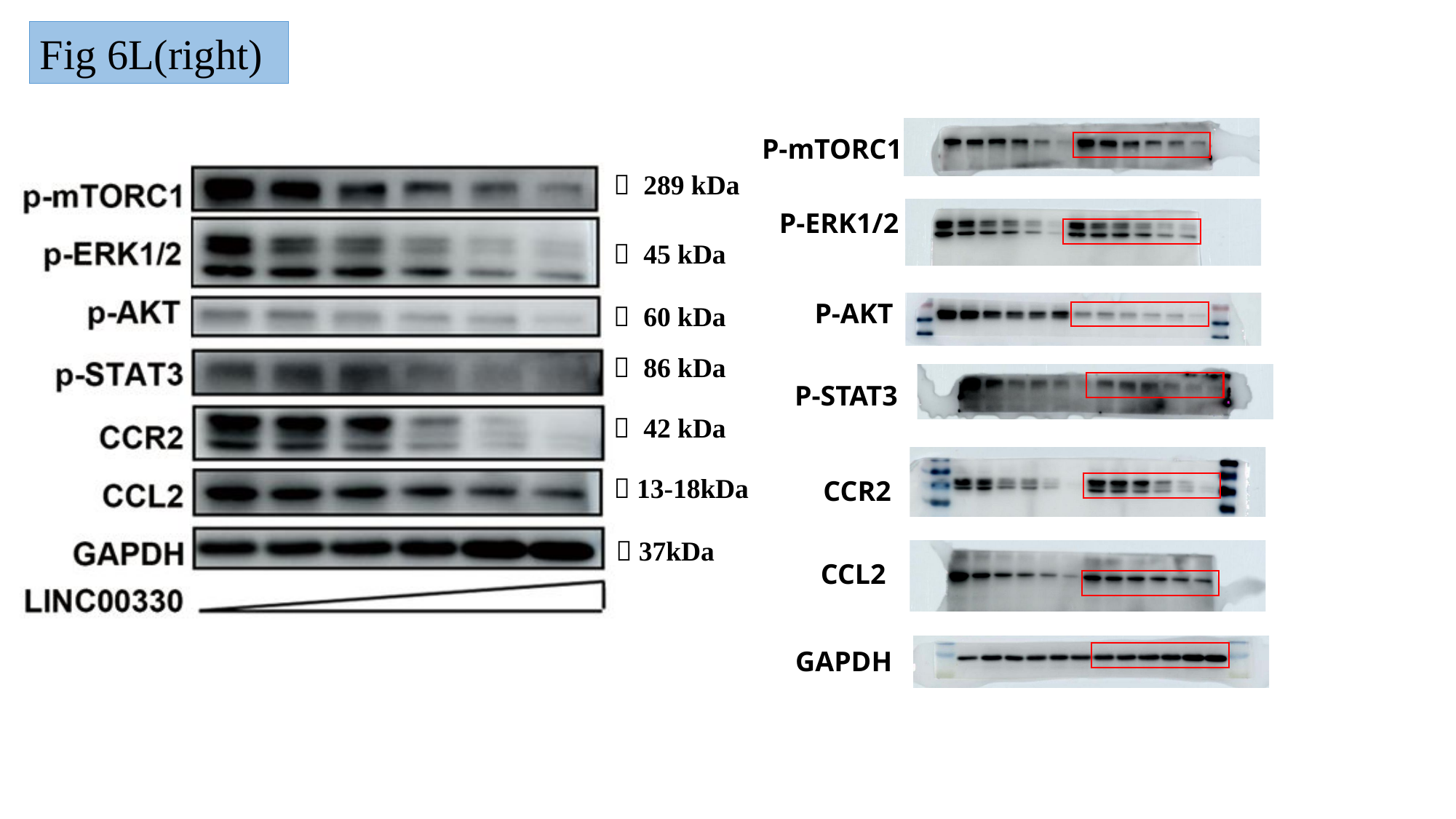

Fig 6L(right)
P-mTORC1
〜 289 kDa
P-ERK1/2
〜 45 kDa
P-AKT
〜 60 kDa
〜 86 kDa
P-STAT3
〜 42 kDa
〜13-18kDa
CCR2
〜37kDa
CCL2
GAPDH

## Slide 25
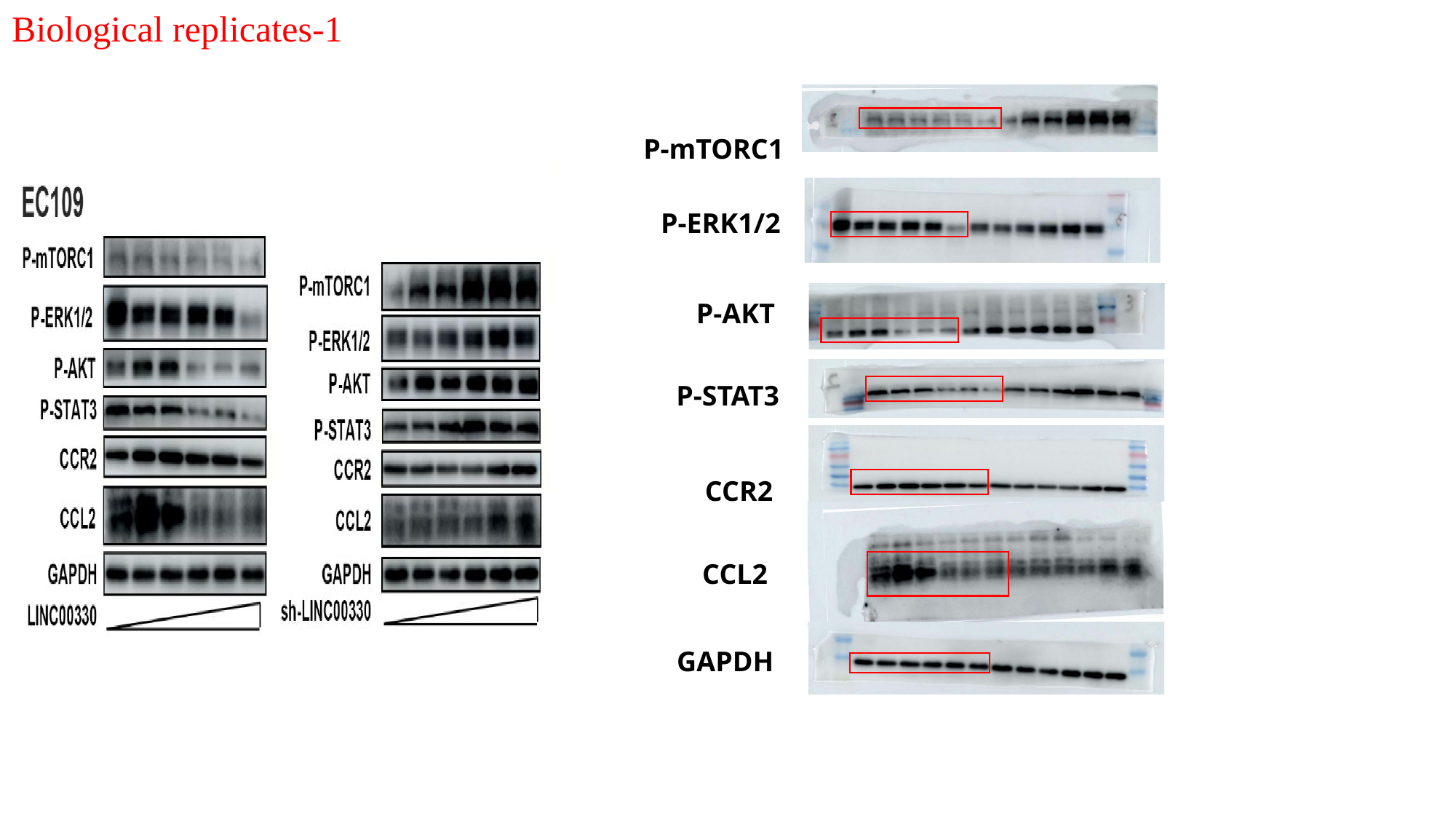

Biological replicates-1
P-mTORC1
P-ERK1/2
P-AKT
P-STAT3
CCR2
CCL2
GAPDH

## Slide 26
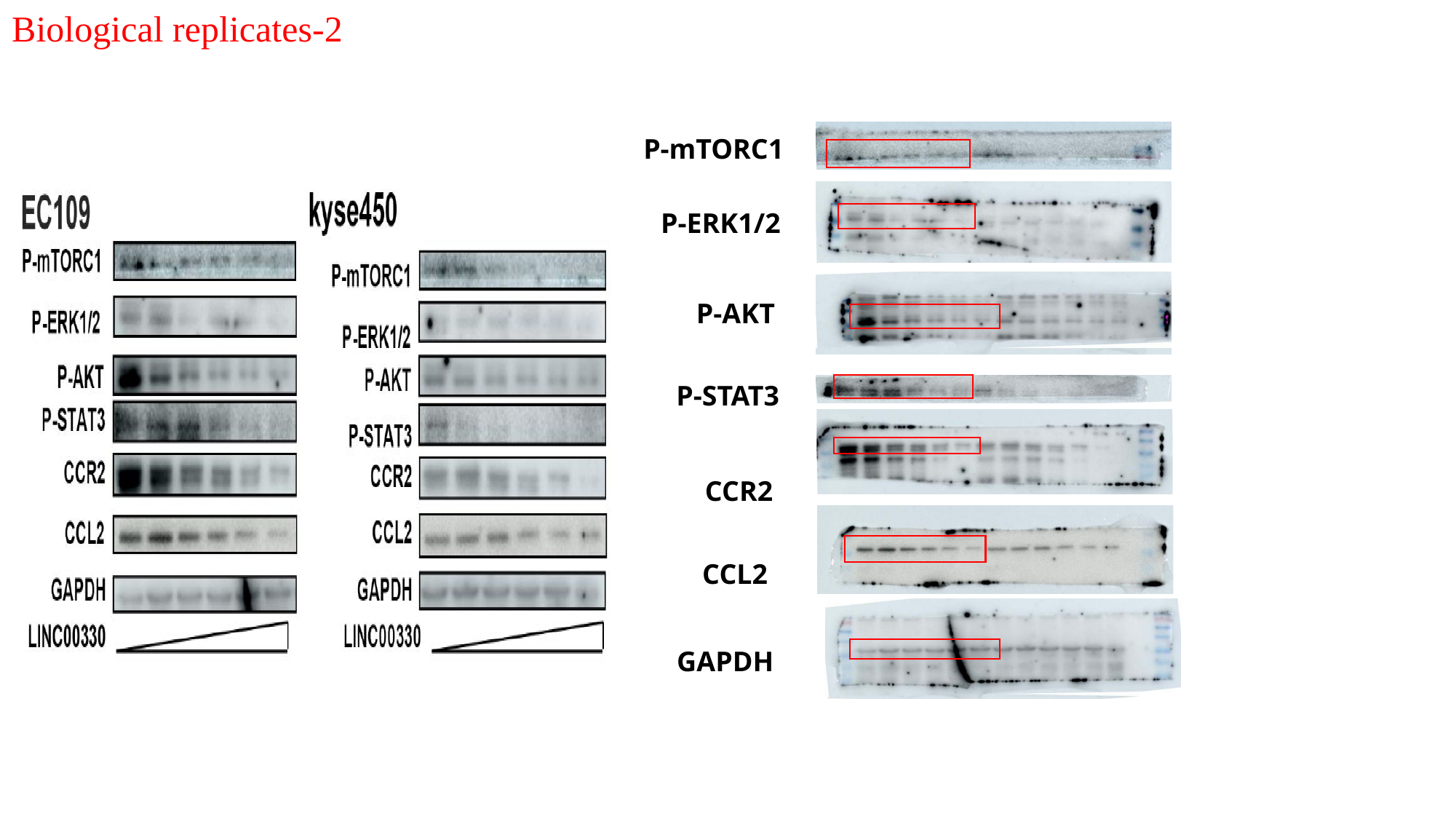

Biological replicates-2
P-mTORC1
P-ERK1/2
P-AKT
P-STAT3
CCR2
CCL2
GAPDH

## Slide 27
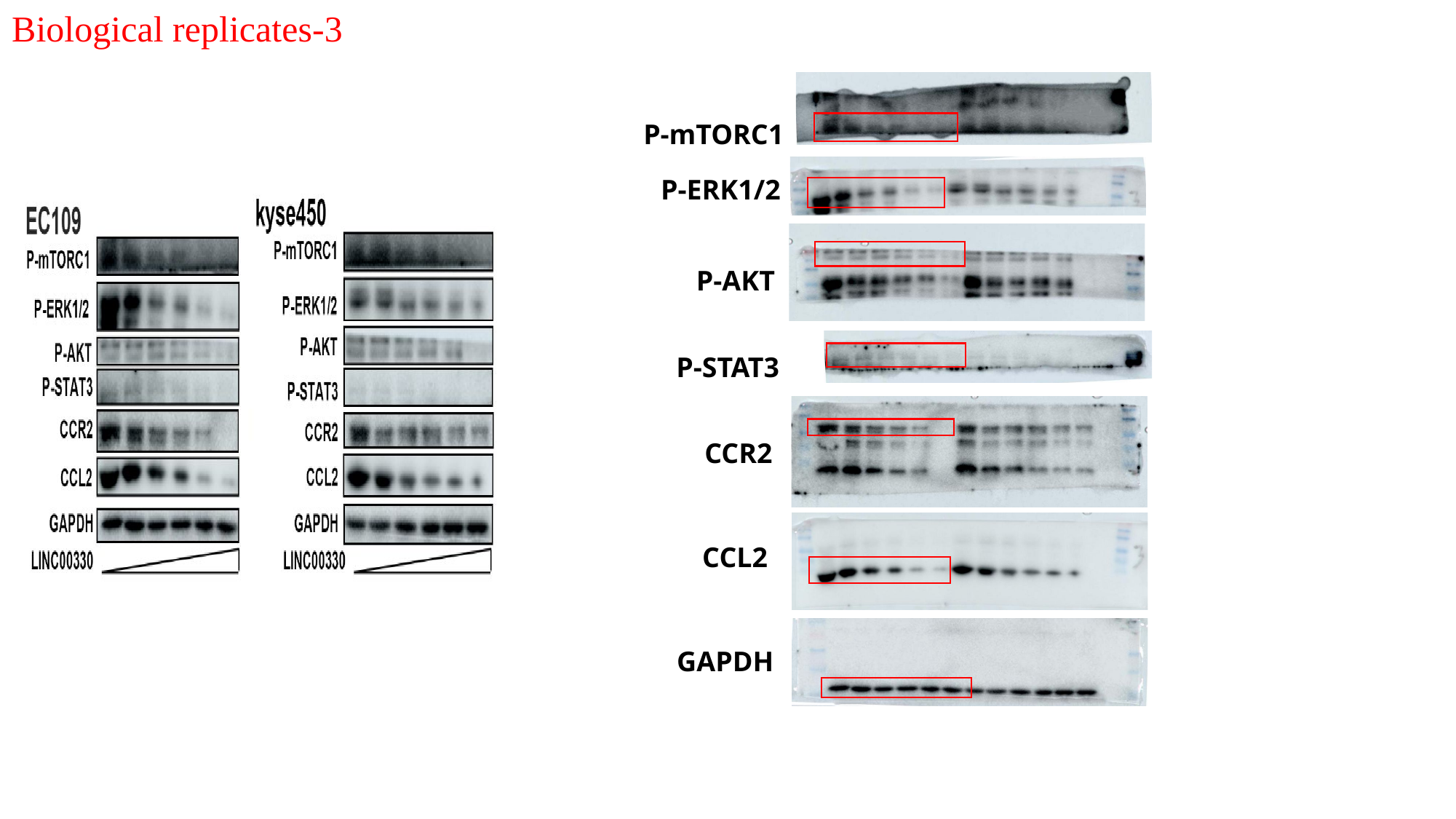

Biological replicates-3
P-mTORC1
P-ERK1/2
P-AKT
P-STAT3
CCR2
CCL2
GAPDH

## Slide 28
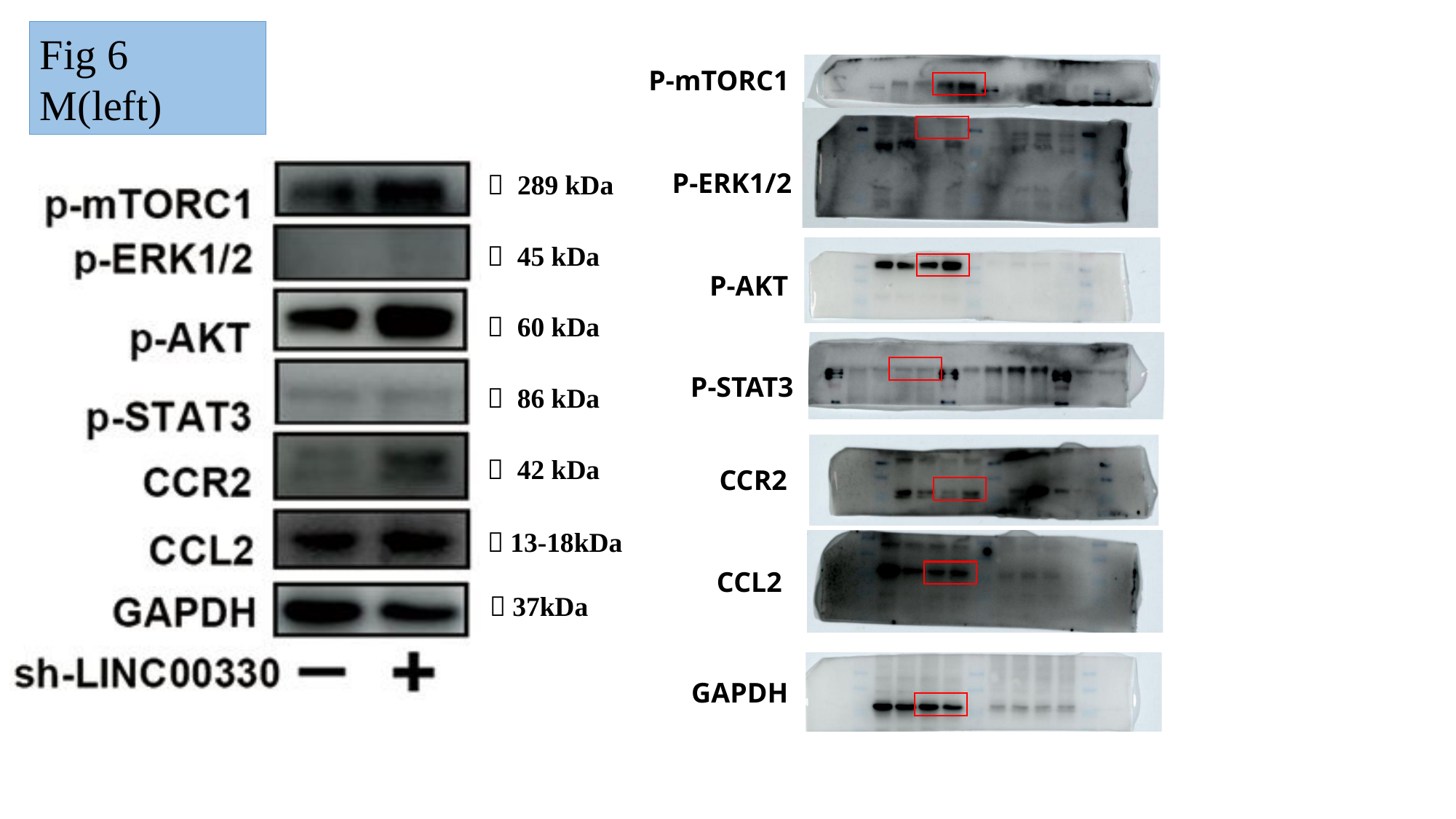

Fig 6 M(left)
P-mTORC1
P-ERK1/2
〜 289 kDa
〜 45 kDa
P-AKT
〜 60 kDa
P-STAT3
〜 86 kDa
〜 42 kDa
CCR2
〜13-18kDa
CCL2
〜37kDa
GAPDH

## Slide 29
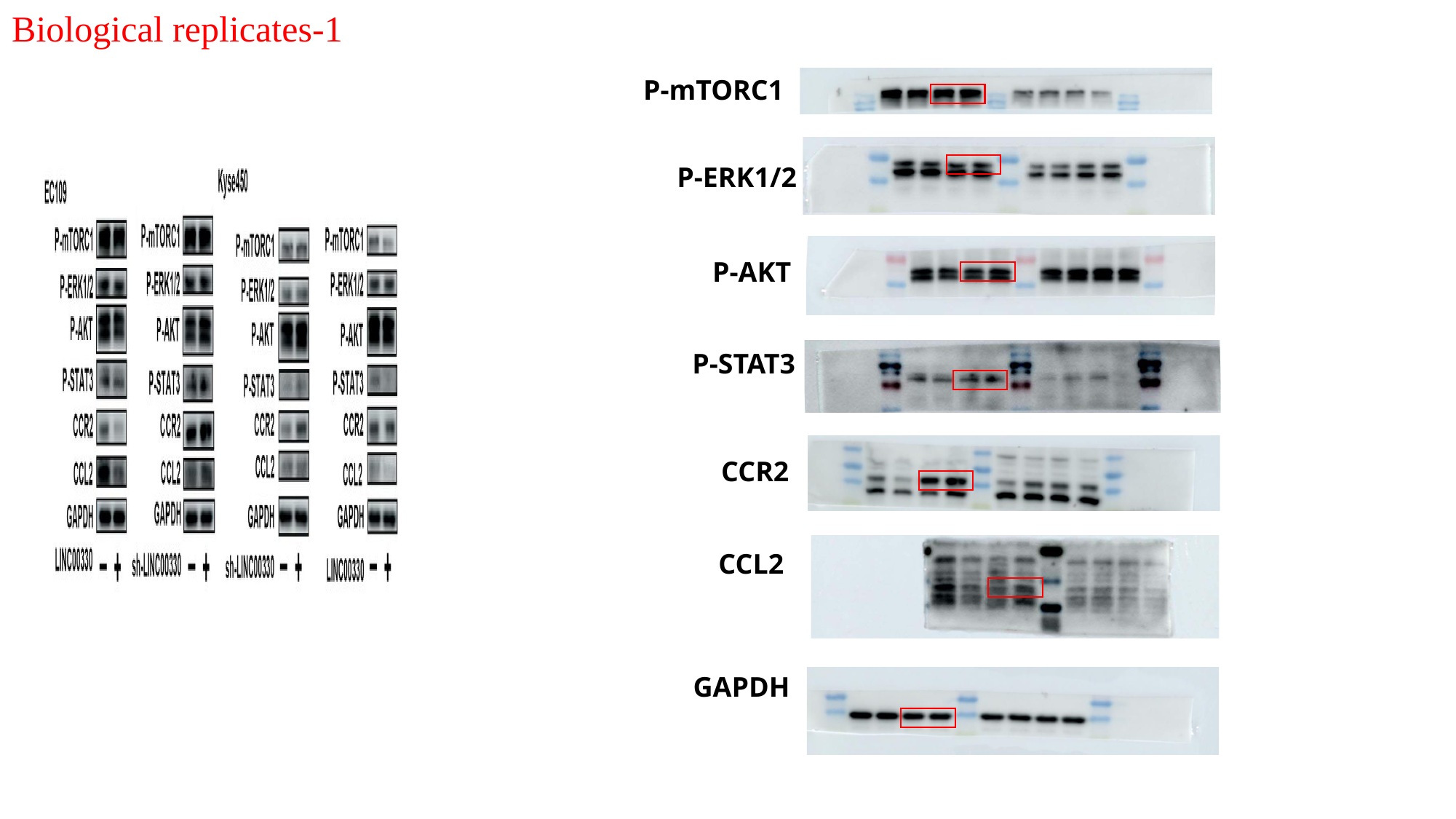

Biological replicates-1
P-mTORC1
P-ERK1/2
P-AKT
P-STAT3
CCR2
CCL2
GAPDH

## Slide 30
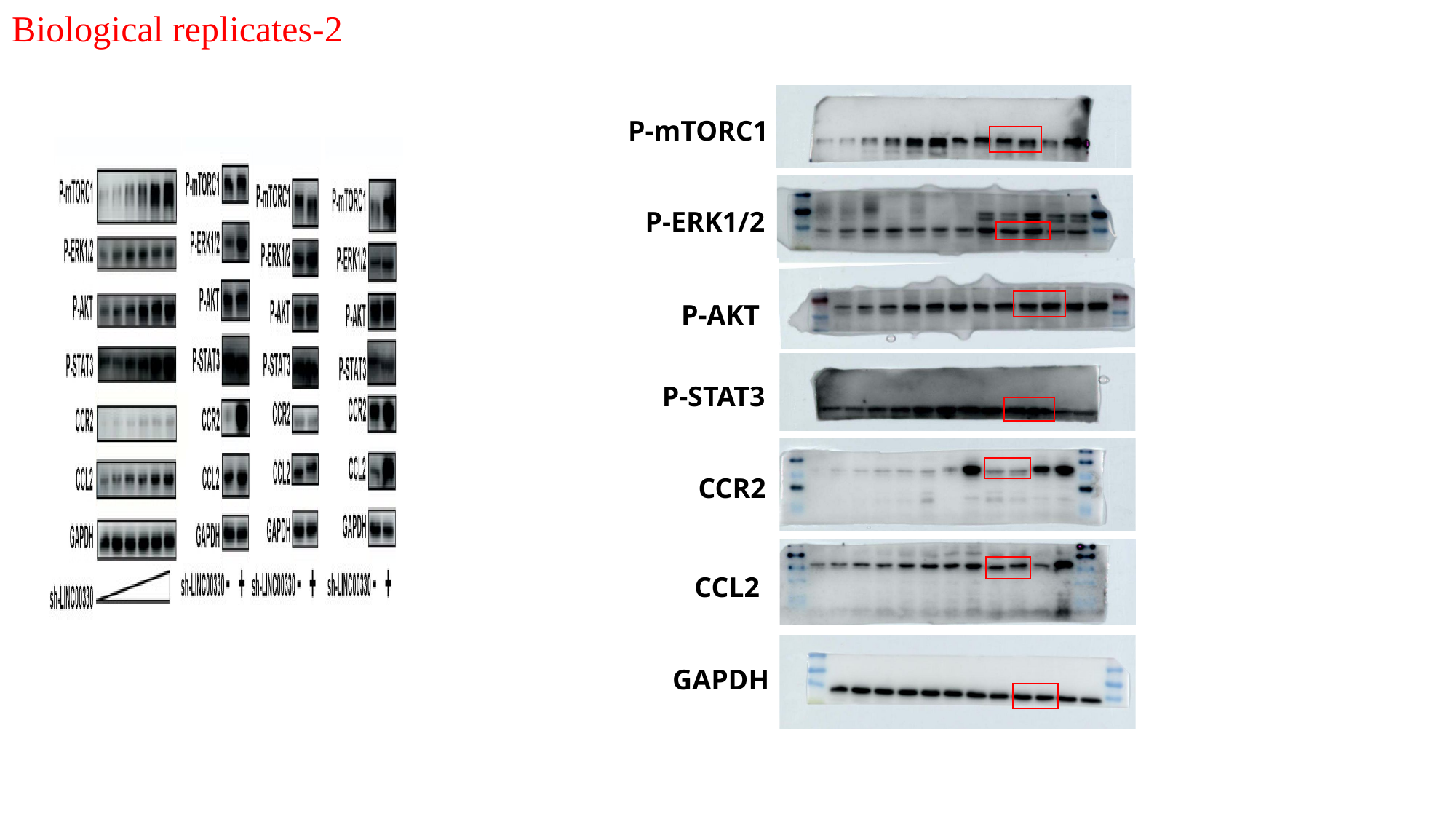

Biological replicates-2
P-mTORC1
P-ERK1/2
P-AKT
P-STAT3
CCR2
CCL2
GAPDH

## Slide 31
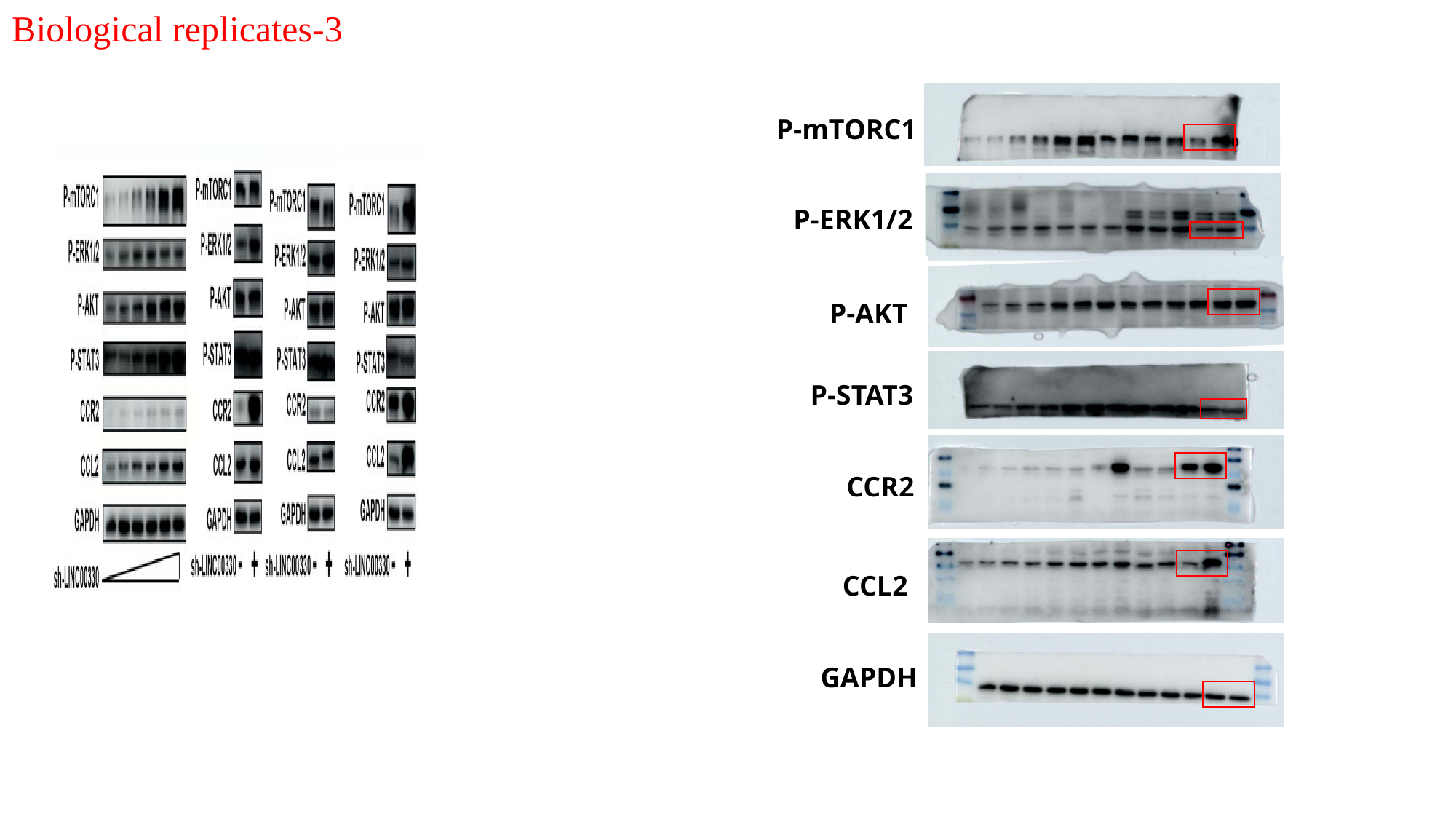

Biological replicates-3
P-mTORC1
P-ERK1/2
P-AKT
P-STAT3
CCR2
CCL2
GAPDH

## Slide 32
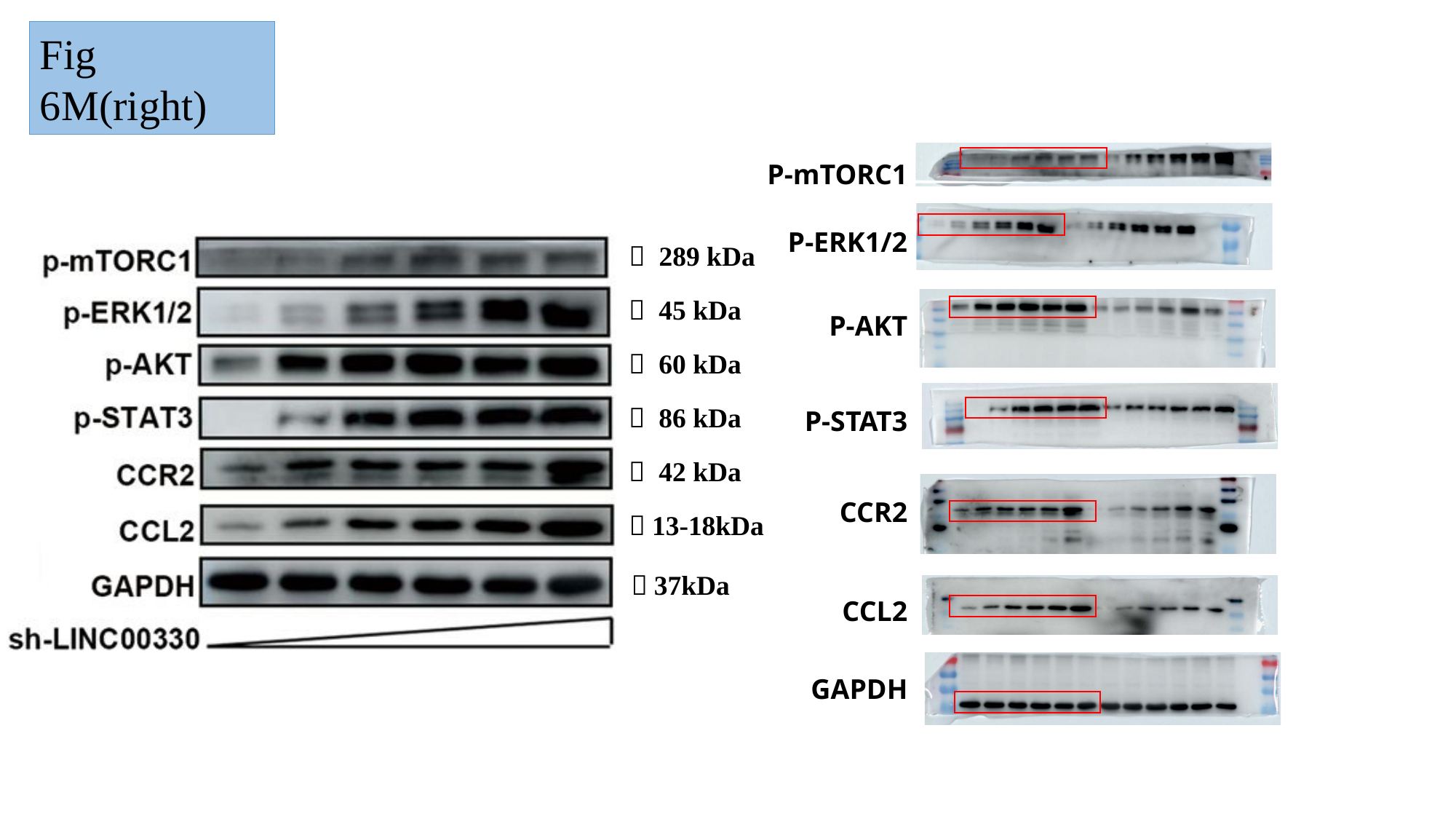

Fig 6M(right)
P-mTORC1
P-ERK1/2
〜 289 kDa
〜 45 kDa
P-AKT
〜 60 kDa
〜 86 kDa
P-STAT3
〜 42 kDa
CCR2
〜13-18kDa
〜37kDa
CCL2
GAPDH

## Slide 33
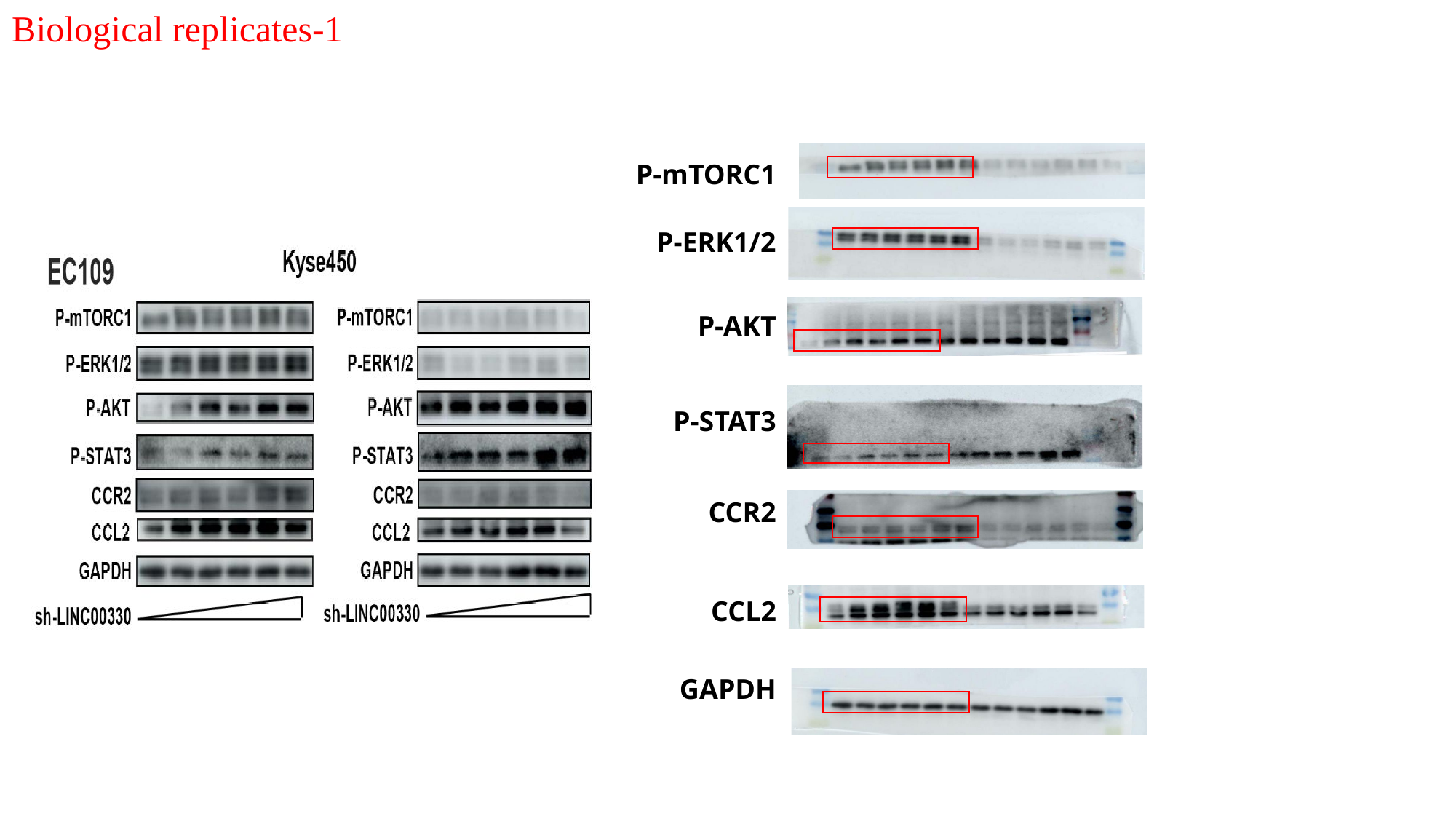

Biological replicates-1
P-mTORC1
P-ERK1/2
P-AKT
P-STAT3
CCR2
CCL2
GAPDH

## Slide 34
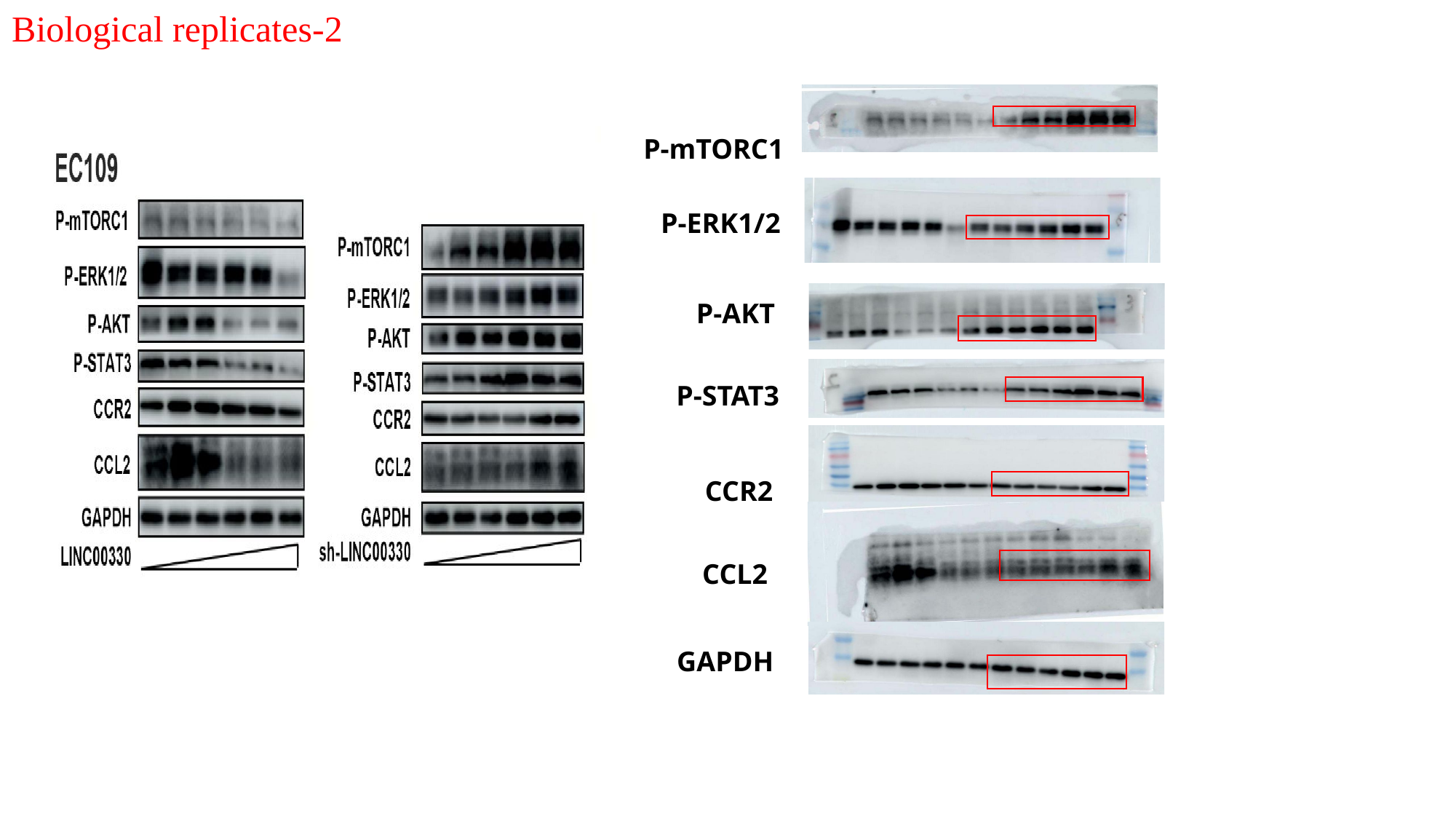

Biological replicates-2
P-mTORC1
P-ERK1/2
P-AKT
P-STAT3
CCR2
CCL2
GAPDH

## Slide 35
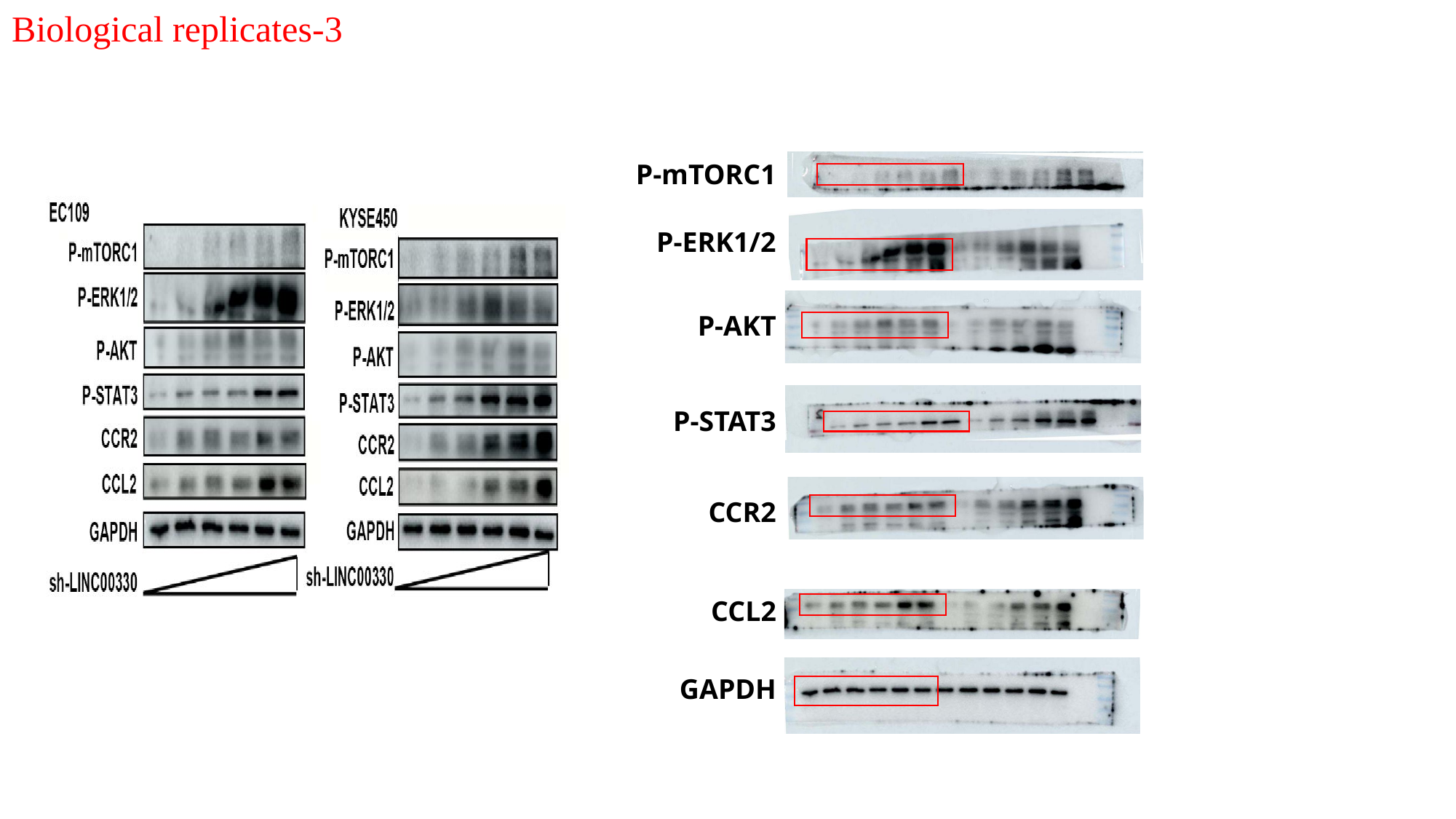

Biological replicates-3
P-mTORC1
P-ERK1/2
P-AKT
P-STAT3
CCR2
CCL2
GAPDH

## Slide 36
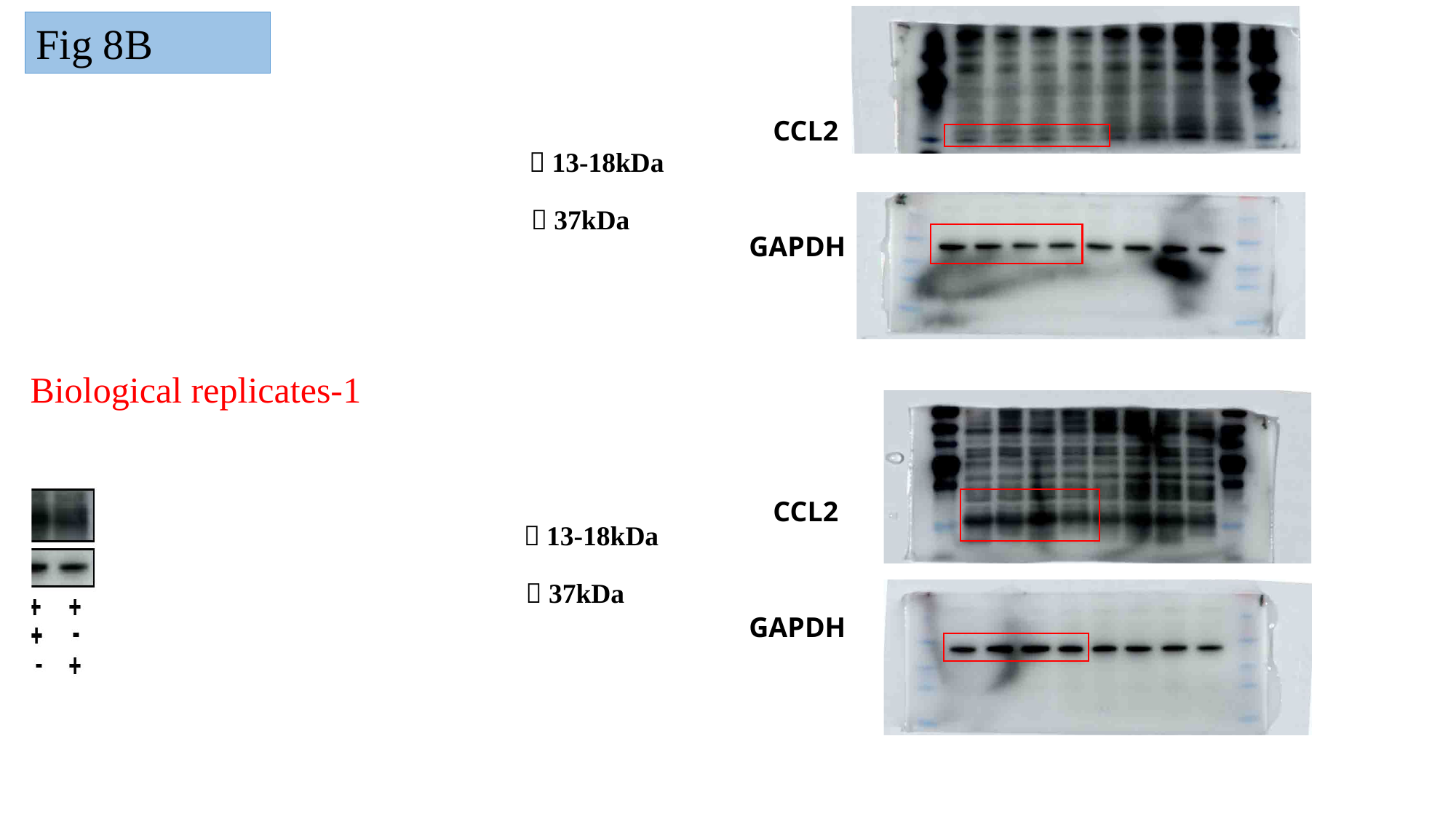

Fig 8B
CCL2
〜13-18kDa
〜37kDa
GAPDH
Biological replicates-1
CCL2
〜13-18kDa
〜37kDa
GAPDH

## Slide 37
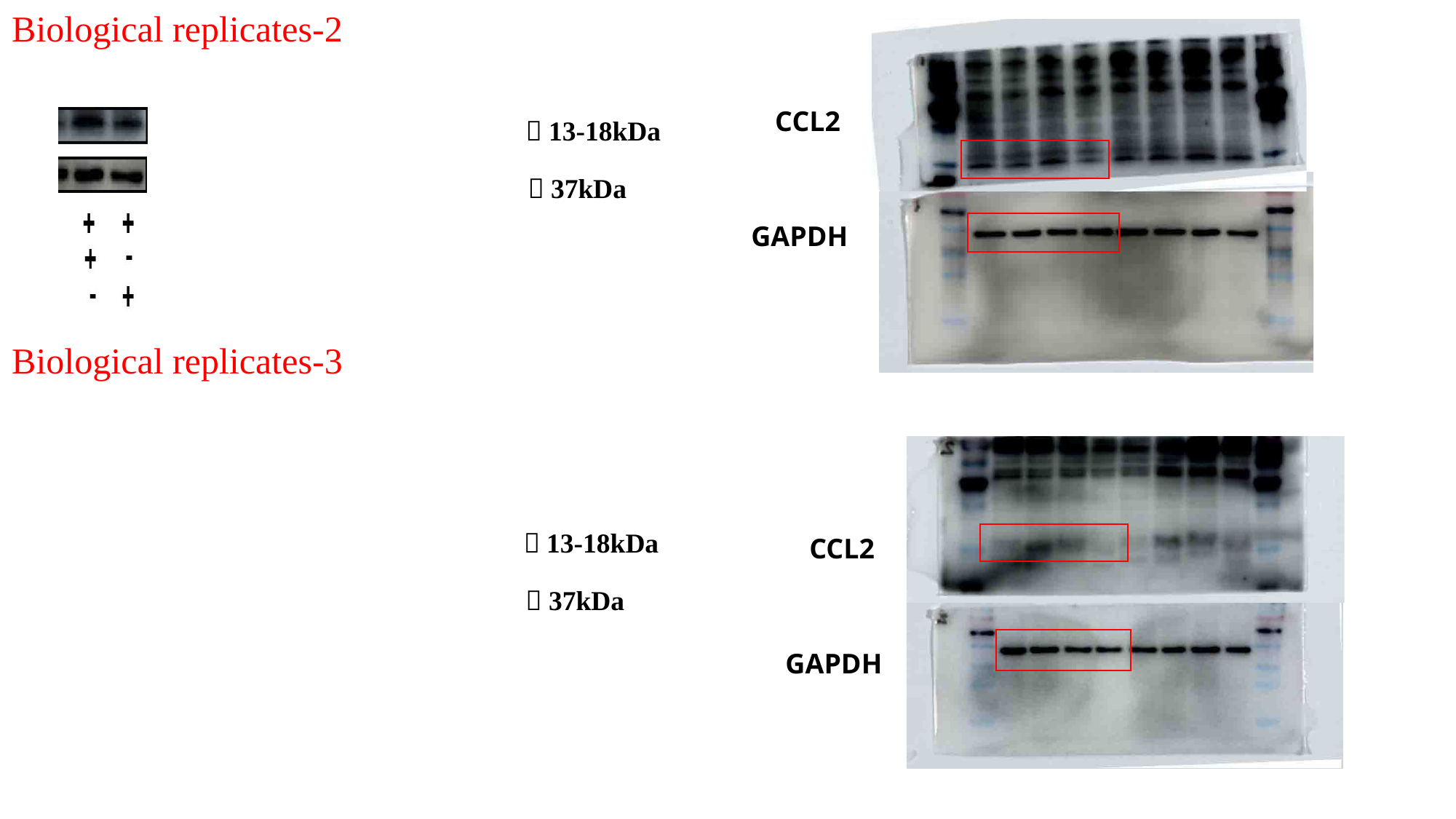

Biological replicates-2
CCL2
〜13-18kDa
〜37kDa
GAPDH
Biological replicates-3
〜13-18kDa
CCL2
〜37kDa
GAPDH

## Slide 38
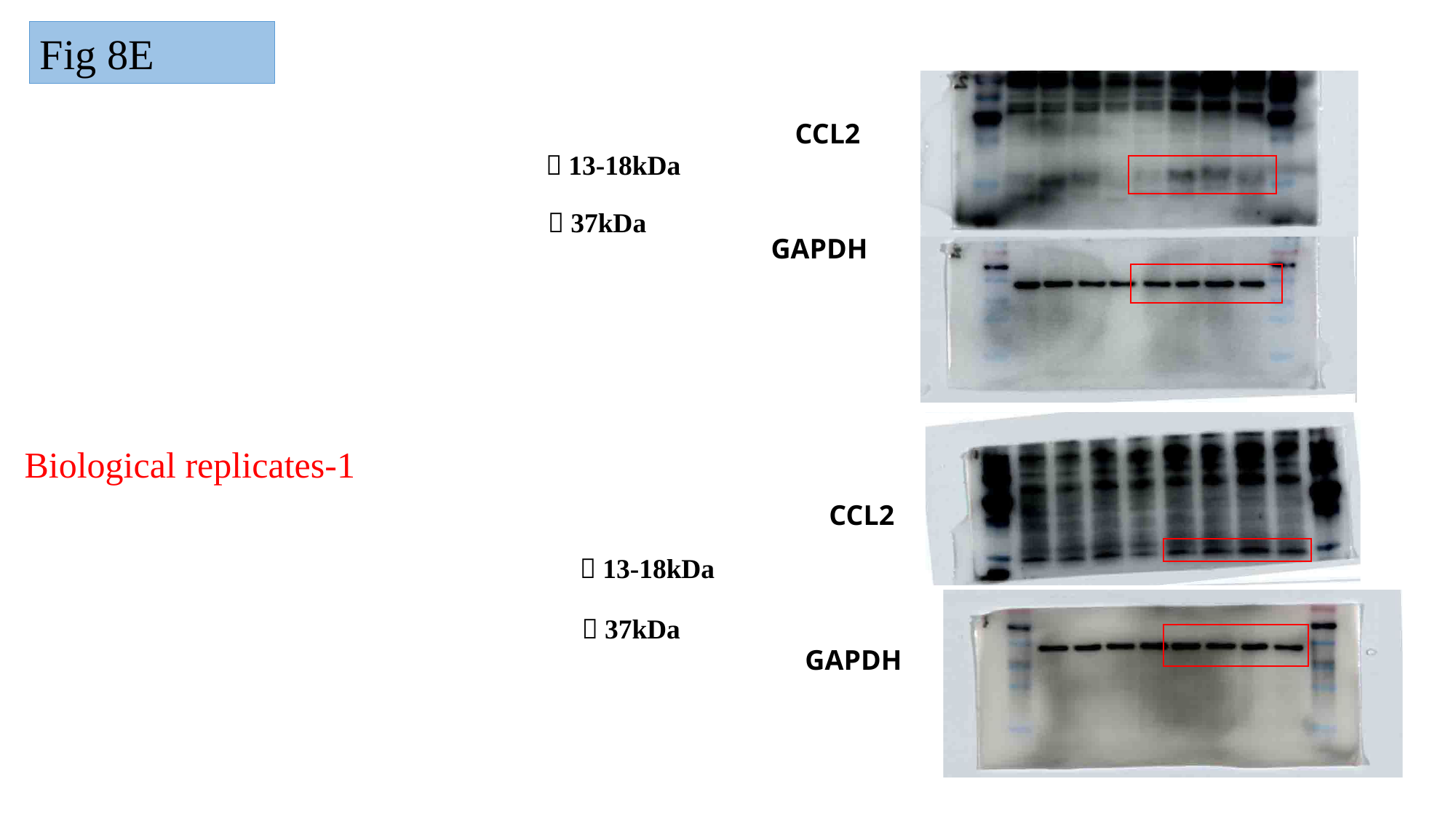

Fig 8E
CCL2
〜13-18kDa
〜37kDa
GAPDH
Biological replicates-1
CCL2
〜13-18kDa
〜37kDa
GAPDH

## Slide 39
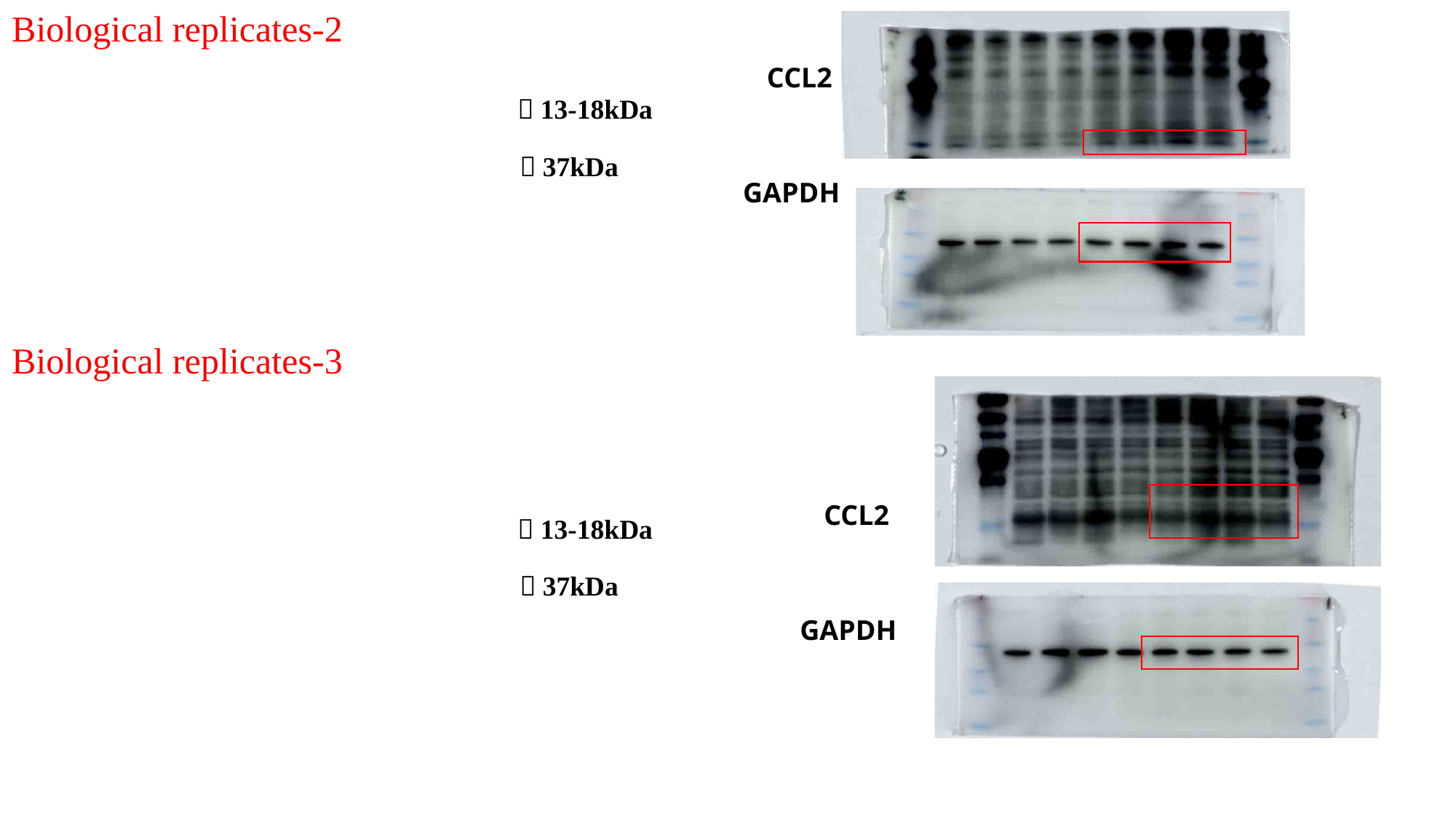

Biological replicates-2
CCL2
〜13-18kDa
〜37kDa
GAPDH
Biological replicates-3
CCL2
〜13-18kDa
〜37kDa
GAPDH

## Slide 40
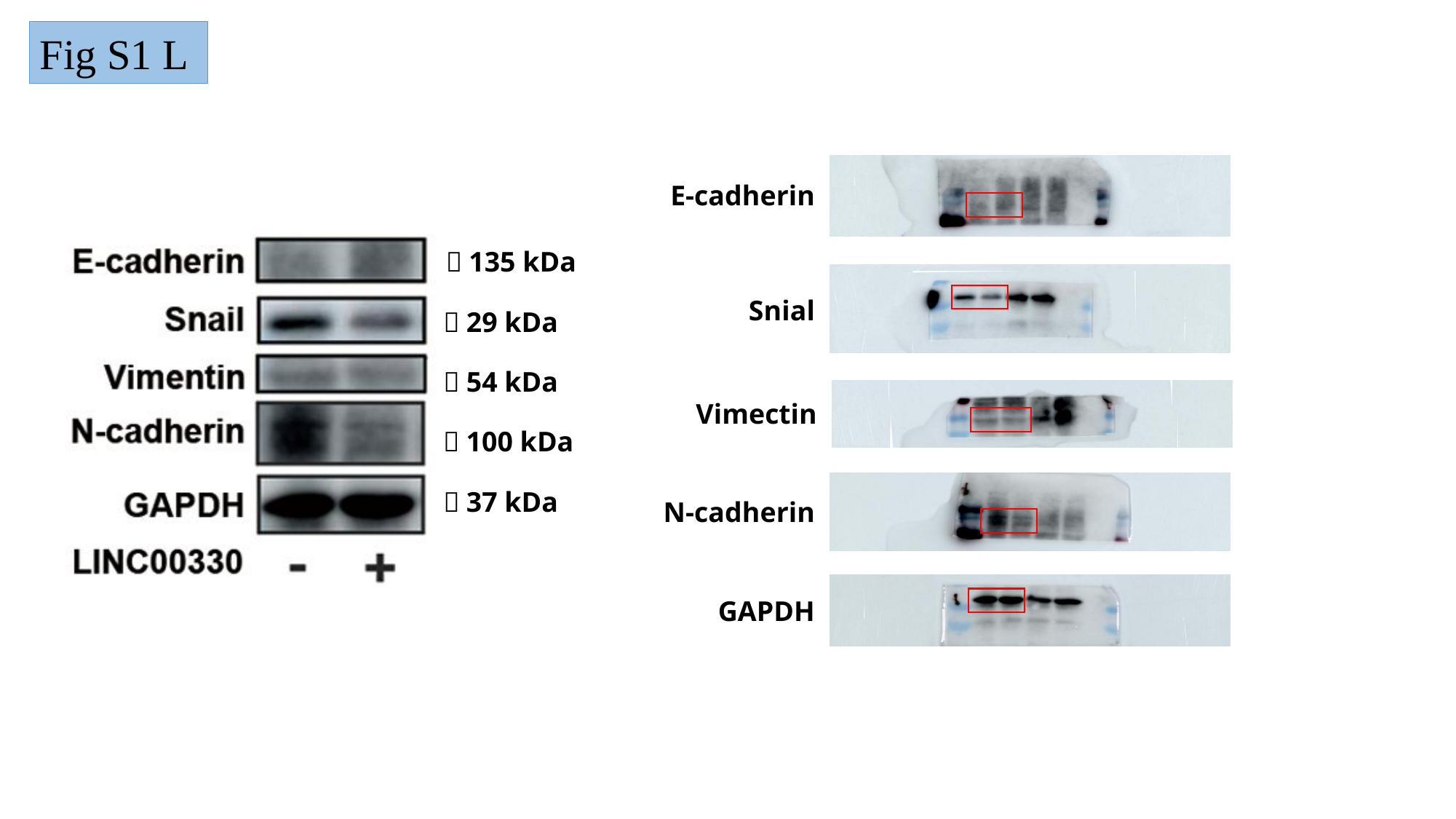

Fig S1 L
E-cadherin
〜135 kDa
Snial
〜29 kDa
〜54 kDa
Vimectin
〜100 kDa
〜37 kDa
N-cadherin
GAPDH

## Slide 41
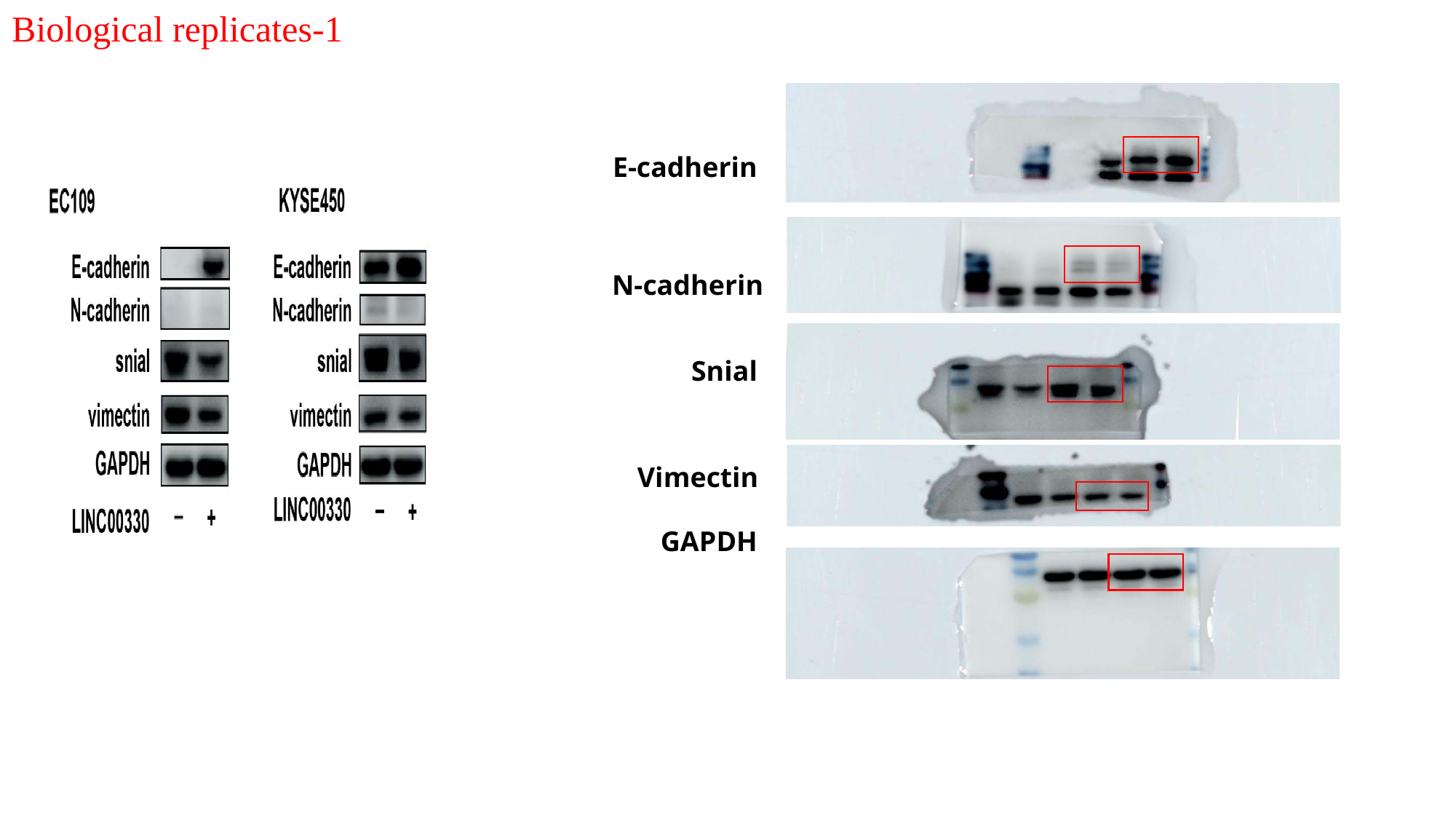

Biological replicates-1
E-cadherin
N-cadherin
Snial
Vimectin
GAPDH

## Slide 42
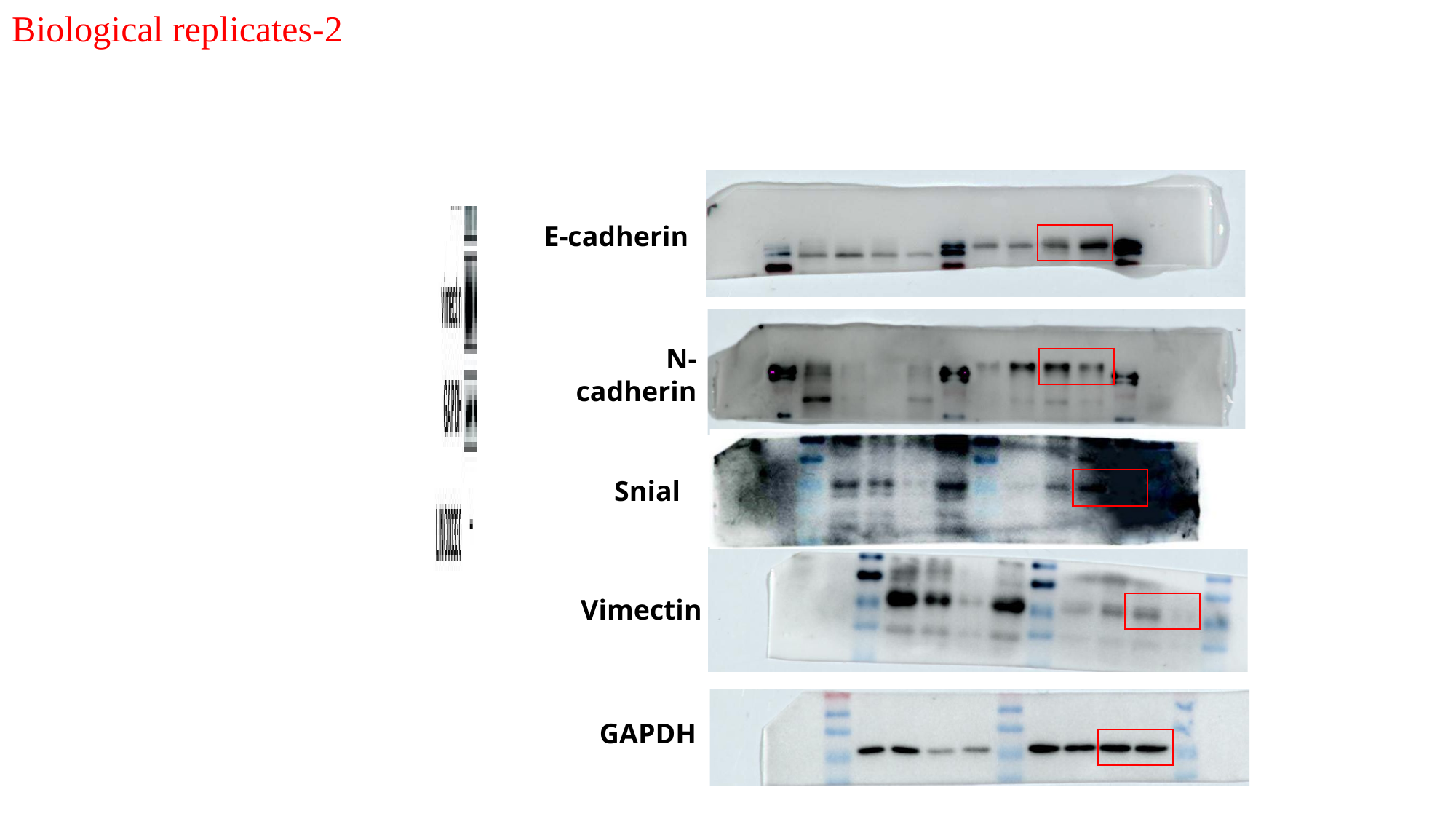

Biological replicates-2
E-cadherin
N-cadherin
Snial
Vimectin
GAPDH

## Slide 43
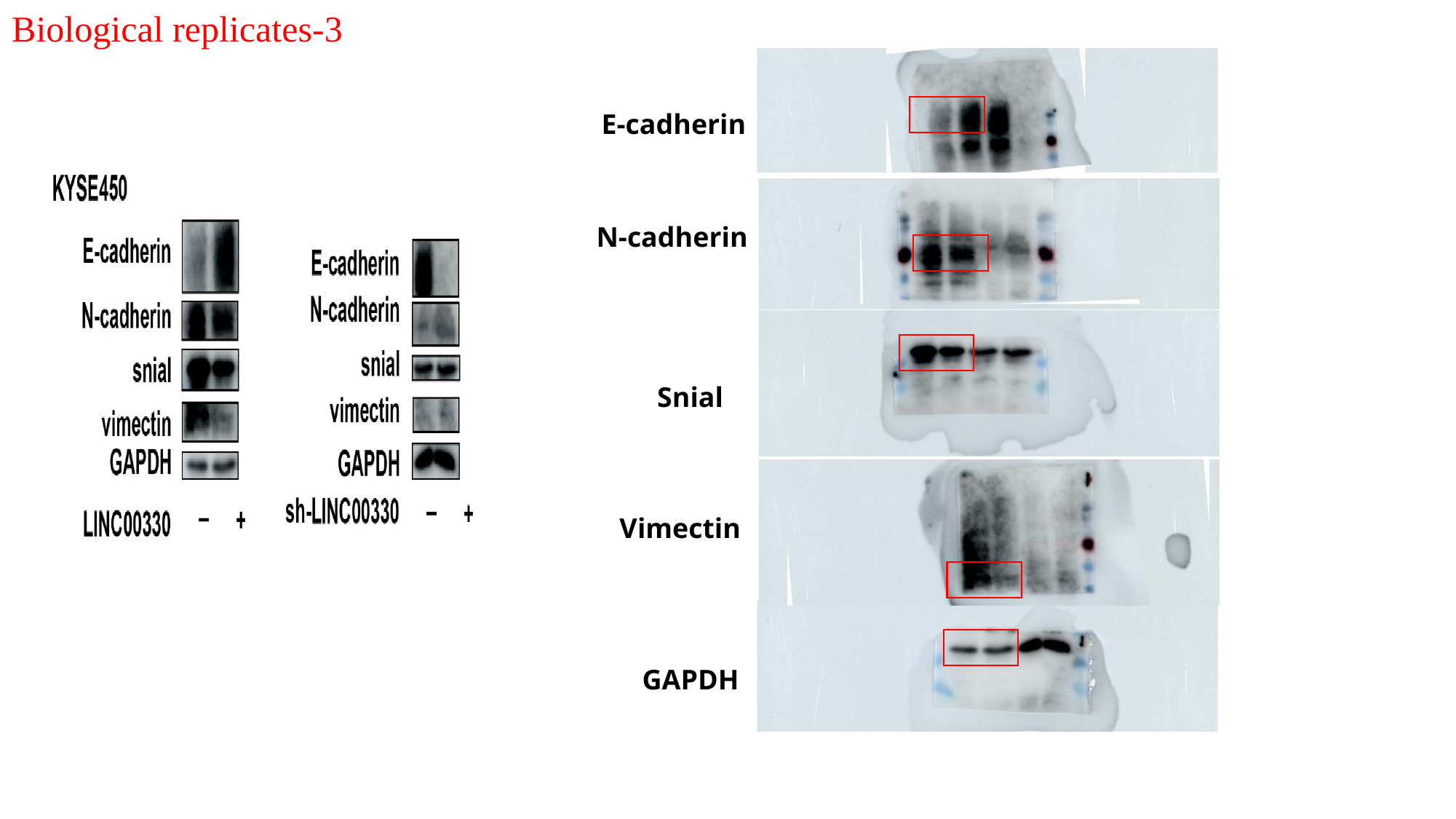

Biological replicates-3
E-cadherin
N-cadherin
Snial
Vimectin
GAPDH

## Slide 44
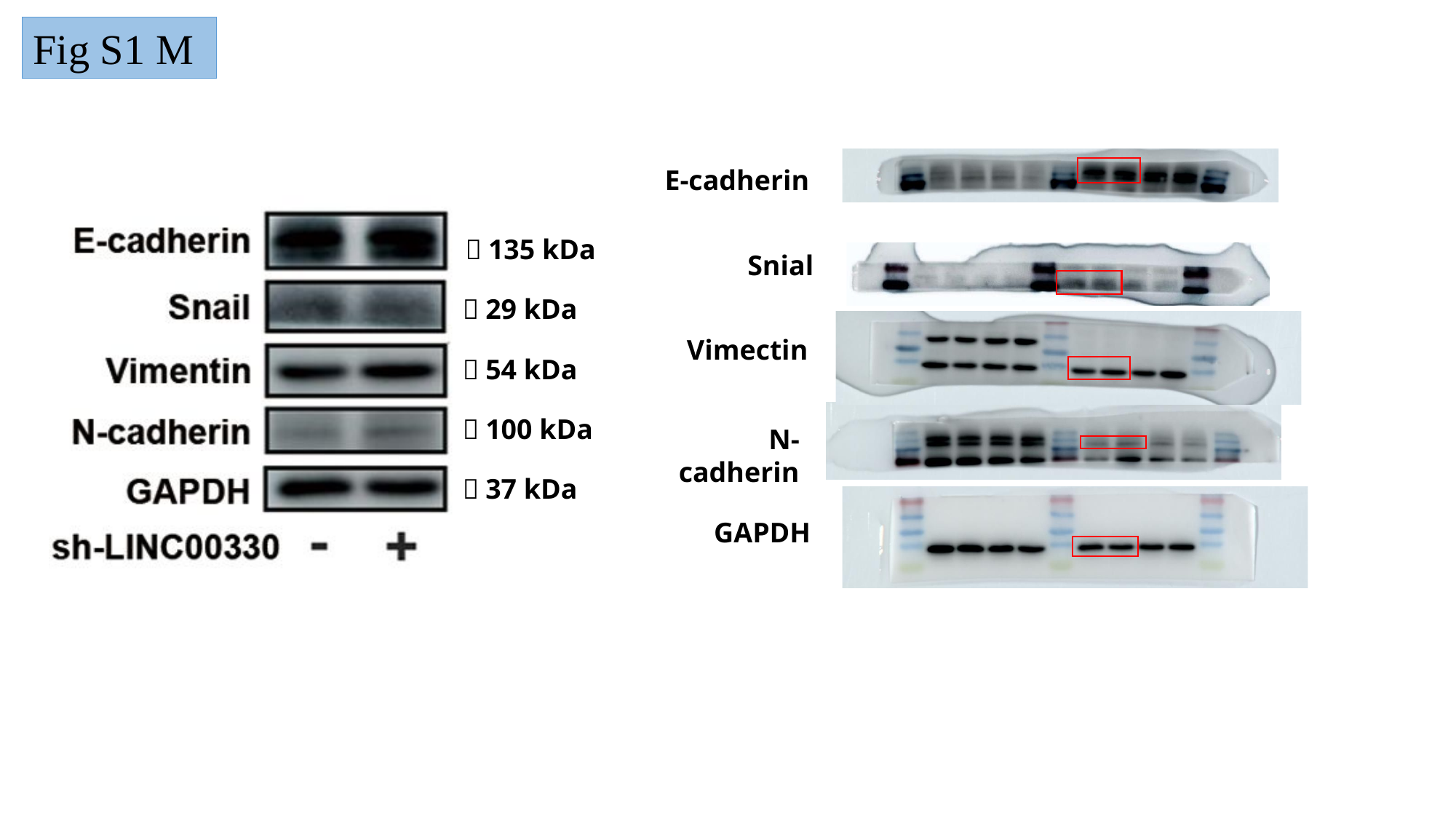

Fig S1 M
E-cadherin
〜135 kDa
Snial
〜29 kDa
Vimectin
〜54 kDa
〜100 kDa
N-cadherin
〜37 kDa
GAPDH

## Slide 45
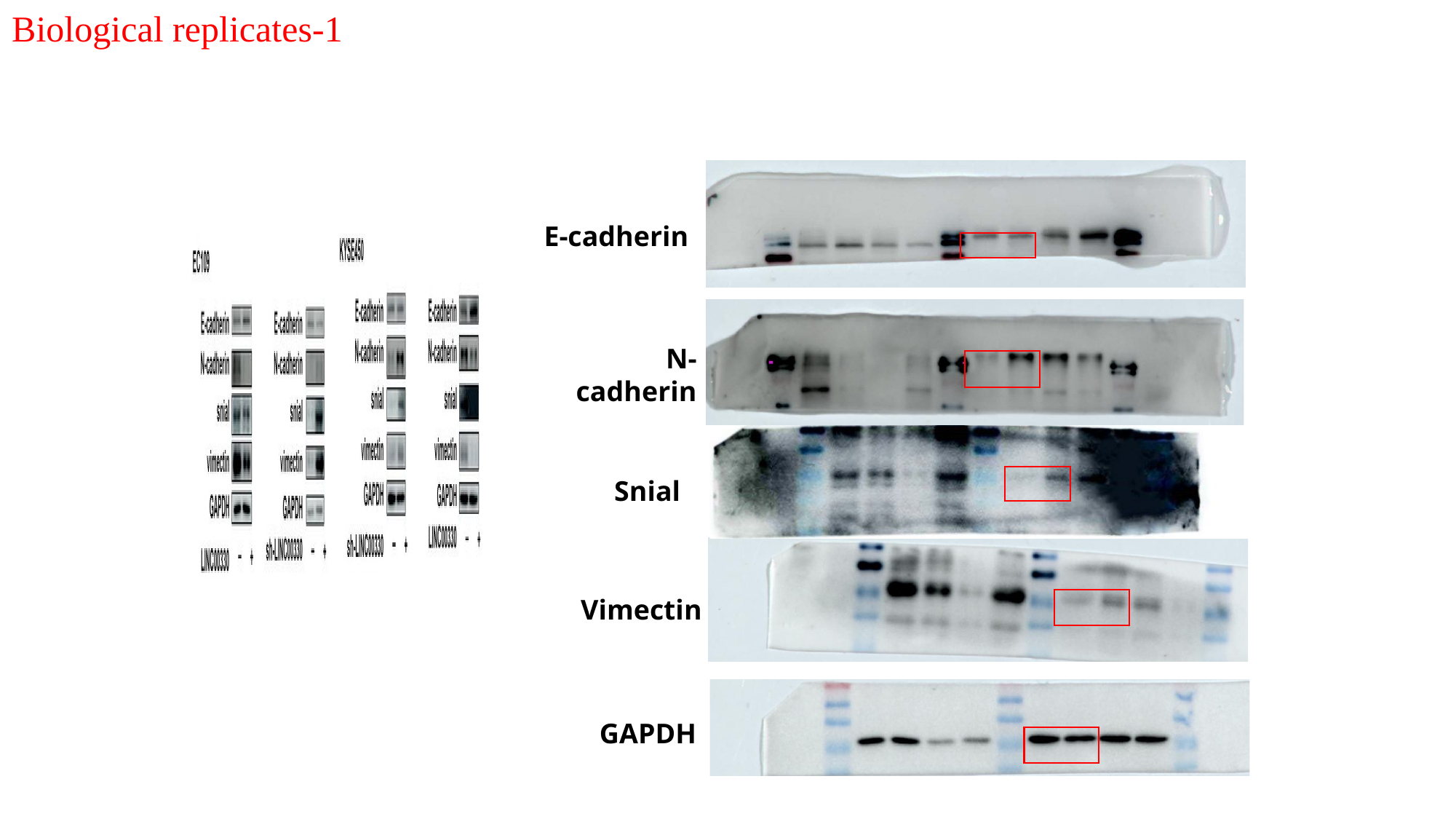

Biological replicates-1
E-cadherin
N-cadherin
Snial
Vimectin
GAPDH

## Slide 46
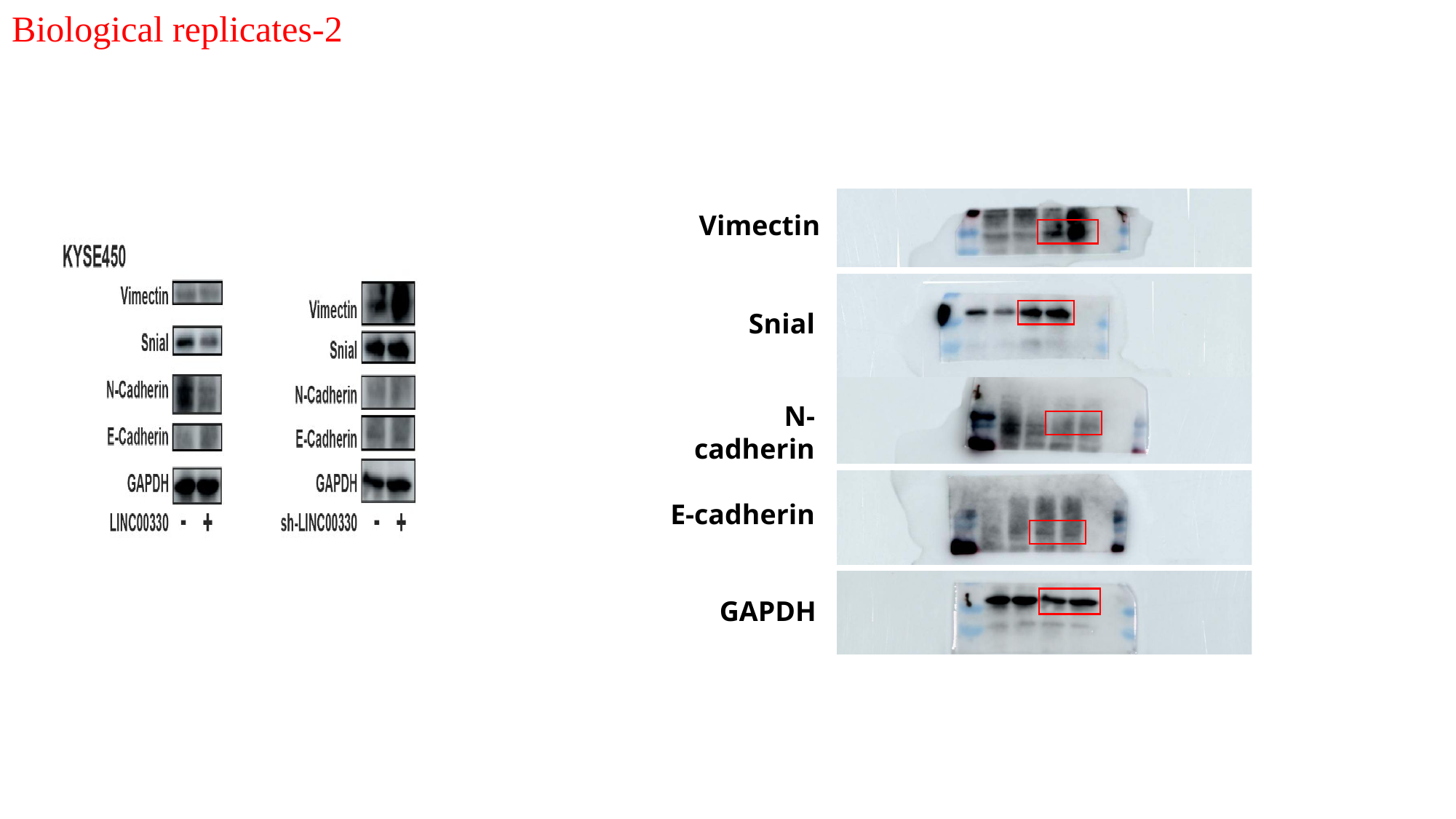

Biological replicates-2
Vimectin
Snial
N-cadherin
E-cadherin
GAPDH

## Slide 47
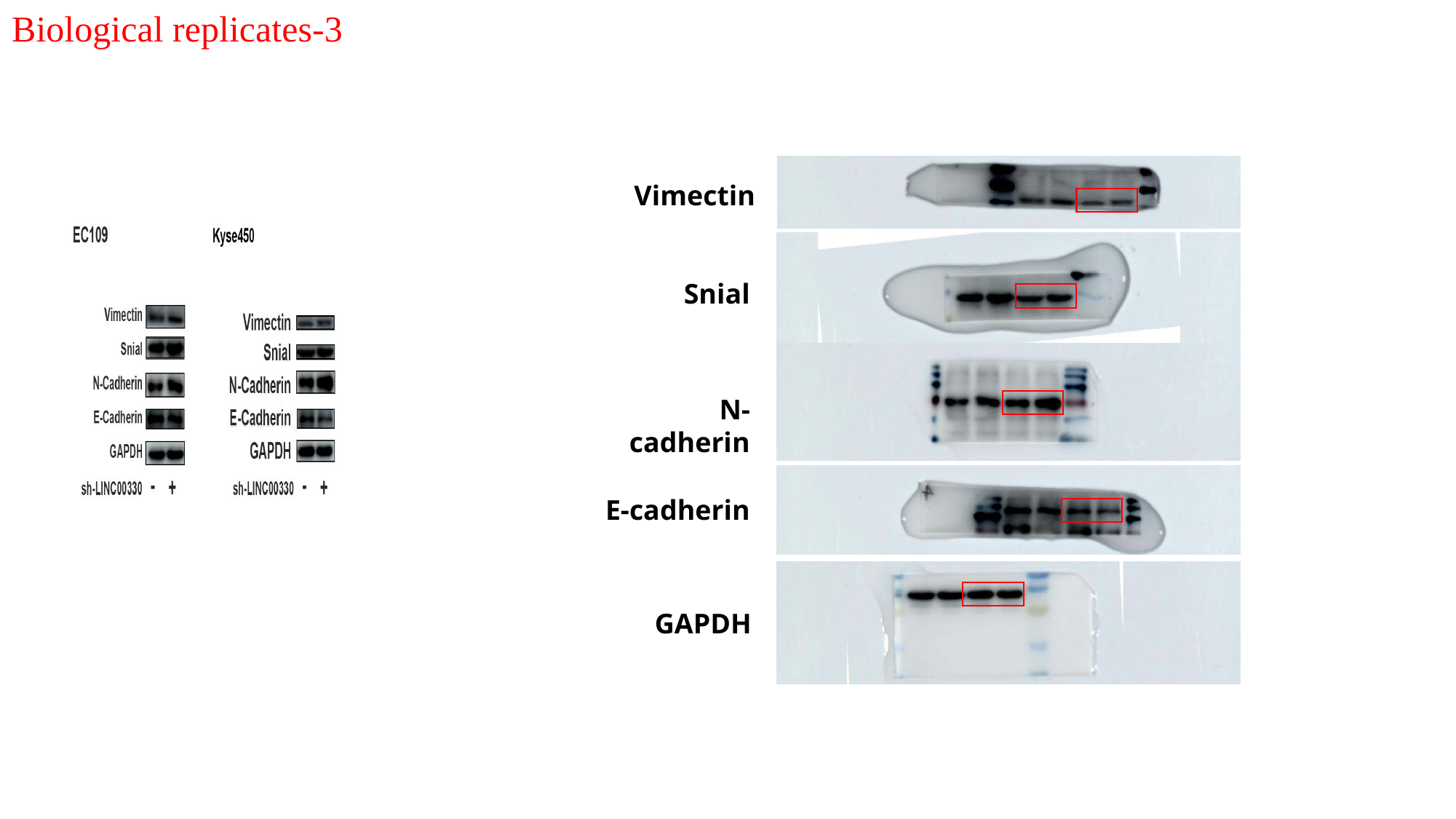

Biological replicates-3
Vimectin
Snial
N-cadherin
E-cadherin
GAPDH

## Slide 48
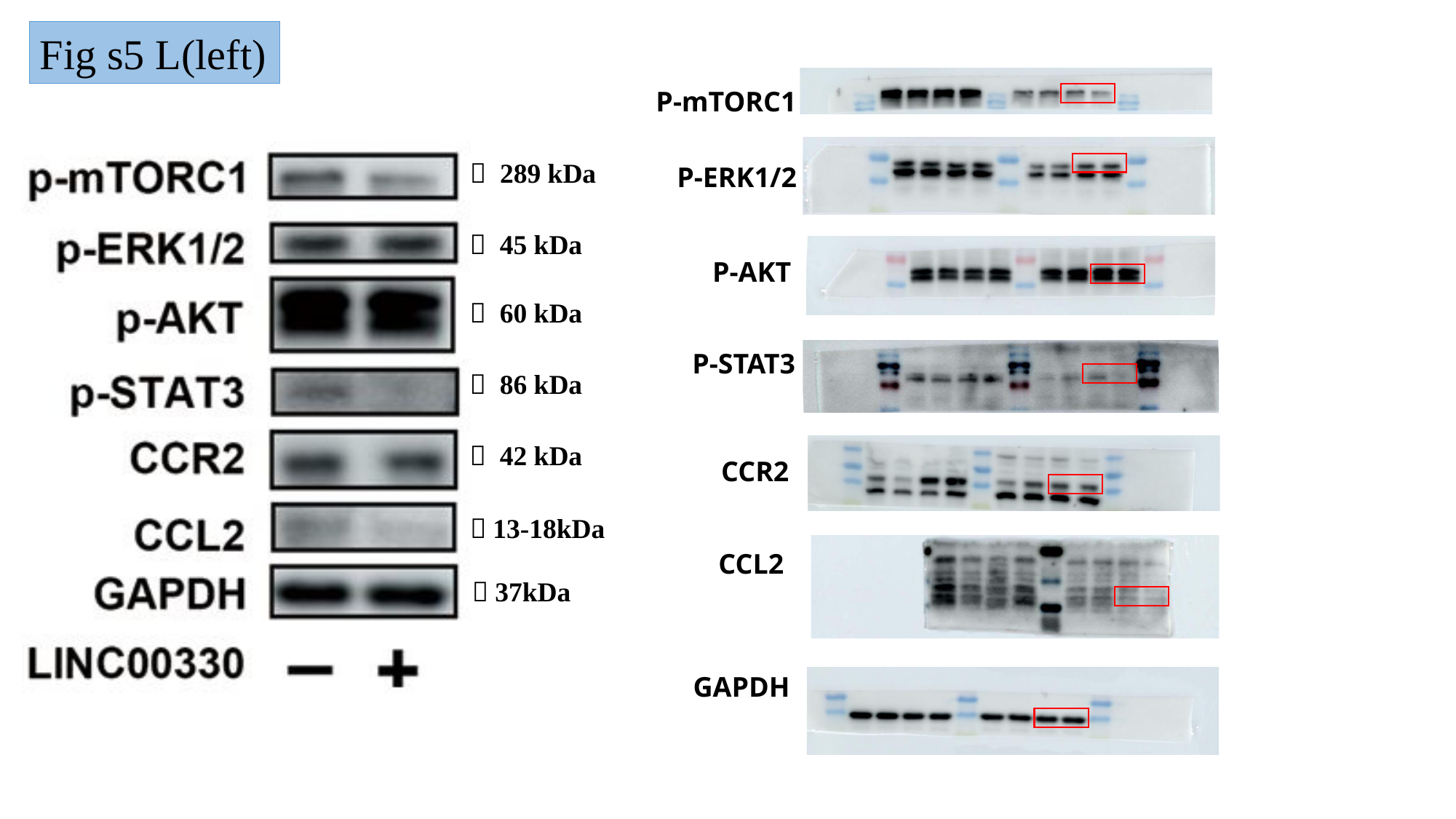

Fig s5 L(left)
P-mTORC1
〜 289 kDa
P-ERK1/2
〜 45 kDa
P-AKT
〜 60 kDa
P-STAT3
〜 86 kDa
〜 42 kDa
CCR2
〜13-18kDa
CCL2
〜37kDa
GAPDH

## Slide 49
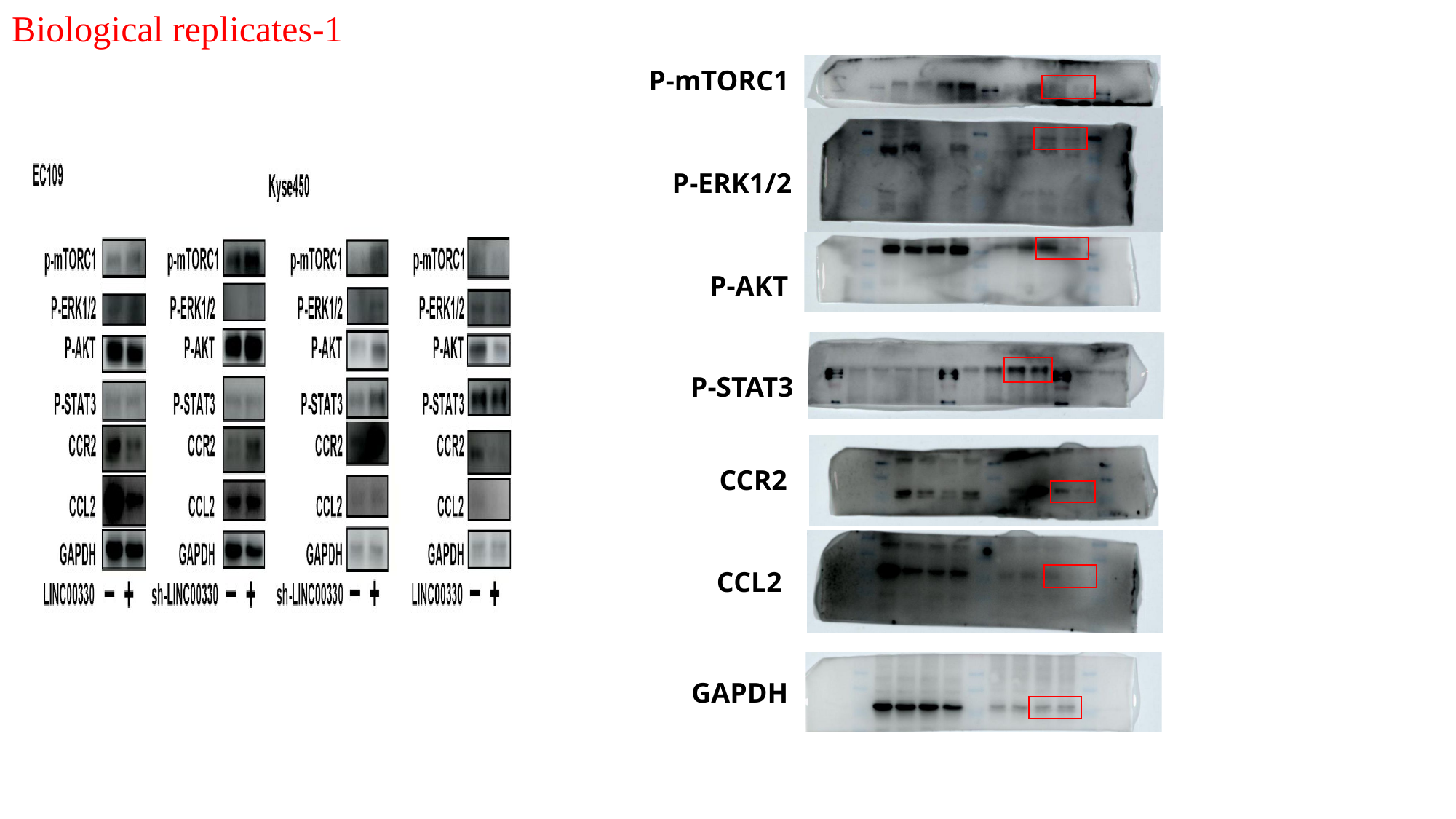

Biological replicates-1
P-mTORC1
P-ERK1/2
P-AKT
P-STAT3
CCR2
CCL2
GAPDH

## Slide 50
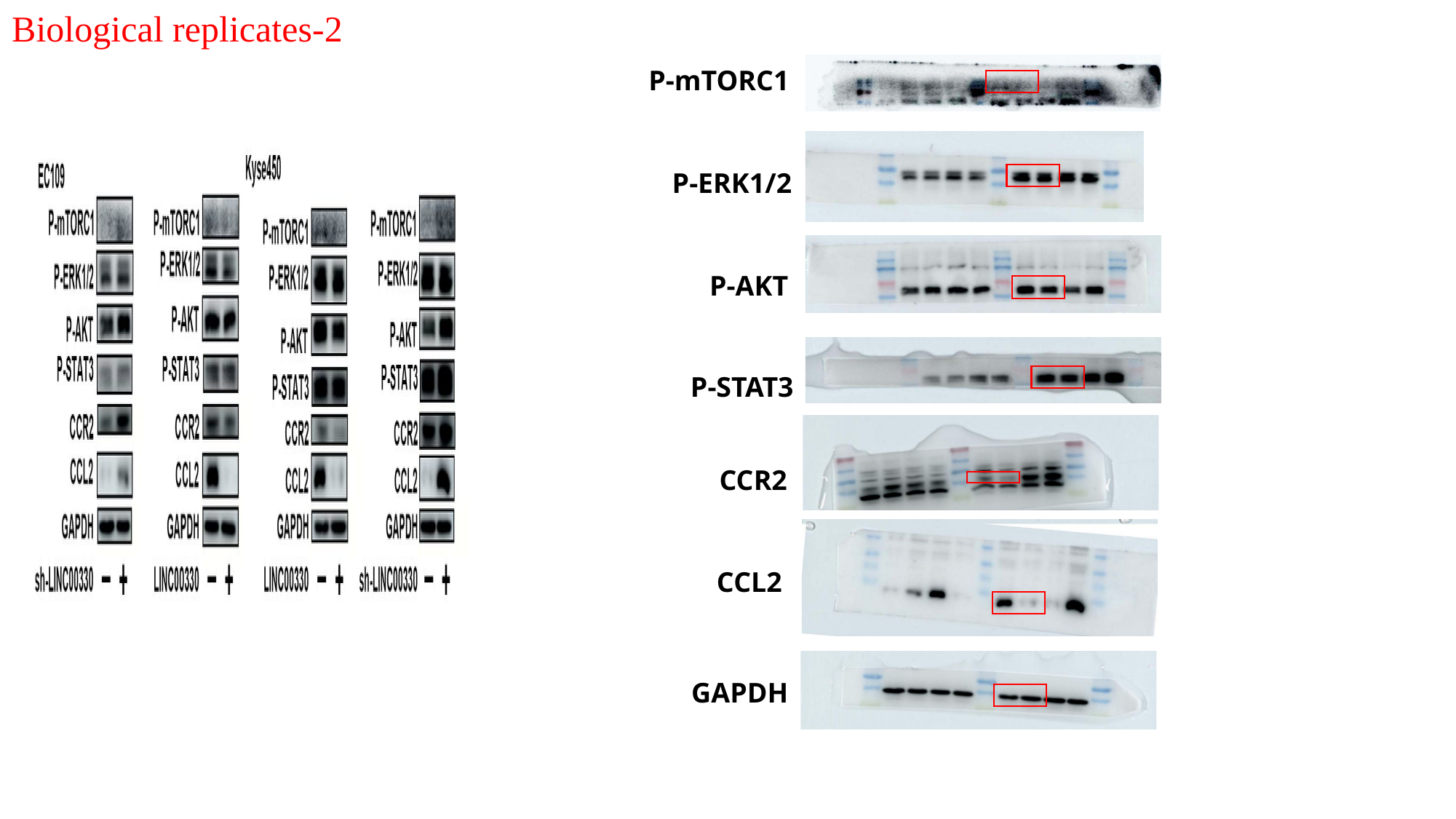

Biological replicates-2
P-mTORC1
P-ERK1/2
P-AKT
P-STAT3
CCR2
CCL2
GAPDH

## Slide 51
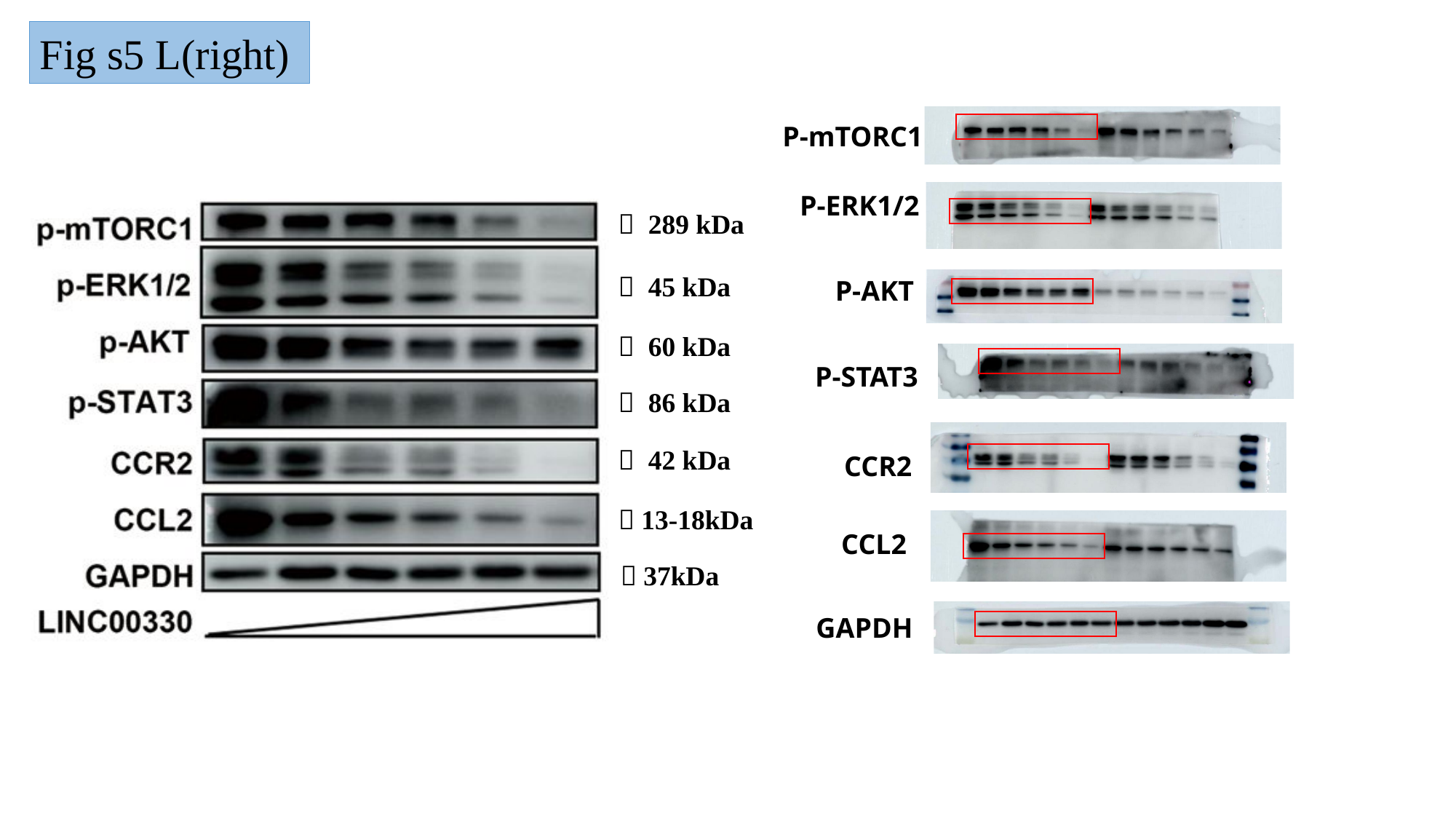

Fig s5 L(right)
P-mTORC1
P-ERK1/2
〜 289 kDa
〜 45 kDa
P-AKT
〜 60 kDa
P-STAT3
〜 86 kDa
〜 42 kDa
CCR2
〜13-18kDa
CCL2
〜37kDa
GAPDH

## Slide 52
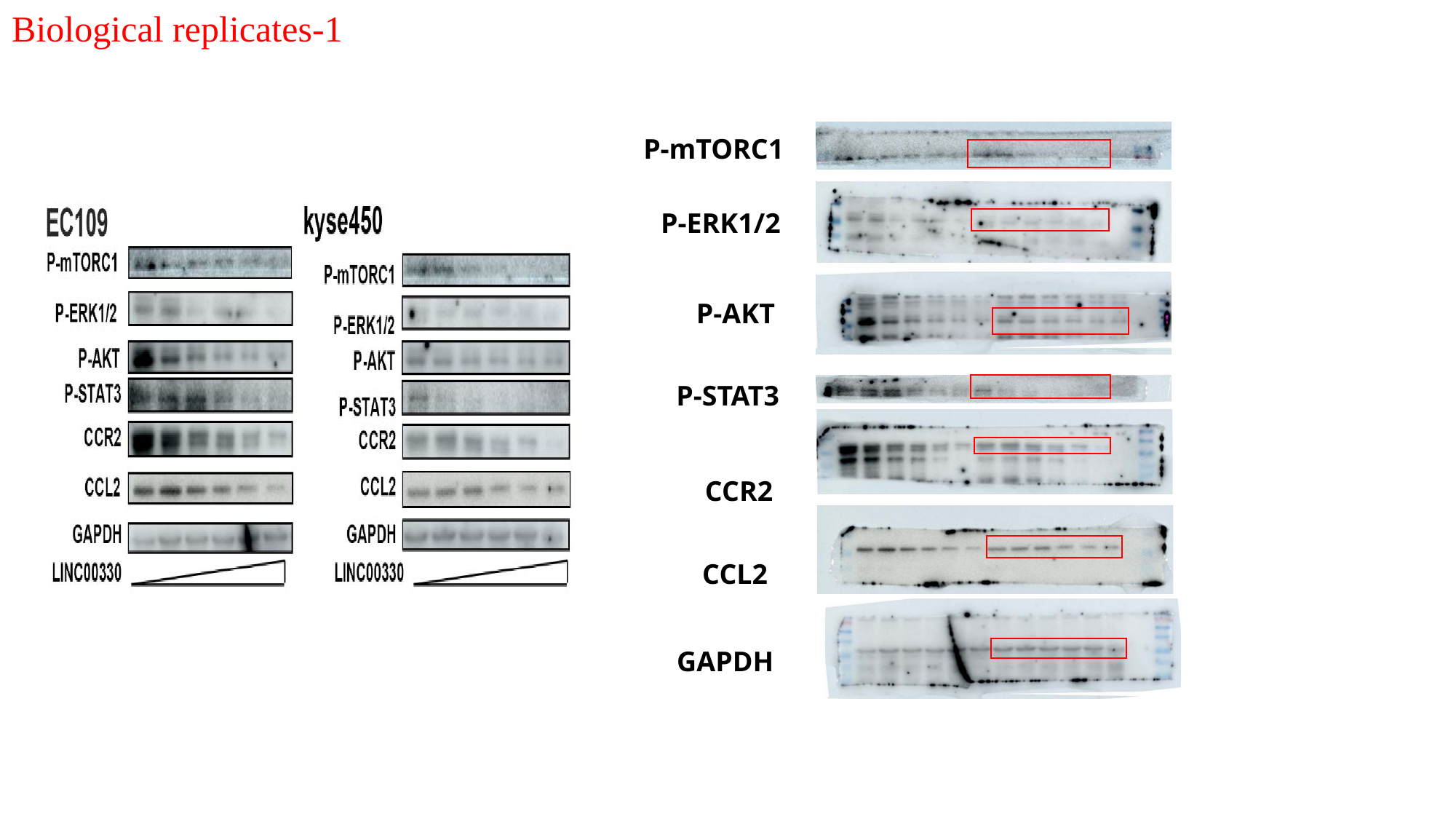

Biological replicates-1
P-mTORC1
P-ERK1/2
P-AKT
P-STAT3
CCR2
CCL2
GAPDH

## Slide 53
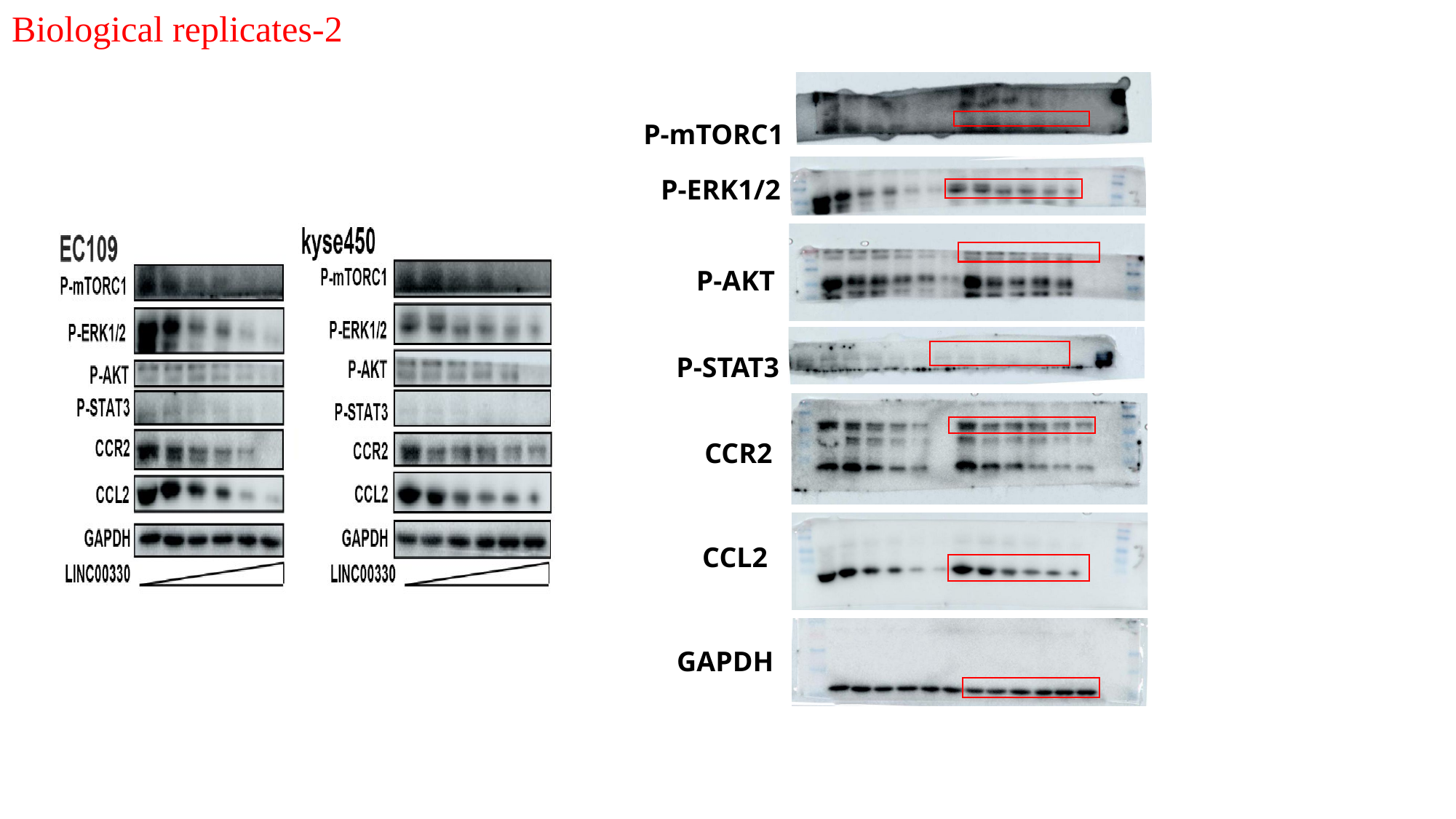

Biological replicates-2
P-mTORC1
P-ERK1/2
P-AKT
P-STAT3
CCR2
CCL2
GAPDH

## Slide 54
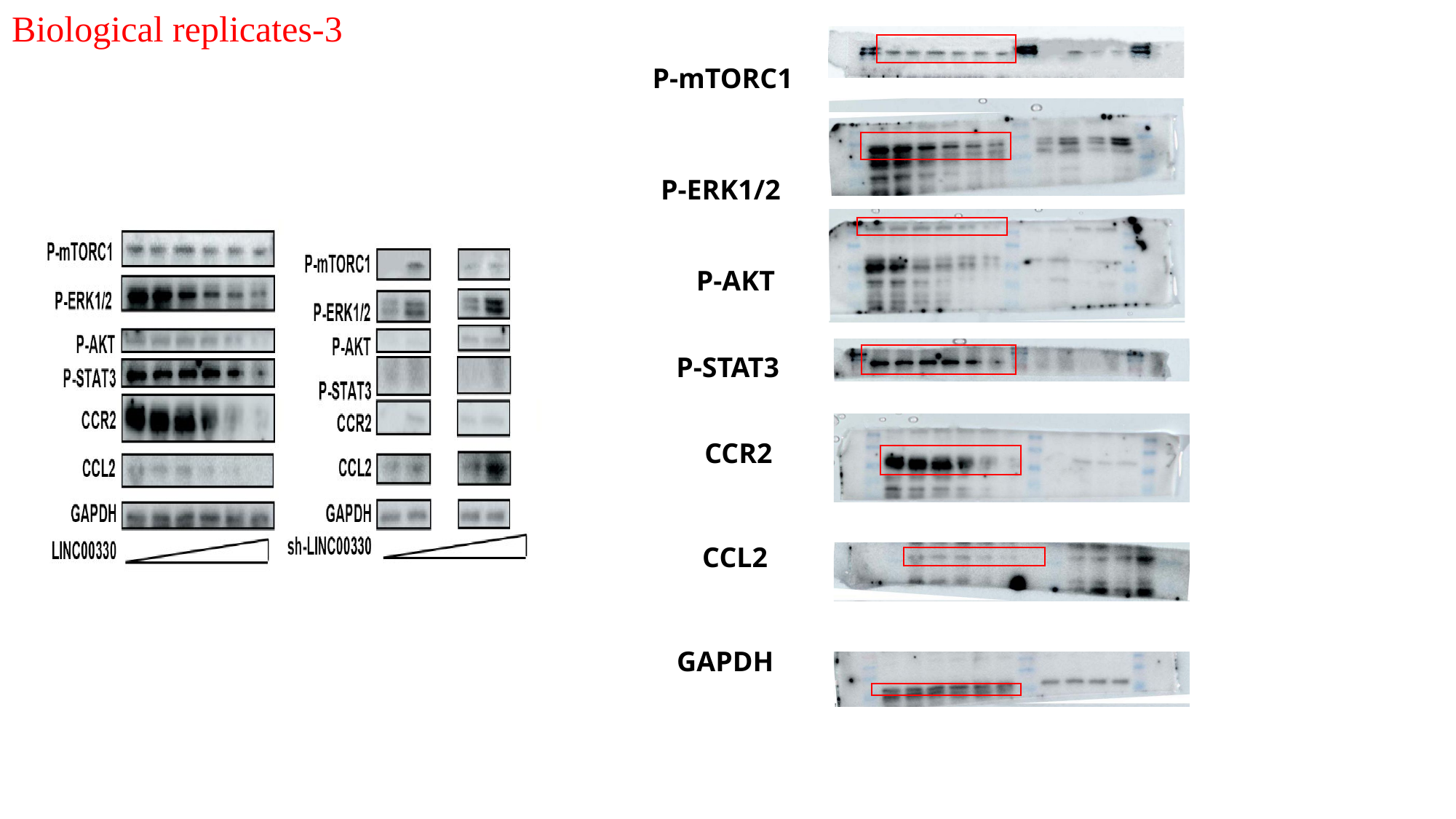

Biological replicates-3
P-mTORC1
P-ERK1/2
P-AKT
P-STAT3
CCR2
CCL2
GAPDH

## Slide 55
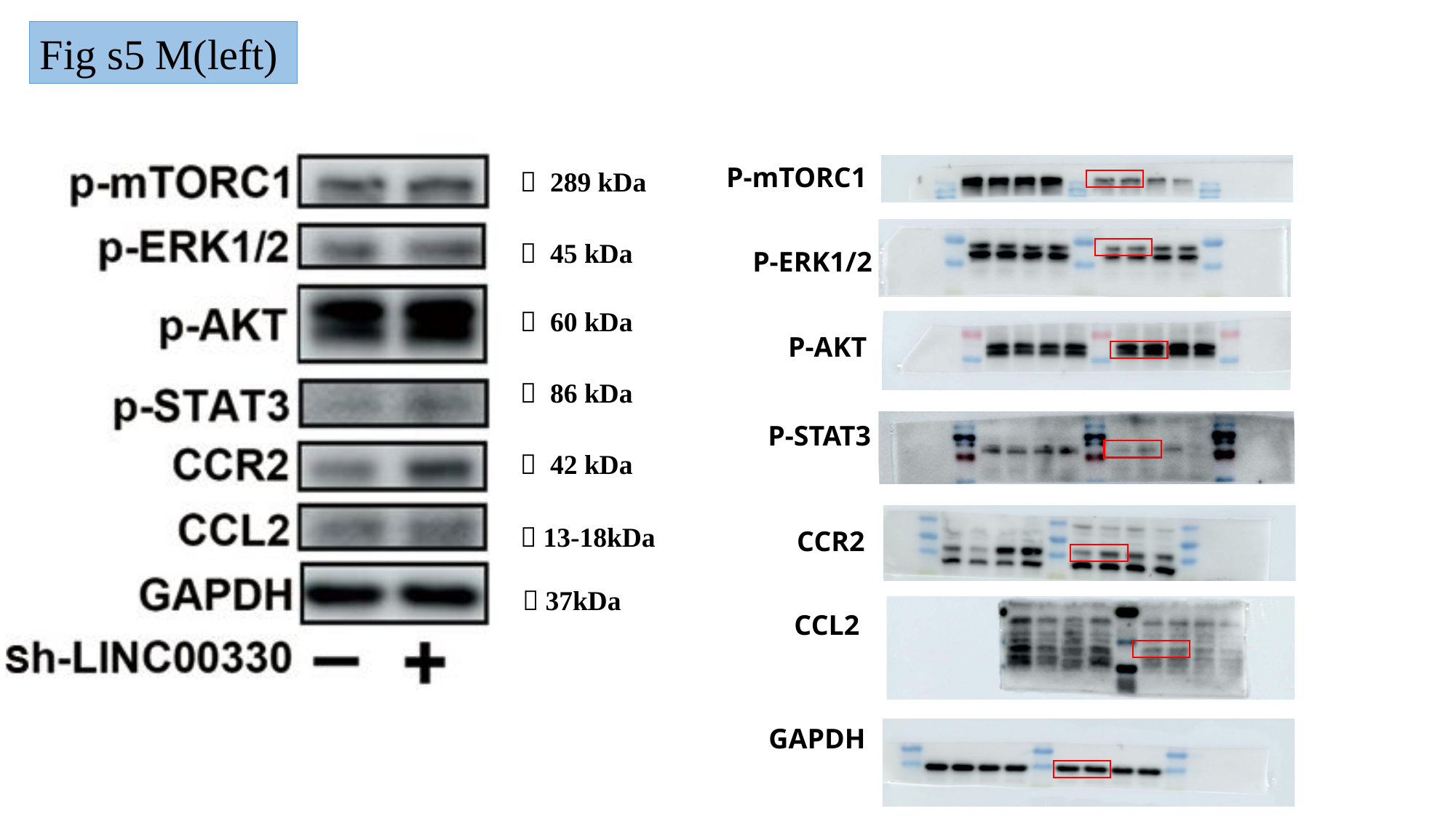

Fig s5 M(left)
P-mTORC1
〜 289 kDa
〜 45 kDa
P-ERK1/2
〜 60 kDa
P-AKT
〜 86 kDa
P-STAT3
〜 42 kDa
〜13-18kDa
CCR2
〜37kDa
CCL2
GAPDH

## Slide 56
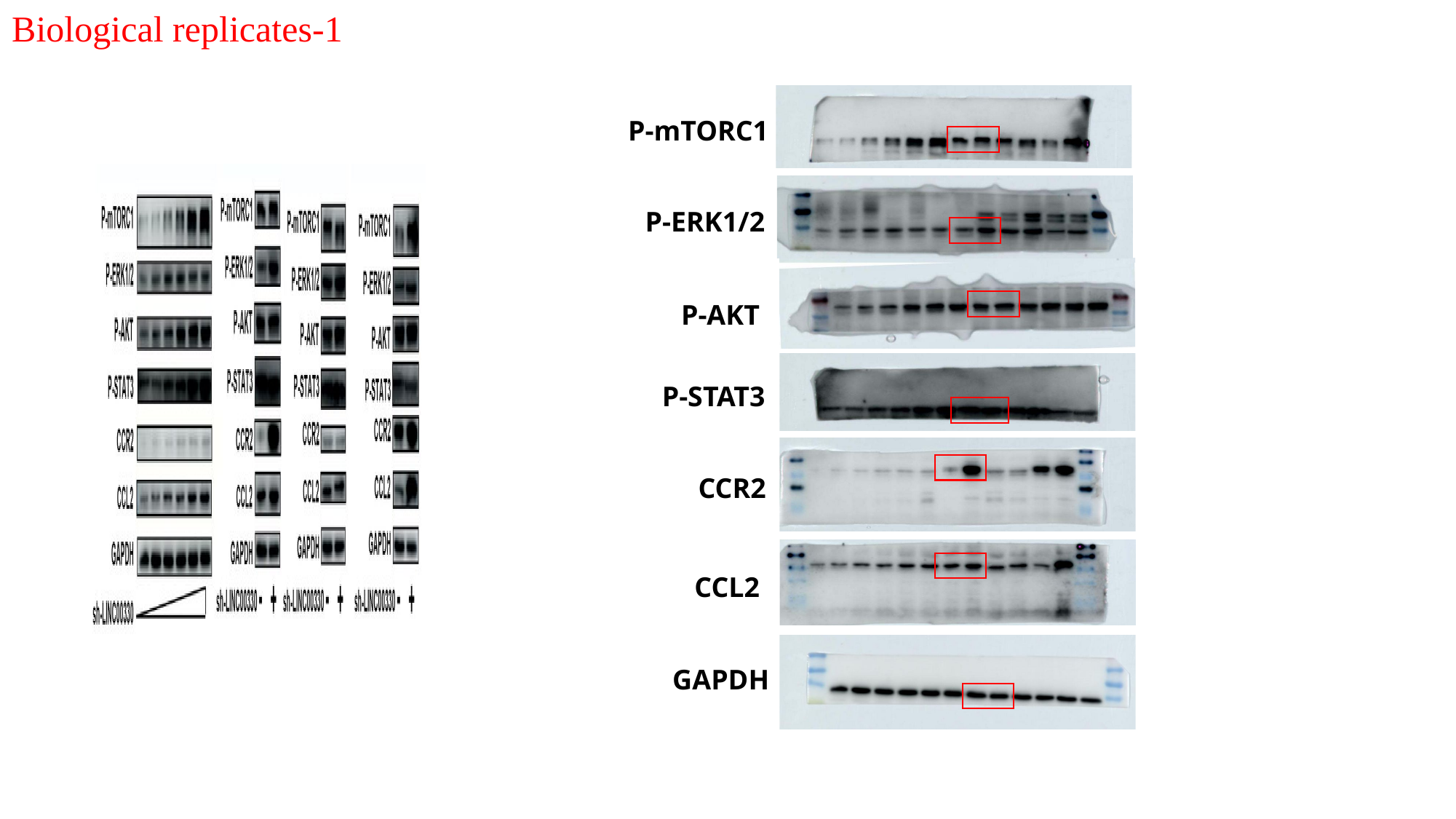

Biological replicates-1
P-mTORC1
P-ERK1/2
P-AKT
P-STAT3
CCR2
CCL2
GAPDH

## Slide 57
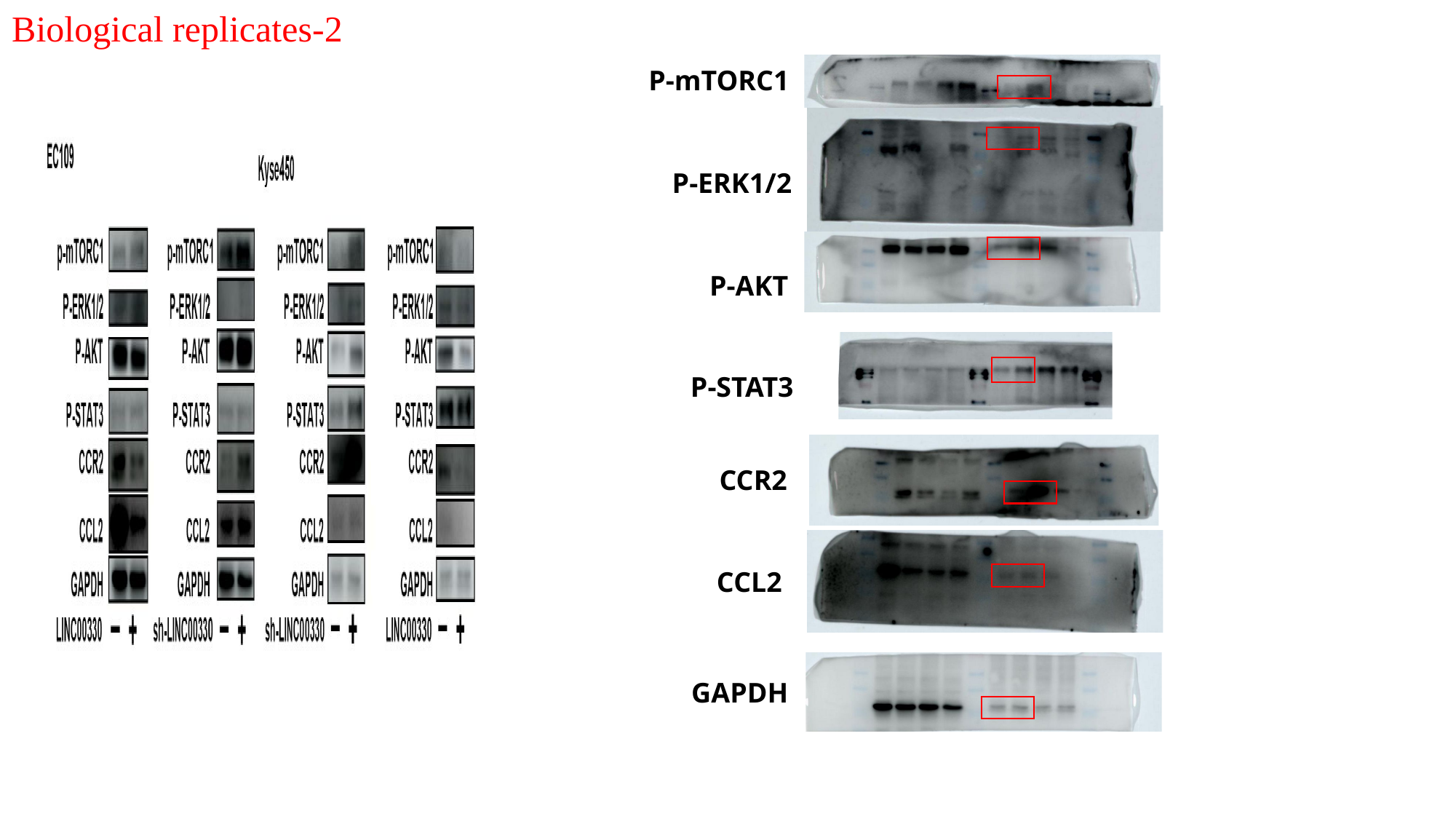

Biological replicates-2
P-mTORC1
P-ERK1/2
P-AKT
P-STAT3
CCR2
CCL2
GAPDH

## Slide 58
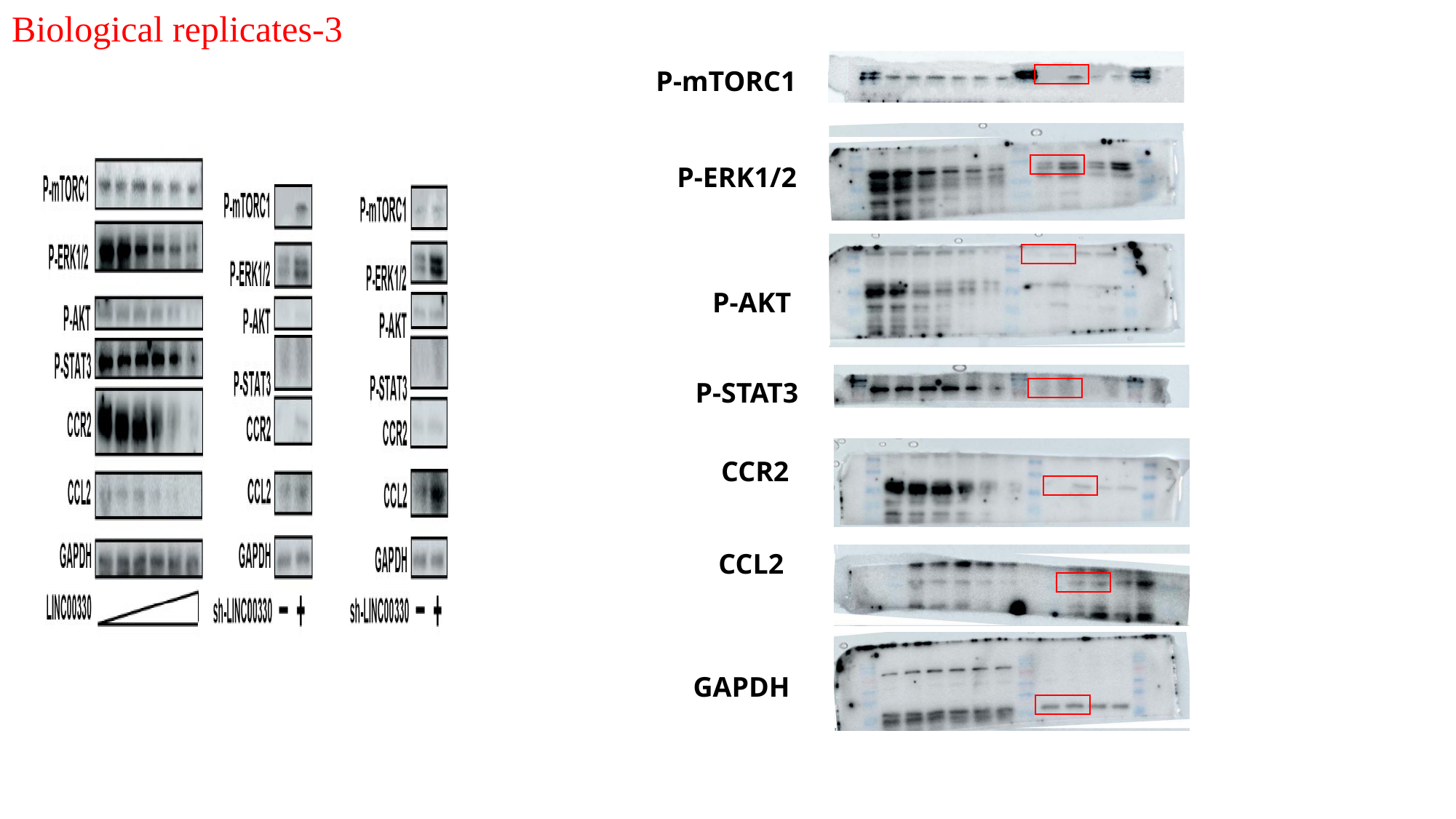

Biological replicates-3
P-mTORC1
P-ERK1/2
P-AKT
P-STAT3
CCR2
CCL2
GAPDH

## Slide 59
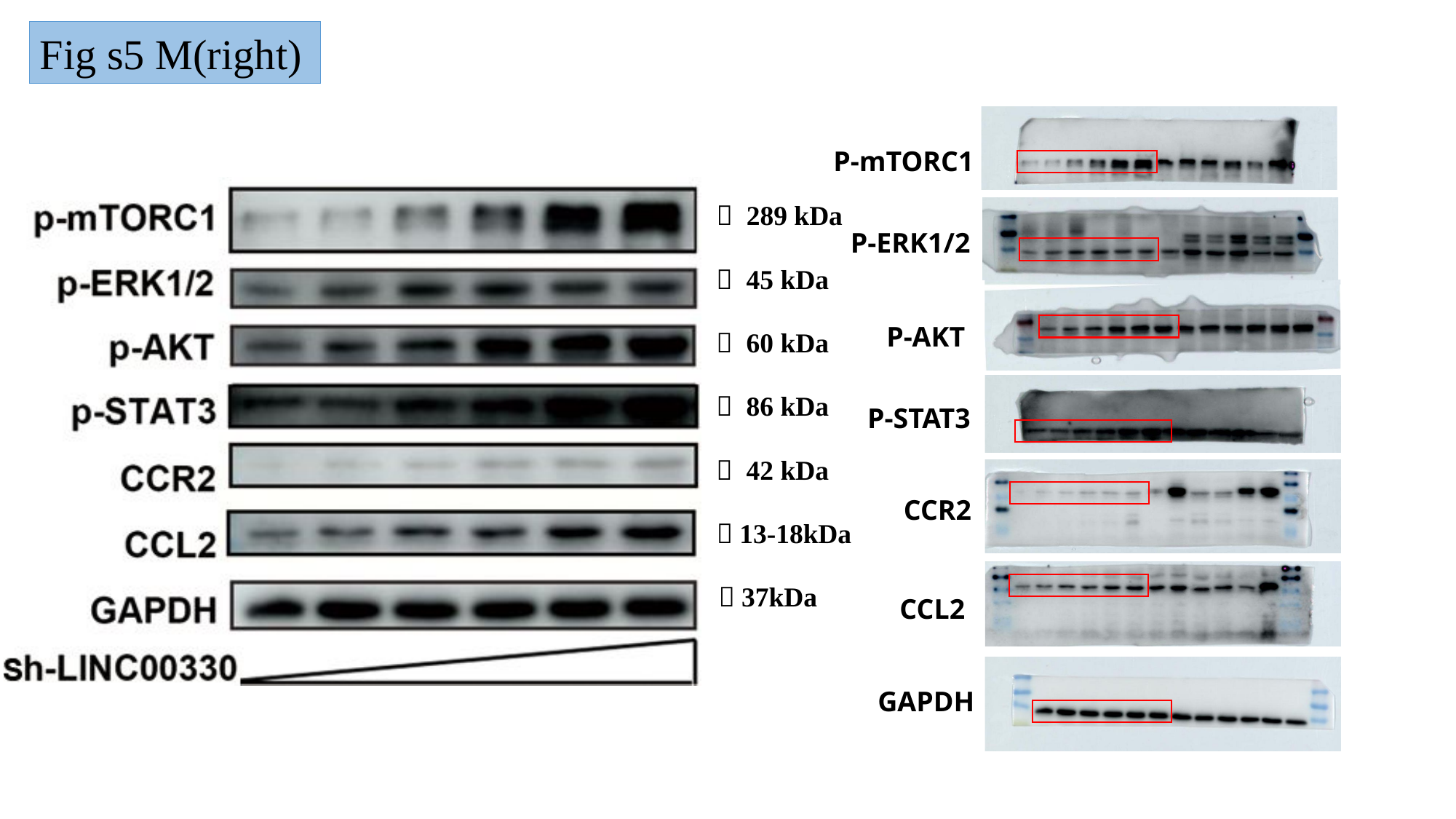

Fig s5 M(right)
P-mTORC1
〜 289 kDa
P-ERK1/2
〜 45 kDa
P-AKT
〜 60 kDa
〜 86 kDa
P-STAT3
〜 42 kDa
CCR2
〜13-18kDa
〜37kDa
CCL2
GAPDH

## Slide 60
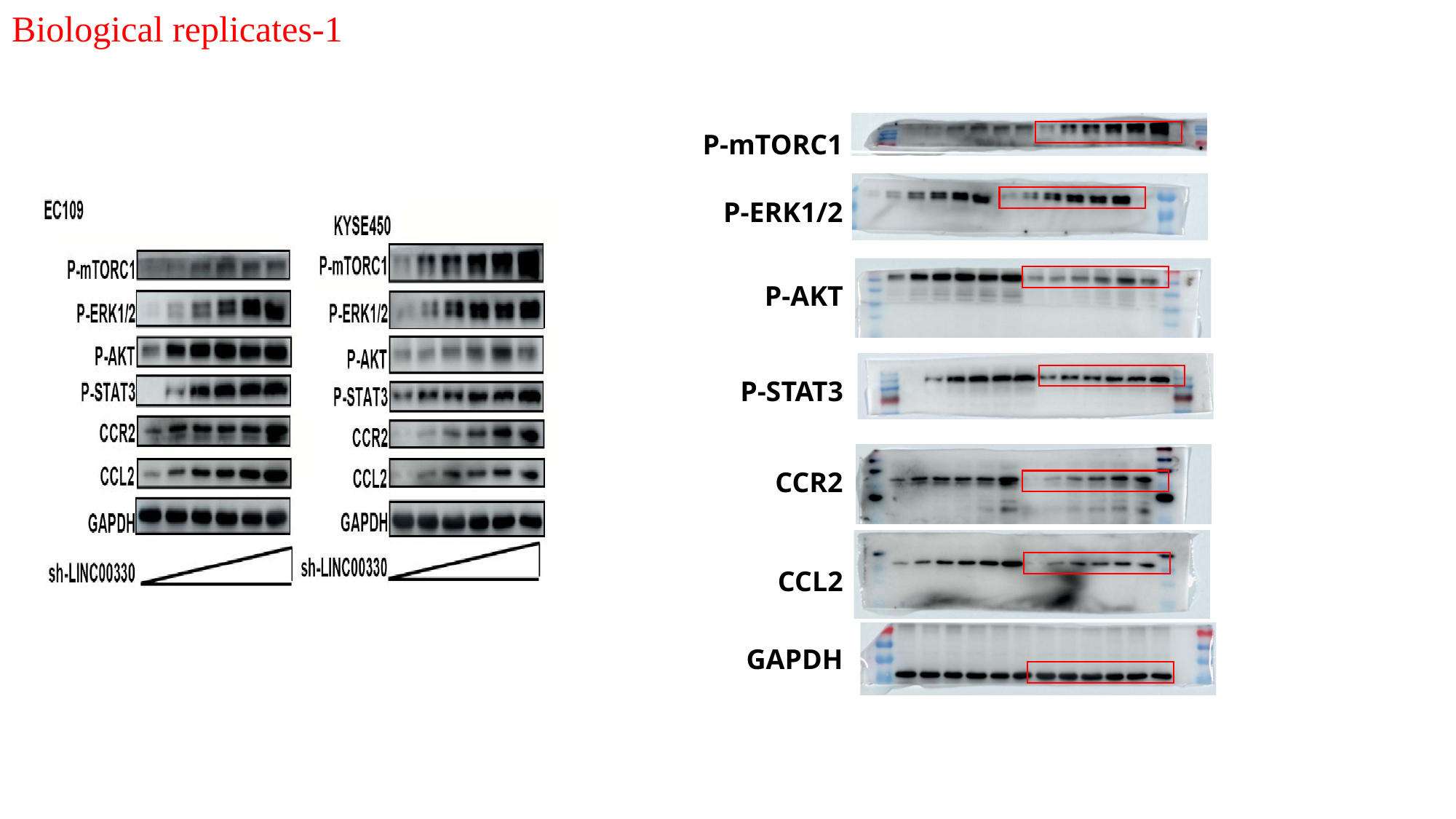

Biological replicates-1
P-mTORC1
P-ERK1/2
P-AKT
P-STAT3
CCR2
CCL2
GAPDH

## Slide 61
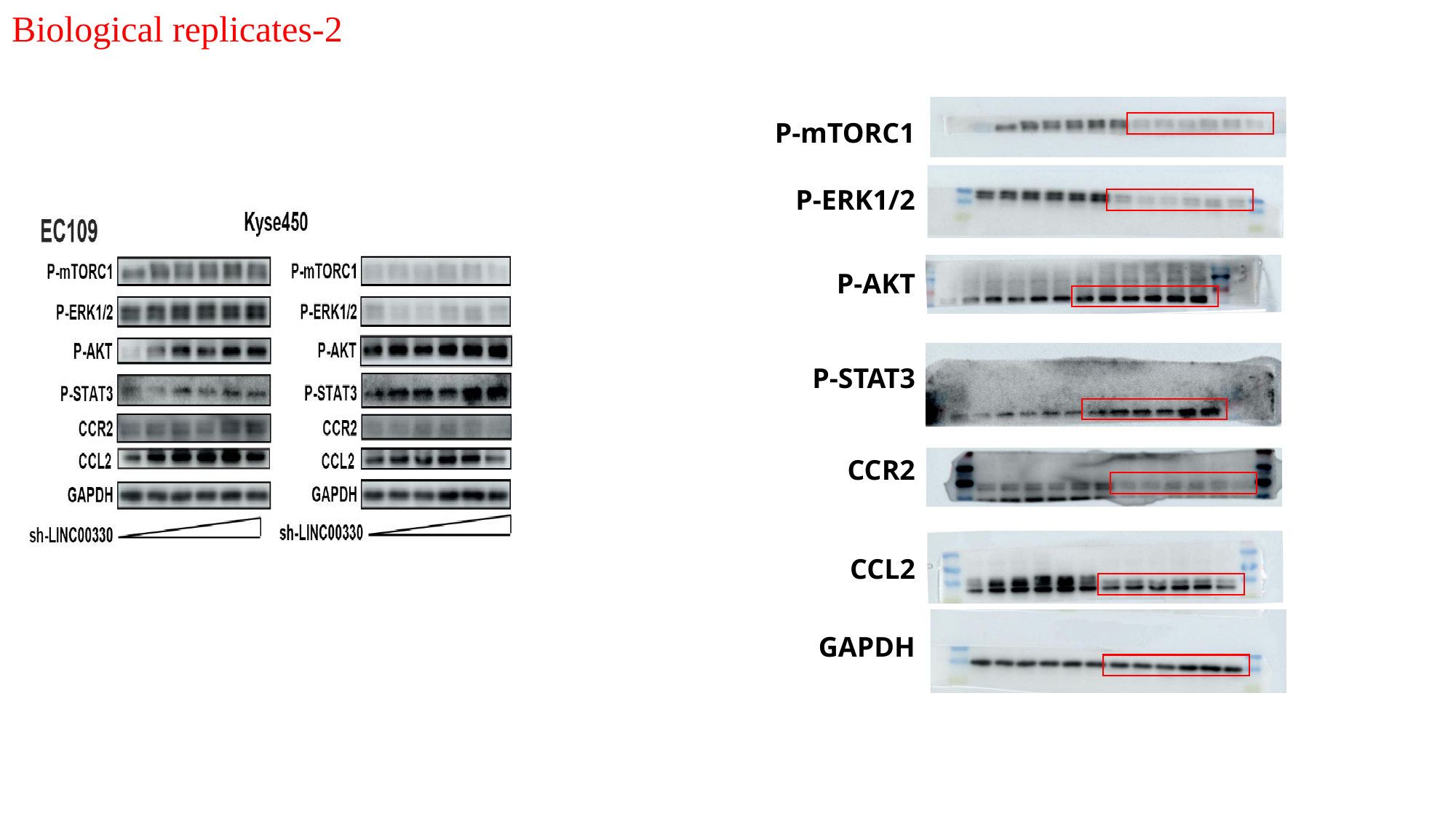

Biological replicates-2
P-mTORC1
P-ERK1/2
P-AKT
P-STAT3
CCR2
CCL2
GAPDH

## Slide 62
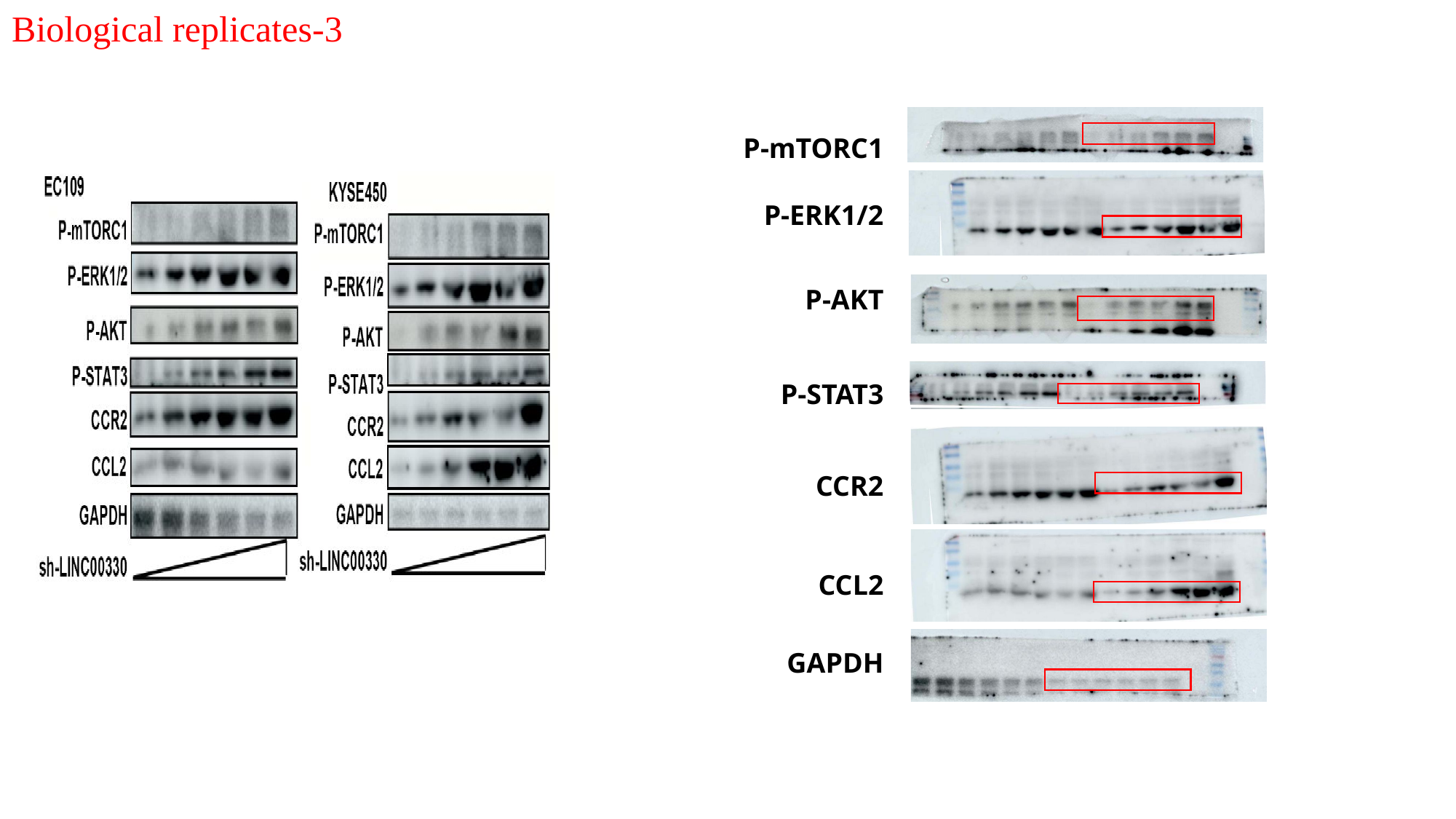

Biological replicates-3
P-mTORC1
P-ERK1/2
P-AKT
P-STAT3
CCR2
CCL2
GAPDH

## Slide 63
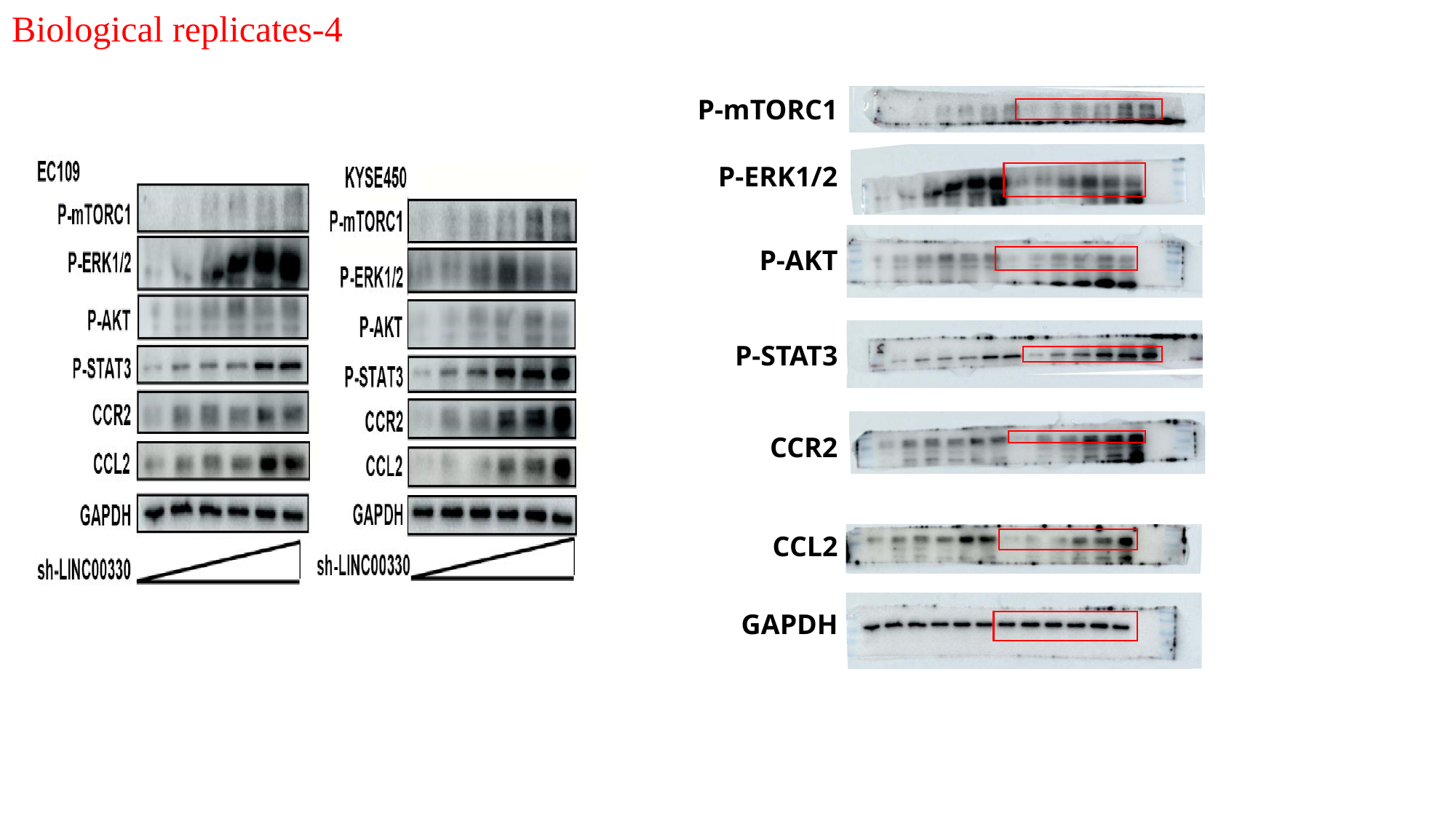

Biological replicates-4
P-mTORC1
P-ERK1/2
P-AKT
P-STAT3
CCR2
CCL2
GAPDH

## Slide 64
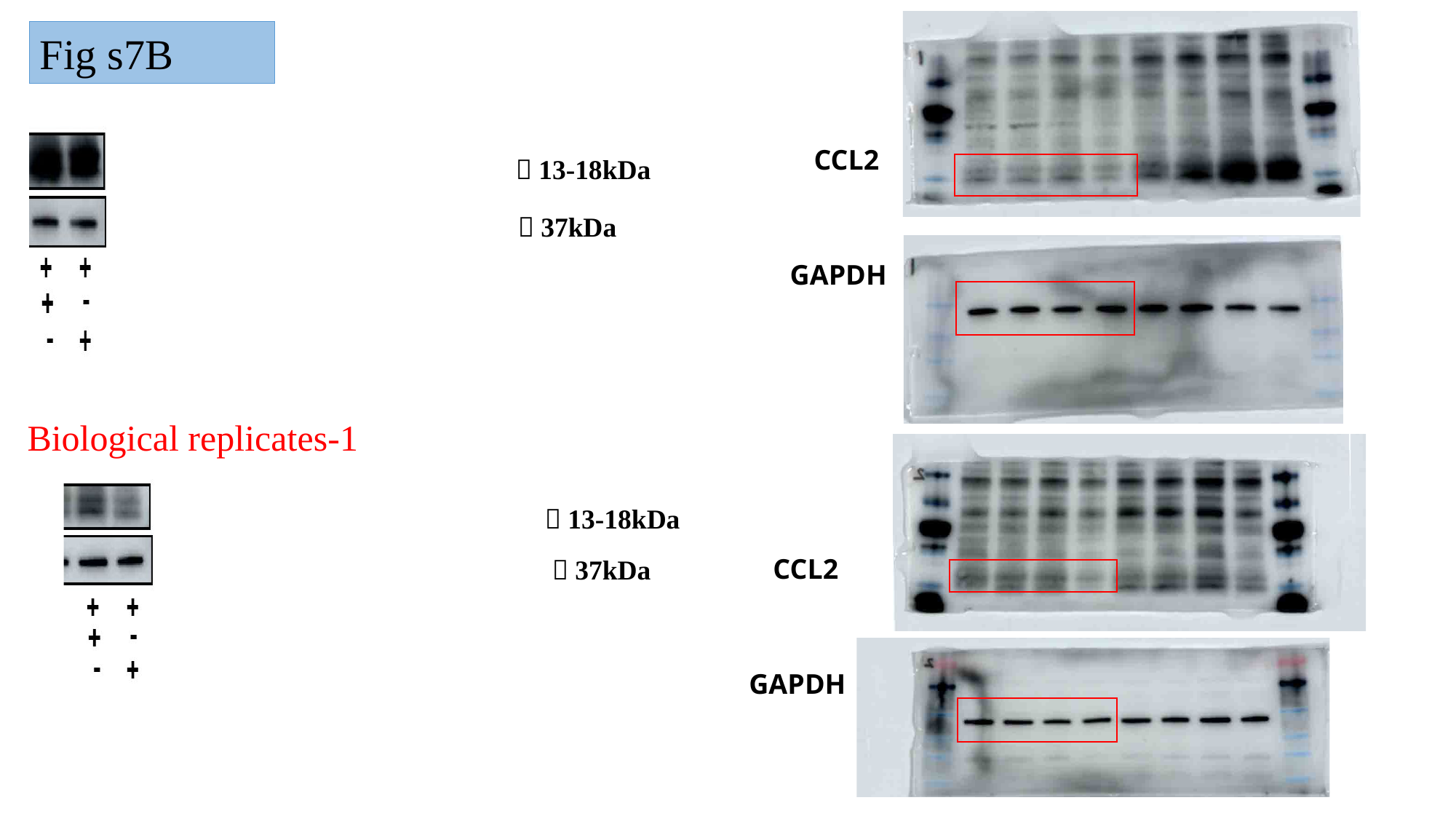

Fig s7B
CCL2
〜13-18kDa
〜37kDa
GAPDH
Biological replicates-1
〜13-18kDa
CCL2
〜37kDa
GAPDH

## Slide 65
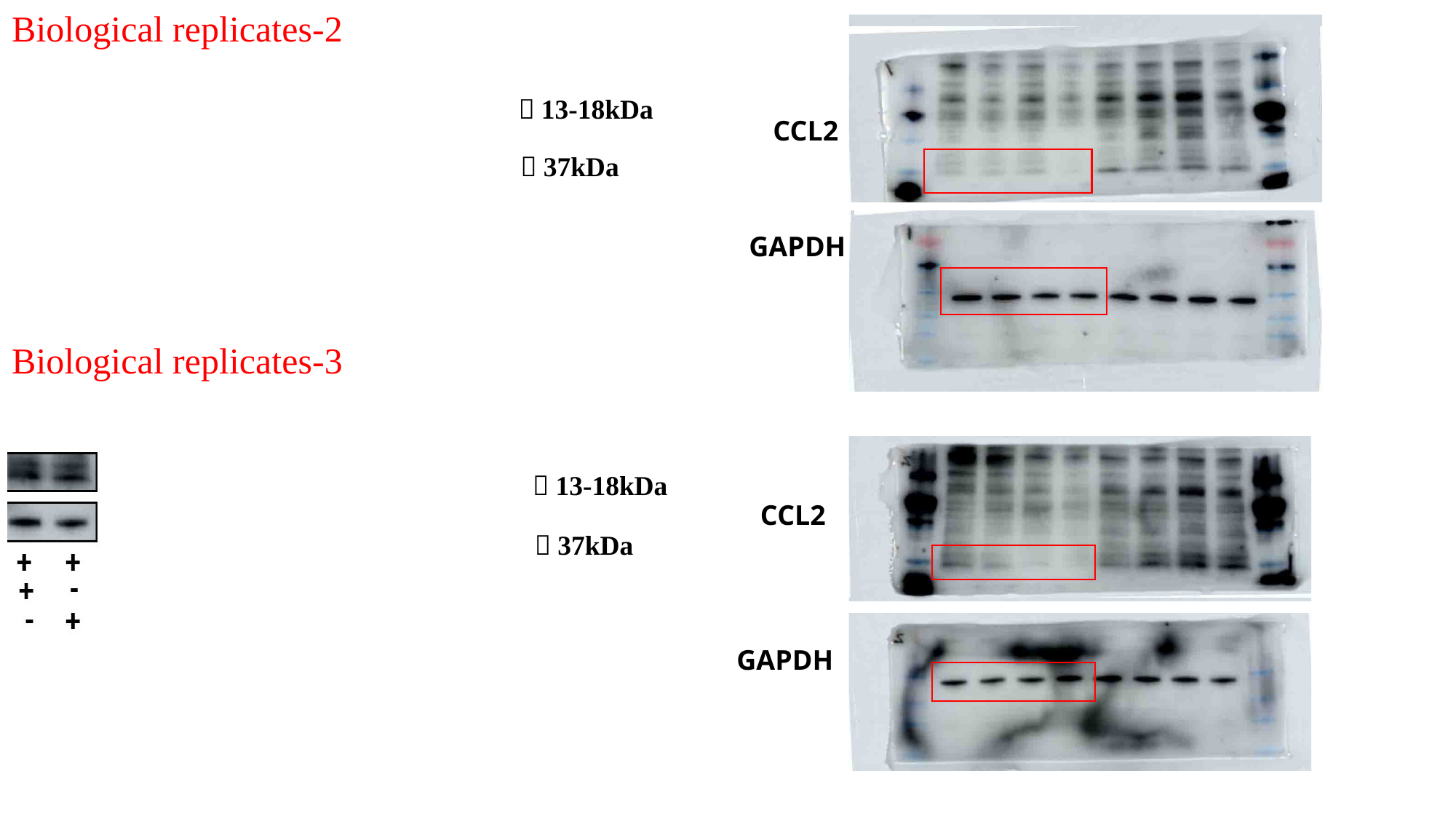

Biological replicates-2
〜13-18kDa
CCL2
〜37kDa
GAPDH
Biological replicates-3
〜13-18kDa
CCL2
〜37kDa
GAPDH

## Slide 66
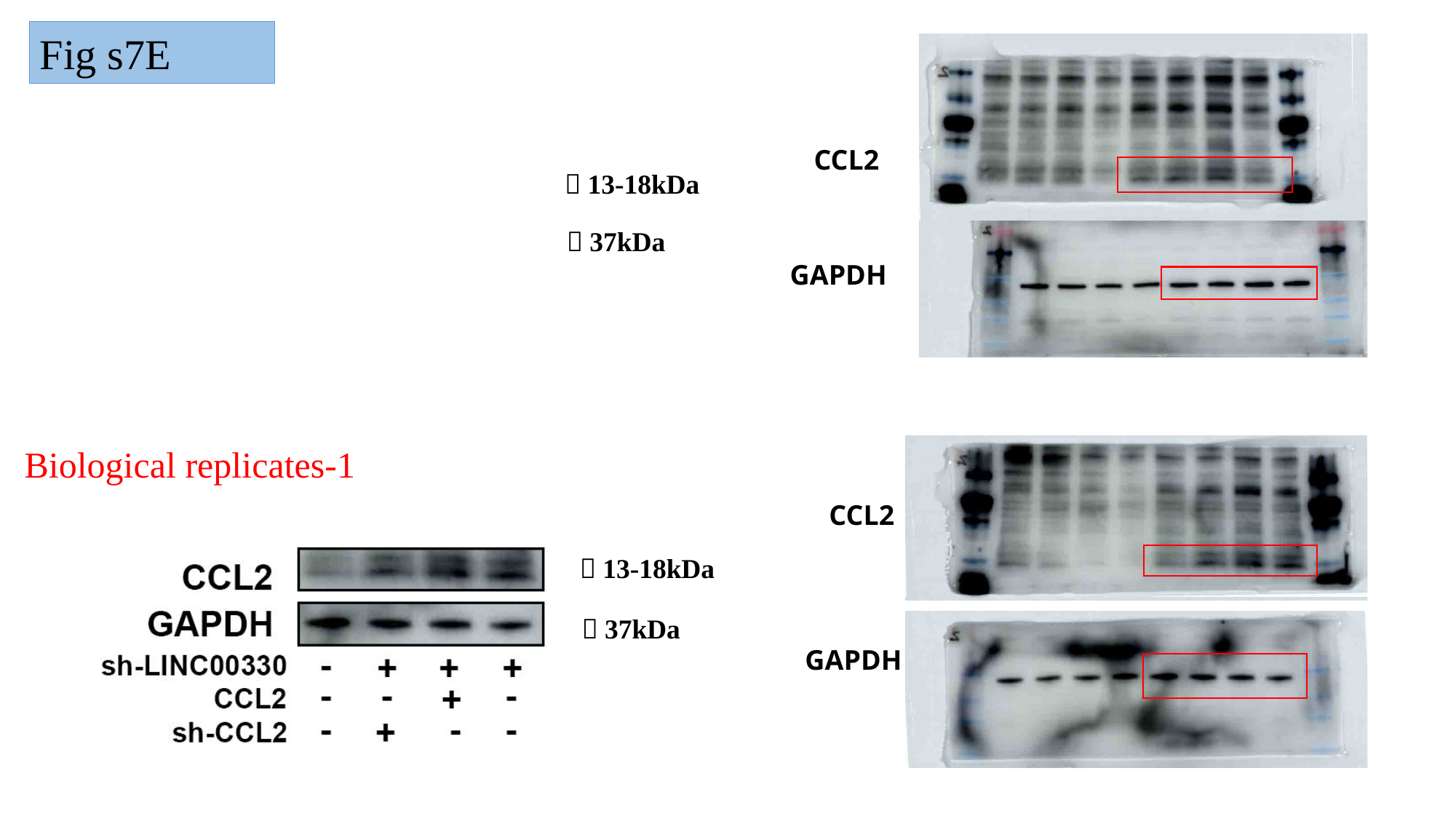

Fig s7E
CCL2
〜13-18kDa
〜37kDa
GAPDH
Biological replicates-1
CCL2
〜13-18kDa
〜37kDa
GAPDH

## Slide 67
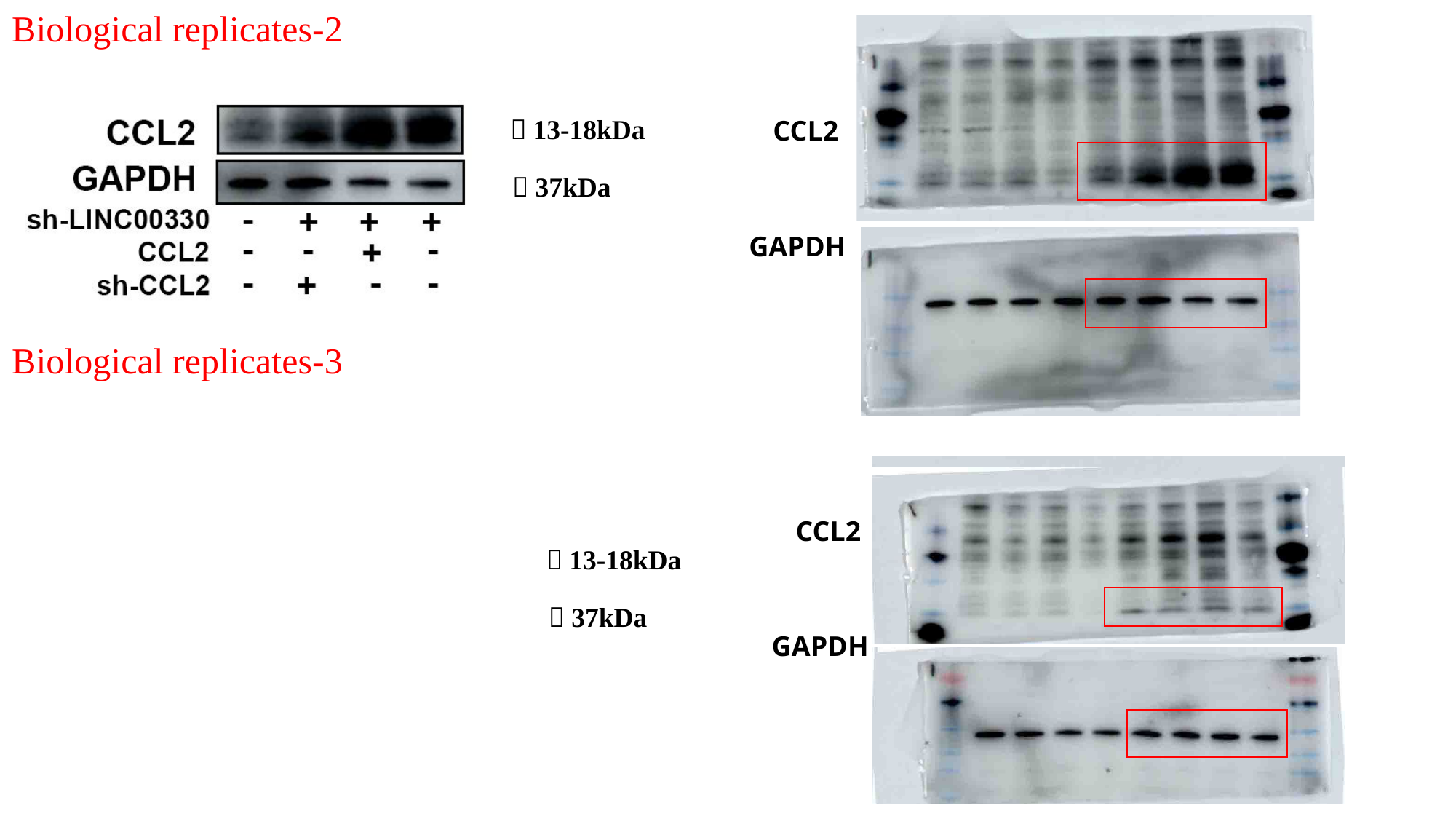

Biological replicates-2
〜13-18kDa
CCL2
〜37kDa
GAPDH
Biological replicates-3
CCL2
〜13-18kDa
〜37kDa
GAPDH
